# The zinc transporter Zip14 (SLC39a14) affects Beta-cell Function: Proteomics, Gene expression, and Insulin secretion studies in INS-1E cells

**DOI:** 10.1038/s41598-019-44954-1

**Published:** 2019-06-13

**Authors:** Trine Maxel, Kamille Smidt, Charlotte C. Petersen, Bent Honoré, Anne K. Christensen, Per B. Jeppesen, Birgitte Brock, Jørgen Rungby, Johan Palmfeldt, Agnete Larsen

**Affiliations:** 1 Department of Biomedicine, Faculty of Health, Aarhus University, Aarhus, Denmark; 2 Department of Clinical Medicine, Faculty of Health, Aarhus University, Aarhus, Denmark; 3 Department of Endocrinology and Internal Medicine, Aarhus University Hospital, Aarhus, Denmark; 4 Steno Diabetes Center, Copenhagen, Denmark; 5 Department of Endocrinology I, Bispebjerg University Hospital, Copenhagen, Denmark; 6 Research Unit for Molecular Medicine, Aarhus University Hospital, Aarhus, Denmark

**Keywords:** Cell biology, Diabetes

## Abstract

Insulin secretion from pancreatic beta-cells is dependent on zinc ions as essential components of insulin crystals, zinc transporters are thus involved in the insulin secretory process. Zip14 (SLC39a14) is a zinc importing protein that has an important role in glucose homeostasis. Zip14 knockout mice display hyperinsulinemia and impaired insulin secretion in high glucose conditions. Endocrine roles for Zip14 have been established in adipocytes and hepatocytes, but not yet confirmed in beta-cells. In this study, we investigated the role of Zip14 in the INS-1E beta-cell line. *Zip14* mRNA was upregulated during high glucose stimulation and Zip14 silencing led to increased intracellular insulin content. Large-scale proteomics showed that Zip14 silencing down-regulated ribosomal mitochondrial proteins, many metal-binding proteins, and others involved in oxidative phosphorylation and insulin secretion. Furthermore, proliferation marker Mki67 was down-regulated in *Zip14* siRNA-treated cells. In conclusion, *Zip14* gene expression is glucose sensitive and silencing of Zip14 directly affects insulin processing in INS-1E beta-cells. A link between Zip14 and ribosomal mitochondrial proteins suggests altered mitochondrial RNA translation, which could disturb mitochondrial function and thereby insulin secretion. This highlights a role for Zip14 in beta-cell functioning and suggests Zip14 as a future pharmacological target in the treatment of beta-cell dysfunction.

## Introduction

Pancreatic beta-cell dysfunction is a key feature of type 2 diabetes (T2D)^1^. Zinc is essential for insulin storage^2^, and several zinc transporters of the SLC30A family, which orchestrate the intracellular distribution of free zinc, have been associated with T2D pathology^3–9^. Genome-wide association studies link a common polymorphism within the zinc transporter *ZNT8*, exporting zinc to insulin granules^6,7^, with an increased risk of T2D^8^. Similarly, ZNT5 is highly present at insulin secretory granules^9^, and Znt3 and Znt7 play a functional role in insulin secretion^3–5^. Moreover, the reduced levels of circulating zinc found in individuals with T2D indicate a systemic zinc dyshomeostasis in T2D^10,11^.

Intracellular free zinc is found in picomolar concentrations^12,13^, but it is ubiquitously involved in cell proliferation, apoptotic pathways, and insulin signaling, e.g. through regulation of tyrosine phosphatase activity^14–17^. Within the normal physiological range, free zinc is protective against oxidative stress^12^, whereas adverse effects of zinc are observed when it accumulates in mitochondria, leading to mitochondrial dysfunction and increased production of reactive oxygen species^18,19^. Mitochondrial function is critical for normal insulin secretion: mitochondrially generated ATP causes the closure of ATP-dependent potassium channels, leading to depolarization of the cell membrane, Ca^2+^ influx, and release of insulin by exocytosis^20^.

To maintain intracellular zinc homeostasis, the concentration and distribution of zinc are tightly regulated by zinc transporters of the SLC30A (ZNTs) and Zrt and Irt-Like Protein (ZIP) family, and by metal-binding metallothioneins (MTs)^21^. Recent studies indicate an important role for ZIP transporters in the function of beta-cells. The ZIP transporters Zip4, Zip6, and Zip7 stimulate insulin secretion and ZIP6 has an anti-apoptotic function in GLP treatment^22,23^.

Recently, ZIP14 has emerged as a transport protein with a general impact on glucose metabolism, growth, and adipose tissue functioning^24–27^. ZIP14 is located mainly at the plasma membrane, where it functions as a Zn^2+^ importing protein, but it has also been implicated in the transport of Fe^2+^ into liver and pancreas in conditions involving iron overload^28–30^. The Zip14 knockout mouse is characterized by hyperinsulinemia, however, with signs of impaired insulin secretion upon high glucose stimulation, as well as impaired hepatic gluconeogenesis and glycolysis^26,31^. An increased insulin content is observed in beta-cells of Zip14 knockout animals^26^, alongside decreased proliferative and differentiation potential of several cell types^24,25,27,32^. *ZIP14* is highly expressed in human liver and pancreas^33^, and it is present in both the alpha- and beta-cells of the human pancreas when investigated by immunofluorescence^34^. In human type 2 diabetic individuals, signs of a low pancreatic *Zip14* expression levels are observed^22^.

In this study, the INS-1E model of insulin-producing beta-cells was used. INS-1E cells originate from rat insulinoma cells and are characterized by a stable glucose-induced insulin secretion with a dose-related response similar to isolated rat islets^35,36^. The aim of this study was to confirm the presence of Zip14 at the protein level in the INS-1E cells and to characterize its regulation and the role of Zip14 in the beta-cell, with regard to the regulation of insulin processing, cell survival, and, using a large-scale proteomic approach, the regulation of other proteins.

## Materials and Methods

### Cultivation of INS-1E cells

INS-1E cells, provided by Prof. Wollheim and Prof. Maechler, Switzerland, were cultivated as originally described^35,36^, in a 5% CO_2_-containing atmosphere in RPMI 1640 medium (Lonza, Verviers, Belgium) containing 11 mM glucose, 2 mM glutamine, 10% heat-inactivated fetal bovine serum, 100 U/ml penicillin, 100 g/ml streptomycin, and 50 μM beta-mercaptoethanol. Cell passage numbers below 100 were used, and the capacity for glucose-induced insulin secretion was confirmed prior to experiments.

### *Zip14, Znt8*, and *Insulin (Ins)* mRNA expression measurement using PrimeFlow

PrimeFlow experiments were undertaken using PrimeFlow RNA Assays (eBioscience, San Diego, CA, USA), based on Fluorescent *in situ* hybridization (FISH) principle.

INS-IE cells were cultivated in RPMI 1640 medium (11 mM glucose) for 3 d and subsequently stimulated with 5, 11, 16 or 24 mM glucose for 24 h (4–6 replicates). The PrimeFlow procedures were performed according to the manufacturer’s protocol using 1–2 million cells stained with viability dye (1 µl/ml; Fixable Viability Dye eFluor 450, eBioscience).

Samples were analyzed using a LSR Fortessa (BD Biosciences, San Jose, CA, USA) equipped with 405, 488, 561, and 640 nm lasers. As control samples, unstained cells cultured in 5, 11, 16, and 24 mM glucose were used for autofluorescence measurements as well as positive control beta-actin probed cells (part of the PrimeFlow RNA Assay), conjugated with AlexaFluor 488, AlexaFluor 647, and AlexaFluor 750. Fluorescence minus one (FMO) controls and single color samples of *Insulin*, *Zip14*, *Zip8*, and the fixable viability dye eFluor 450, were included for flow cytometry gating and fluorescence compensation. For gating strategy please refer to Supplementary Material 1.

For each sample, 300,000 cells were collected and data were analyzed using FlowJo software (v. 10.0.8, FlowJo LLC, Ashland, OR, USA). Data were given as the median fluorescent value for the 300,000 cells collected for each sample. Data are presented as the mean median fluorescence value ± SEM, and groups were compared using the Kruskal-Wallis test, followed by Dunn’s *post-test* where appropriate (GraphPad Prism 5, San Diego, CA, USA; level of significance, p < 0.05).

### Transfection procedure

Cells (100,000) were seeded into 24-well plates and grown in supplemented RPMI 1640 medium (11 mM glucose), as described, but without any antibiotics. Transfection procedure were performed as previously described^5^, using siRNA targeting Zip14 (ON-TARGET plus Rat Slc39a14 siRNA SMARTpool, Thermo Fischer Scientific) and, as a control, non-targeting siRNA (ON-TARGET plus Non-targeting siRNA, Thermo Fischer Scientific). The target sequences of the ZIP14 siRNA were as follows: GUAUAUUGCUCUAGCCGAU, GCUCAAAGGGGUUCGAUAU, CCACAACUUCAGUGAGCGA, and GAGCUGGGAGACUUCGUUA. The transfection efficiency was assessed by measurement of mRNA expression levels in all experiments and investigated once at the protein level using targeted proteomic analysis, as described below.

### RNA extraction and real-time PCR

RNA was extracted using Qiagen RNAeasy Mini kits (VWR) and evaluated spectrophotometrically and by agarose gel electrophoresis. RNA was reverse transcribed into cDNA using the ImProm-IITM Reverse Transcription System (Promega, Madison, Wisconsin, USA) and oligo (dT)15 primers. cDNA was checked for genomic DNA contamination by PCR analysis using the Qiagen HotStarTaq Master Mix Kit (VWR) and an intron-spanning primer-set for beta-actin (TAC, Copenhagen, Denmark). Quantitative real-time PCR was performed in duplicate with IQ SYBR Green Supermix (Roche applied Science, Germany) in a MyiQ Single-Color Real-time PCR detection system (Bio-Rad, Denmark). Results were analyzed with iQ Optical System Software, V. 2.0. Starting quantities were calculated using a standard curve. Values were normalized to the geometric mean of up to three house-keeping genes (*Beta-actin*, *Cyclophilin A*, and *HSP90ab1*). The geNorm method (geNorm 3.5) was used to confirm the stability of the house-keeping genes^37^.

Transfection efficiency was checked by real-time PCR in all experiments by comparing gene expression of *Zip14* in *Zip14* siRNA-treated cells vs. control siRNA-treated cells (n = 6 per group). Gene expression of *insulin (Ins)*, *Bax*, *Bcl-2*, metallothionein-1A (*Mt1a*), metallothionein-3 (*Mt3*), *Znt1*, *Znt3*, *Znt5*, *Znt8*, *Zip6*, *Zip9*, and *Zip13* was measured after transfection of INS-1E cells, comparing *Zip14* siRNA- and control siRNA-treated cells (n = 6 per group). The primers used and gene ID numbers are listed in Supplementary Material 2.

Data are presented ± SEM and were compared using the Mann-Whitney U-test (GraphPad Prism 5; level of significance, p < 0.05).

### Targeted and large scale proteomic studies

Transfected INS-1E cells (*Zip14* siRNA and control siRNA) were harvested and lysed. Protein concentrations were measured using Pierce BCA Protein Assay Reagent kits (Bie & Berntsen A/S, Denmark). See Supplementary Material 3 for details on proteomics methods.

In targeted analysis, using selected reaction monitoring mass spectrometry (MS), the protein level of Zip14 was measured by specific targeting of a unique Zip14 peptide (SEFQEFCPTILQQLDSR) sequence using an isotopically labeled peptide standard of the same sequence (JPT Technology, Berlin, Germany). Five replicate samples of Zip14 siRNA- and control siRNA transfected cells were analyzed as previously described^38^. Briefly, proteins were separated by SDS-PAGE, in-gel trypsin digested and resulting peptides were C18 purified and analyzed by nano-liquid chromatography (LC)-MS/MS consisting of an Ultimate 3000 LC (Thermo Fisher Scientific) coupled to a TSQ Vantage triple quadrupole mass spectrometer (Thermo Fisher Scientific, Waltham, MA, USA). Raw data files from the LC-MS/MS analysis were imported into Skyline software^39^ and the ratios between the endogenous (light) and internal standard (heavy) peptides were calculated. All samples were analyzed twice by Triple Q MS. The mean ratio (endogenous Zip14 specific peptide/heavy labeled standard peptide) for each group was calculated using Excel, and the groups were compared using the Mann-Whitney U-test (Microsoft Excel and GraphPad Prism 5; level of significance, p < 0.05).

By the use of large scale proteomics the regulation of cellular proteins was evaluated following Zip14 silencing. An isobaric tag was added to 70 µg of protein from *Zip14* siRNA- and control siRNA-treated cells (n = 4 replicates) using a tandem mass tag (TMT) 10plex Mass Tag Labeling Kit (Applied Biosystems, Foster City, CA, USA), according to the manufacturer’s protocol. Samples were subsequently purified by strong cation exchange, fractionated by isoelectric focusing, and peptides were extracted and C18 purified, all as previously described^40^. Samples were analyzed twice by the use of a nano-LC (Ultimate 3000, Dionex) coupled to a mass spectrometer (Orbitrap Fusion, Thermo Fisher Scientific, Bremen, Germany) through an EASY-Spray nano-electrospray ion source (Thermo Scientific). Data files were merged and analyzed using Mascot v. 2.5.1 (Matrix Science, London, UK) in Proteome Discoverer 2.1 (Thermo Scientific). The MS data were searched against the SwissProt *Rattus* database downloaded in May 2016. Normalization to the summed intensities of the tandem mass tag signal was applied to compensate for possible variation in the starting material. The false discovery rate (FDR) criterion for peptides was q < 0.01. Only proteins with an identification score ≥ 30 (corresponding to a protein identification significance of 0.001) were considered, as well as proteins with at least three quantitative scans and one protein unique peptide, yielding 3431 proteins. Mean protein ratios were calculated for each protein as the mean abundance in the four *Zip14* siRNA-treated samples divided by the mean abundance in control siRNA-treated samples. A significant fold change was defined as 2× global standard error of the 3431 proteins (2 × 0.068 = 0.136). FDR was calculated from p-values (estimated by Student’s t-test) using the Benjamini and Hochberg’s method^41^. A FDR < 0.05 was used. Fifty-two proteins met the fold-change and FDR criteria. For gene ontology analysis, to obtain protein groups, wider criteria, defined as a p value < 0.05 and a significant fold change, were applied, yielding 121 proteins. For ontology analysis, the Functional Annotation tool from the Database for Annotation Visualization and Integrated Discovery (DAVID) was used (DAVID 6.8 Beta; https://david-d.ncifcrf.gov/). The total list of the 3431 proteins found within the samples was selected as background. An enrichment score (ES) ≥ 1.3, equal to 0.05 on a non-log scale, was set as the level of significance^42^. Three protein lists were analyzed for functional enrichment; one included both up- and downregulated proteins and two consisted of only either down- or upregulated proteins. Mitocarta 2.0 compendium was used to identify mitochondrial proteins within our dataset of the 3431 quantified proteins^43^, and data from KEGG pathway database was applied to filter out proteins of the central metabolic pathways^44^.

Further details and technical descriptions on proteomic analyses are found in Supplementary Material 3. One sample was excluded in both targeted and large scale analysis due to an outlying calculated Zip14 level (Zip14 siRNA treated sample).

### Western blot of chromogranin a (chga)

The same samples of transfected INS-1E cells (*Zip14* siRNA and control siRNA) were used as in the proteomic experiments.

Protein samples were dissolved in sample buffer (Laemmli buffer, Biorad, Copenhagen, Denmark) and DTT (dithiothreitol) and treated for 5 minutes at 95 °C. 5 μg of protein were used. Protein samples were separated on a 4–12% CRIT XT BIS-TRIS GEL (Biorad) and transferred to a PVDF membrane (Biorad). The membranes were blocked with 5% fat-free milk in tris-buffered saline containing 0.1% Tween-20 for 1.5 h. Primary antibody were added and membranes were incubated overnight at 4 °C, where after a horseradish peroxidase (HRP)-conjugated secondary antibody were added and incubated for 2 h at room temperature. Visualization were performed using Clarity ECL or Clarity ECL max (Biorad).

Primary Antibodies: Chromogranin A mouse monoclonal antibody, dilution 1:6000 (Proteintech), b-actin mouse monoclonal antibody, dilution 1:300000 (Sigma Aldrich, St Louis, Missouri, USA). Secondary antibody: donkey anti-mouse IgG-HRP, dilution 1:6000 (Santa Cruz Biotechnology, Dallas, Texas, USA). Due to lack of sample material 9 samples were included for western blot. B-actin was used as internal control. The intensities of Chga were normalized to the corresponding intensity of the internal control (b-actin) in each lane to obtain a normalized quantification.

The Mann-Whitney U-test was used to compare *Zip14* siRNA-treated cells with control cells (GraphPad Prism 5; level of significance, p < 0.05).

### Intracellular and secreted insulin measurements

To evaluate the ability of the transfected cells to respond to glucose stimuli as well as to quantify the insulin secretion capacity, the cells were exposed to different glucose concentrations, ranging from low to high glucose compared to the basic condition of 11 mM glucose. Cells were glucose stimulated in a period of 24 h. Transfected cells (*Zip14* siRNA and control siRNA) were stimulated with RPMI 1640 medium containing either 5 mM (lower than average glucose/slightly hypoglycemic condition), 16 mM (slightly elevated glucose), or 24 mM (high glucose exposure) glucose (n = 6 replicates). Prior to insulin and protein measurements, growth media was replaced by Krebs-Ringer bicarbonate HEPES buffer containing the same glucose concentrations for 2 h, after which media, destined for secreted insulin measurement, were collected, and cells were harvested for protein and intracellular insulin measurements, as previously described in detail^5^. Protein measurements were performed using the BCA Protein Assay Reagent Kit. One sample was excluded due to a significant outlying protein concentration (control siRNA sample at 16 mM). Secreted and intracellular insulin measurements were performed using Rat Insulin Elisa kits from DRG Diagnostics (VWR, Denmark), following the manufacturer’s instructions. For each sample, secreted and intracellular insulin measurements were normalized to the protein content.

Mean intracellular and secreted insulin values are presented as (µIU/µg of protein) ± SEM as well as the mean ratio of secreted insulin/intracellular insulin ± SEM. Groups were compared using the Mann-Whitney U-test (GraphPad Prism 5; level of significance, p < 0.05).

### Measurement of viability

Measurement of viability was performed twice on transfected INS-1E cells (*Zip14* siRNA/control siRNA) stimulated for 24 h with 5, 11, 16, or 24 mM glucose (n = 9). Dead cells were collected from the growth media. Attached cells were washed and trypsinized. The fractions containing dead and attached cells were combined. Viability was measured with Via1-cassettes (Chemometec, Denmark) using the Nucleocounter NC-3000 platform. Data are presented as mean percentage of viable cells ± SEM, and groups were compared using the Mann-Whitney U-test (GraphPad Prism 5; level of significance, p < 0.05).

### Apoptosis and necrosis assay

Transfected cells (*Zip14* siRNA/control siRNA) were stimulated for 24 h in RPMI 1640 medium containing 5, 11, 16, or 24 mM glucose (n = 6 for each group, except n = 4 for the 16 mM Control siRNA group). Apoptosis was identified by the measurement of intracellular histone-associated DNA fragments and necrosis by the measurement of extracellular histone-associated DNA fragments using a photometric enzyme immunoassay (Cell Death Detection ELISAPLUS, Roche Applied Science, Germany) following the manufacturer’s instructions^5^. The immunoassay included both a negative as well as a positive control. Data are presented as mean optical density (OD) ± SEM. The Mann-Whitney U-test was used to compare *Zip14* siRNA-treated cells with control cells (GraphPad Prism 5; level of significance, p < 0.05).

## Results

### Effect of high glucose concentrations on *Zip14* mRNA expression

Using PrimeFlow-based flow cytometry, we measured the effect of glucose concentration on *Zip14*, *Znt8*, and *insulin (Ins)* mRNA expression, to determine whether *Zip14* is regulated by glucose and whether its expression is co-regulated with *insulin* mRNA levels. *Znt8* was used as a positive control for glucose dependence, as we have previously found decreased expression levels upon high glucose stimulation^3,45^.

**Figure 1:**
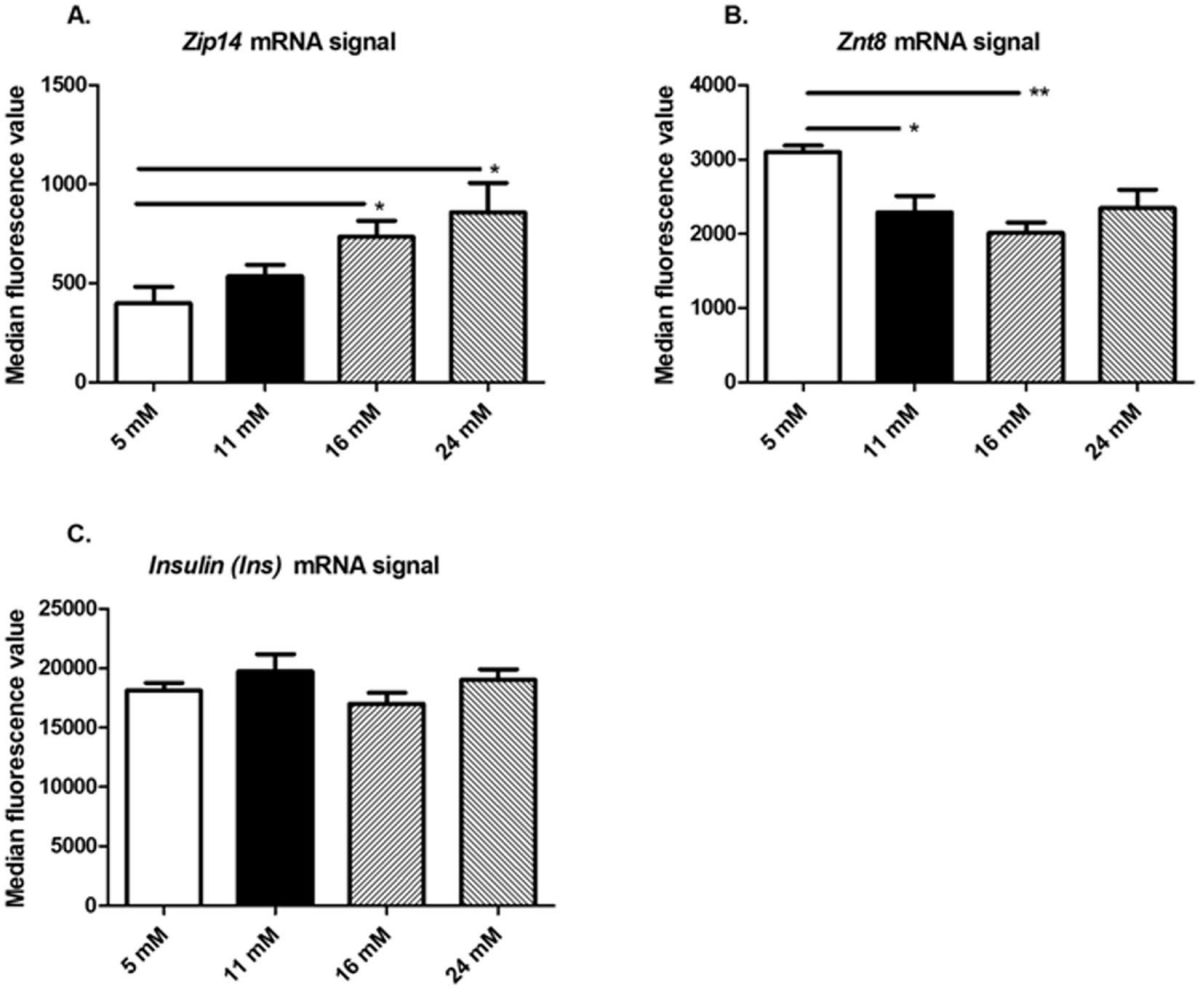
Measurements of *Zip14*, *Znt8*, and *insulin* mRNA by PrimeFlow RNA Assay. INS-1E cells were incubated with 5, 11, 16, or 24 mM glucose for 24 h. *Zip14*, *Znt8*, and *insulin (Ins)* mRNA levels were measured by flow cytometry using specific RNA probes incorporated in PrimeFlow RNA assays. (**A**) *Zip14* mRNA probe, (**B**) *Znt8* mRNA probe, and (**C**) *Insulin* mRNA probe. (**A**–**C**) Mean median fluorescence value ± SEM of indicated mRNA probe at 5, 11, 16, and 24 mM glucose. n = 4–6 in each group. *p < 0.05, **p < 0.01 compared with 5 mM glucose as reference.

A homogenous response of the cell population was observed in response to glucose stimulation, with a significant increase in *Zip14 m*RNA (p = 0.0123). Statistically significant increases in the *Zip14* mRNA signal were detected at 16 mM and 24 mM glucose vs. 5 mM (Fig. 1A). *Znt8* mRNA was also significantly regulated by glucose (p = 0.0071), with a homogenous response among the cells. Statistically significant decreases were detected at 11 mM and 16 mM glucose vs. 5 mM (Fig. 1B). There was no significant effect of glucose concentration on *insulin (Ins)* mRNA (p = 0.3177) (Fig. 1C).

In summary, there was an increase in *Zip14* mRNA upon stimulation with high glucose concentrations, while *Znt8* mRNA was decreased and *insulin (Ins)* mRNA was statistically unchanged. It should be noted, that a very high fluorescent signal was found for *insulin* (*Ins*) mRNA potentially masking small changes.

### Verification of Zip14 at RNA and protein levels in INS-1E cells

To investigate the effect of Zip14 within the beta-cell, Zip14 expression was silenced in INS-1E cells using *Zip14* siRNA. To verify the presence of Zip14 at both the mRNA and protein levels in INS-1E cells, as well as to evaluate the effects of siRNA treatment on Zip14 levels, Zip14 expression was measured in *Zip14* siRNA- and control siRNA-transfected cells using real-time PCR and targeted proteomics.

**Figure 2:**
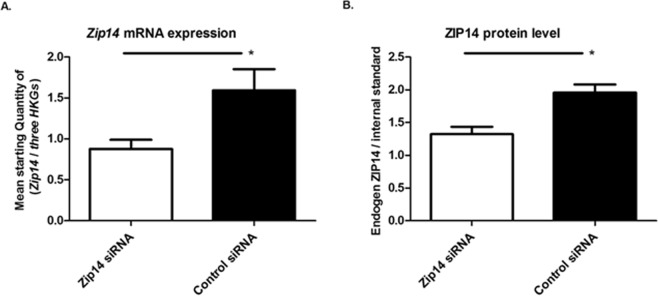
Transfection efficiency. Transfection efficiency was assessed by measurement of mRNA expression level by real-time PCR and at the protein level by selected reaction monitoring mass spectrometry. *Zip14* siRNA-treated cells were compared with non-targeting siRNA-treated cells (Control siRNA). (**A**) *Zip14* mRNA expression (n = 6). Results are expressed as the mean starting quantity of (*Zip14*/*three house-keeping genes*) ± SEM. (**B**) ZIP14 protein expressed as a mean ratio of an endogenous ZIP14-specific peptide/a heavy labeled standard peptide with the same sequence ± SEM. (Endogen Zip14/Internal standard) (n = 4–5). *p < 0.05.

A significant decrease in *Zip14* mRNA expression was observed in *Zip14* siRNA-treated cells. Zip14 mRNA was reduced by 45–65% in six independent experiments (by 45% in the example shown in Fig. 2A, p = 0.0152).

The protein level of Zip14 was analyzed by selected reaction monitoring MS. Zip14 protein expression was reduced by 32% (p = 0.0317) in *Zip14* siRNA- vs. control siRNA-transfected cells (Fig. 2B) (by 31% in a second analysis, p = 0.0635).

In summary, we confirmed the presence of Zip14 in the INS-1E beta-cell line at both the mRNA and protein levels, and demonstrated a siRNA transfection efficiency of 45–65% at the mRNA level resulting in approximately 30% down-regulation at the protein level.

### Effect of Zip14 silencing on the expression of selected zinc transporters and metallothioneins

The co-regulation of other zinc transporters that have previously been implicated in beta-cell function, and the zinc-sensing metallothioneins, Mt1a and Mt3 was investigated, to determine their regulation and whether expression of other zinc transporters might compensate for the down-regulation of Zip14. Znt1 was chosen as the widely expressed and main zinc efflux transporter at the plasma membrane, in order to illuminate if a change in Zip14-mediated zinc influx would affect the efflux^46^. Znt8, Znt3 and Znt5 are all localized at the insulin granules and in particular Znt8 and Znt3 have been shown to significantly regulate the glucose-stimulated insulin secretion^5,47–49^. Of the Zip family, Zip6 and Zip9 were investigated as hormone-sensitive transporters that, like Zip14, play a role in cell proliferation and apoptosis, while Zip13 was chosen as Zip13- and Zip14 KO-mice display many similarities in terms of growth retardation and impaired glucose metabolism^50–52^.

**Figure 3:**
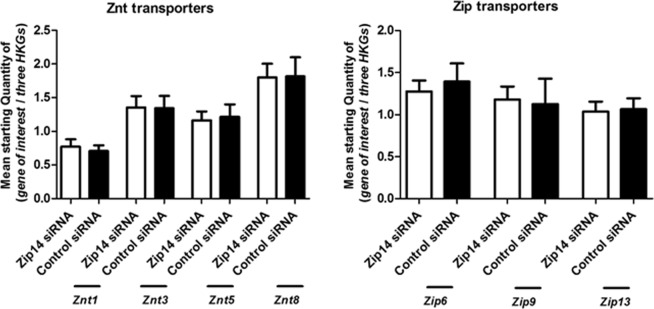
mRNA expression levels of zinc transporters. Measurements of zinc transporter mRNAs by real-time PCR in *Zip14* siRNA- and non-targeting siRNA-treated samples (Control siRNA). Results are expressed as the mean starting quantity of (*gene of interest*/*three house-keeping genes*) ± SEM. (**A**) ZnT transporters; *Znt1*, *Znt3*, *Znt5*, and *Znt8*. (**B**) Zip transporters; *Zip6*, *Zip9*, and *Zip13*.

**Figure 4:**
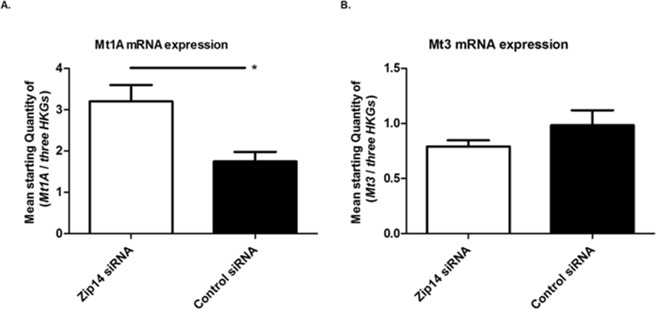
mRNA expression levels of metallothioneins. Measurements of metallothionein mRNA expression by real-time PCR in *Zip14* siRNA- and non-targeting siRNA-treated (Control siRNA) cells. Results are expressed as the mean starting quantity of (*gene of interest*/*three house-keeping genes*) ± SEM. (**A**) *Mt1A* mRNA expression. (**B**) *Mt3* mRNA expression. *p < 0.05.

None of the zinc transporters investigated (*Znt1*, *Znt3*, *Znt5*, *Znt8*, *Zip6*, *Zip9*, and *Zip13*) showed any mRNA response to Zip14 silencing (p = 0.4848, p = 0.69961, p = 0.9372, p = 0.8182, p = 0.9372, p = 0.8182, and p = 0.6991, respectively) (Fig. 3). A significant increase in *Mt1a* expression was observed after *Zip14* silencing (p = 0.0152), but no difference was detected in *Mt3* mRNA expression (p = 0.2403) (Fig. 4A,B).

In summary, no transcriptional compensation was observed by the other zinc transporters investigated, including *Znt1*, *Znt3*, and *Znt8*, in response to Zip14 silencing. However, increased expression of the metallothionein *Mt1a mRNA* was observed.

### Effect of Zip14 silencing on expression of other cellular proteins

We next investigated whether the expression of other proteins was regulated by Zip14 silencing using a large-scale proteomic approach.

In total, 3431 proteins were identified (a full list of these can be found in Supplementary Material 4). Of these, 52 were found to be differentially regulated (FDR < 0.05) in *Zip14* siRNA- vs. control siRNA-treated cells (listed in Tables 1–3). From their described properties (Uniprot.org), 11 proteins were identified as metal-binding (downregulated: Pdp2, Ranbp2, Resp2, Ampd2, Zfp512, Aco1; upregulated: Edem3, Chga, Srrm2, Adpgk, and Rnf213), two with a regulatory effect on insulin secretion (down-regulated, 6-phosphofructo-2-kinase/fructose-2, 6-biphosphatase 2 (Pfkfb2); and up-regulated, Chromogranin A (Chga)), as well as a down-regulation of the proliferation marker protein (Mki67) was found.

**Table 1:** List of proteins that were regulated by Zip14 silencing.

	siRNA ZIP14 vs. Control siRNA
Down-regulated	28 proteins
Up-regulated	24 proteins
Total	52 proteins

Numbers of proteins that were differentially expressed in Zip14 siRNA- and non-targeting siRNA-treated (Control siRNA) cells. Proteins with a fold change >1.136 or <0.88, and a false discovery rate <0.05, were included. 52 proteins were found in total, of which 28 were down-regulated and 24 were up-regulated.

**Table 2:** List of down-regulated proteins.

Down-regulated proteins			
Gene name	Accession Number	Protein description	Ratio (siRNAZIP14 vs. Control siRNA)
Dcp1a	D4AE80	mRNA-decapping enzyme 1A	0.70
Pdcd11	D3ZNI3	Protein RRP5 homolog	0.72
Pdp2	A0A0G2JSL7	[Pyruvate dehydrogenase [acetyl-transferring]]-phosphatase 2, mitochondrial	0.76
Cfdp1	A0A0G2K6H5	Craniofacial development protein 1	0.77
Mrps5	D3ZYT2	Mitochondrial ribosomal protein S5	0.78
Reps2	A0A0G2K1L4	RalBP1-associated Eps domain-containing protein 2	0.78
Zfp512	D3ZIF0	Zinc finger protein 512	0.79
Ap2a1	D3ZUY8	Adaptor protein complex AP-2, alpha 1 subunit	0.80
Mki67	D4A0Y6	Protein Mki67	0.80
Nup205	D4A7R3	Nucleoporin 205 kDa	0.81
Ranbp2	M0R3M4	E3 SUMO-protein ligase RanBP2	0.81
Pbdc1	G3V6C3	Protein Pbdc1	0.82
Noc2l	E9PTF3	Nucleolar complex protein 2 homolog	0.82
Nop56	Q4KLK7	Nucleolar protein 5 A	0.84
Wdr37	D3ZQ02	WD repeat-containing protein 37	0.84
Sept3	F1LMH0	Neuronal-specific septin-3	0.84
Ampd2	A0A0G2K3U1	AMP deaminase 2	0.85
Tmed4	B5DEM3	Transmembrane emp24 domain-containing protein 4	0.85
Numa1	F7FF45	Nuclear mitotic apparatus protein 1	0.85
Aco1	G3V6S2	Cytoplasmic aconitate hydratase	0.85
Slirp	D4A4W6	SRA stem-loop-interacting RNA-binding protein, mitochondrial	0.86
Pacs1	F1LPG3	Phosphofurin acidic cluster sorting protein 1	0.86
Strbp	D3ZDD7	Spermatid perinuclear RNA binding protein, isoform	0.87
Hnrnpr	Q566E4	Heterogeneous nuclear ribonucleoprotein R	0.87
Osbpl7	A0A0G2K0D5	Oxysterol-binding protein	0.87
Gga3	A0A0G2JV04	Protein Gga3	0.87
Pfkfb2	R9PXY6	6-phosphofructo-2-kinase/fructose-2, 6-biphosphatase 2, isoform CRA_a	0.87
Nop58	Q5PPK6	Nucleolar protein 58	0.88

List of the 28 proteins that were down-regulated in Zip14 siRNA- vs. non-targeting siRNA-treated (Control siRNA) cells. Proteins with a fold change >1.136 or <0.88, and a false discovery rate <0.05, were included. Gene name, accession number, and ratio are listed, together with a short description (from uni-prot.org, using the descriptions from the human database, when not available for Rattus norvegicus).

**Table 3:** List of up-regulated proteins.

Up-regulated proteins			
Gene name	Accession	Protein description	Ratio (siRNAZIP14 vs. Control siRNA)
Itln1	Q499T8	Intelectin 1 (Galactofuranose binding)	1.15
Rpl22l1	B2RZD5	60S ribosomal protein L22-like 1	1.15
Dera	F1M1H0	Deoxyribose-phosphate aldolase	1.16
Gba	B2RYC9	Glucosylceramidase	1.16
LOC681282	D3ZDG0	Unknown	1.16
Vgf	F1LP80	Neurosecretory protein VGF	1.16
Rnf213	F1M0R1	E3 ubiquitin-protein ligase RNF213	1.17
Rab3b	Q6P9W6	Ras-related protein Rab-3B	1.18
Adpgk	G3V784	ADP-dependent glucokinase, isoform CRA_a	1.18
Srrm2	A0A0G2K2M9	Serine/arginine repetitive matrix protein 2	1.18
Evi5	D3ZJN9	Ecotropic viral integration site 5 protein	1.18
Chga	F8QYX0	Chromogranin A	1.18
Rfc2	Q9QXI2	Replication factor C subunit 2 (Fragment)	1.18
Tspan6	B0BN20	Tetraspanin	1.19
Camk2b	F1LNI8	Calcium/calmodulin-dependent protein kinase type II subunit beta	1.20
Tgfbi	D4A8G5	Transforming growth factor-beta-induced protein ig-h3	1.22
Cdk12	A0A0G2K5U7	Cyclin-dependent kinase 12	1.23
RGD1560212*	A0JPQ5	N-alpha acetyltransferase 11 (88.6% homologue)	1.23
Edem3	D4AB70	alpha-1,2-Mannosidase	1.24
Wnk3	A0A0G2K2Y2	Serine/threonine-protein kinase WNK3	1.25
Cnn2	D3ZRX9	Calponin	1.26
Resp18	A0A0G2JZD6	Regulated endocrine-specific protein 18	1.37
Reep1	D4A193	Receptor expression-enhancing protein	1.39
Muc13	F1M9I3	Mucin-13	2.45

List of the 24 proteins that were up-regulated in Zip14 siRNA- vs. non-targeting siRNA-treated (Control siRNA) cells. Proteins with a fold change >1.136 or <0.88, and a false discovery rate <0.05, were included. Gene name, accession number, and ratio are listed, together with a short description (from uni-prot.org, using the descriptions from the human database, when not available for Rattus norvegicus). *Analyzed by the Blast function of uni-prot.org. RGD1560212 showed 88.6% homology with Mus musculus N-alpha acetyltransferase 11.

In summary, 52 proteins were found to be differentially regulated after Zip14 silencing, of which 11 were identified to be metal-binding, while others have known functions in insulin secretion and cellular proliferation.

**Figure 5:**
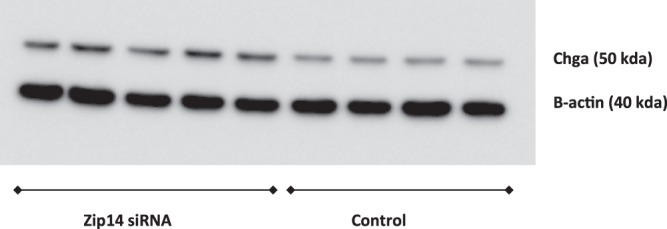
Western blotting of Chga (Chromogranin a) and B-actin (internal control). Sample 1–5 from left; Zip14 siRNA-treated samples. Sample 6–9 from left; non-targeted siRNA-treated samples (Control). 5 μg of protein were used.

As Chga (Chromogranin A) is a key regulatory pro-hormone, substantially affecting insulin secretion^53–55^ we chose to validate this protein by western blotting, using the same samples as in the proteomic experiments, comparing *Zip14* siRNA- and control siRNA-transfected cells. Western blotting confirmed the changes observed in Chga levels (Proteomics; ratio 1.18; p = 0.0007. Western blotting; ratio 1.76; p = 0.0635) (Fig. 5, as well as the full western blot is available in Supplementary Material 5).

### Ontological analysis of the large-scale proteomics data

Functional ontology analysis was used to characterize the list of differentially regulated proteins identified by large-scale proteomics following Zip14 silencing, to determine whether specific functional protein groups were selectively regulated.

121 proteins were included in the gene ontology analysis, which was undertaken using the bioinformatics software DAVID 6.8 Beta. Two annotation clusters were identified with an ES ≥ 1.3 (Table 4). When analyzing the complete list and the separate list of down-regulated proteins, an annotation cluster containing 11 ribosomal proteins was identified (ES = 1.7 and ES = 1.3, respectively). Of these, the majority were found to be mitochondrial (seven) and down-regulated (eight) (Table 4). Within the group of down-regulated proteins, an additional annotation cluster of mitochondrial proteins was identified (seven mitochondrial proteins, of which six were identified as mitochondrial transit proteins (ES = 1.6).

**Table 4:** Functional annotation clustering.

Functional Annotation Clustering				
Group cluster	Gene name	Accession	Regulation	Description
Ribosomal proteins (ES 1.7)*				
	Hnrnpr	Q566E4	Down	*Heterogeneous nuclear ribonucleoprotein R*
	Mrpl11	A0A0G2JYU2	Down	*39S ribosomal protein L11, mitochondrial*
	Mrpl28	D3ZJY1	Up	*39S ribosomal protein L28, mitochondrial*
	Mrpl44	Q4G067	Down	*39S ribosomal protein L44, mitochondrial*
	Mrps5	D3ZYT2	Down	*28S ribosomal protein S5, mitochondrial*
	Mrps16	D4A7X1	Down	*28S ribosomal protein S16, mitochondrial*
	Mrps30	D4A833	Down	*28S ribosomal protein S30, mitochondrial*
	Mrps34	D4ABM5	Down	*28S ribosomal protein S34, mitochondrial*
	Nop58	Q5PPK6	Down	*Nucleolar protein 58*
	Rpl22l1	B2RZD5	Up	*60S ribosomal protein L22-like 1*
	Rps5	B0BN81	Up	*40S ribosomal protein S5*
Mitochondrial transit peptides (ES 1.6)**				
	Ccbl1	G3V827	Down	*Cysteine conjugate-beta lyase 1, isoform CRA_a*
	Cox7a2	B2RYS0	Down	*Cytochrome c oxidase subunit 7A2, mitochondrial*
	Dlst	G3V6P2	Down	*Dihydrolipoyllysine-residue succinyltransferase component of 2-oxoglutarate dehydrogenase complex, mitochondrial*
	Micu1***	A0A0G2K0T5	Down	*Calcium uptake protein 1, mitochondrial*
	Mrpl11	A0A0G2JYU2	Down	*39S ribosomal protein L11, mitochondrial*
	Pdp2	A0A0G2JSL7	Down	*[Pyruvate dehydrogenase [acetyl-transferring]]-phosphatase 2, mitochondrial*
	Prdx3	G3V7I0	Down	*Thioredoxin-dependent peroxide reductase, mitochondrial*

Functional annotation clustering was performed using the bioinformatics software DAVID (https://david-d.ncifcrf.gov/). Proteins with ≥3 scans, ≥1 unique peptide, fold change >1.136 or <0.88, and a p-value < 0.05 were included and analyzed together, or separated into down-regulated and up-regulated proteins. Two annotation clusters were found with an ES ≥1.3. *ES of 1.7 when interrogating the complete list of proteins. This cluster was also found when interrogating only down-regulated proteins (ES 1.3). **ES of 1.6 when investigating only down-regulated proteins. ***MICU1 is not classified as a transit peptide, but was within the cluster group as a mitochondrial protein. Gene names are listed, together with accession number and a short description of each gene and cluster. The description of genes was accessed from uni-prot.org, using the descriptions from the human database, when not available for Rattus norvegicus. ES: enrichment score.

In summary, analysis of proteins that were differentially regulated following Zip14 silencing identified a group of mitochondrial proteins, especially ribosomal mitochondrial proteins, of which most were down-regulated.

### Identification of mitochondrial proteins and pathways

In order to analyze the group of mitochondrial proteins further, we used the Mitocarda 2.0 compendium^43^ and found that 365 out of the 3431 quantified proteins can be assigned to the mitochondria. The general level of the mitochondrial proteins was not altered when comparing *Zip14* siRNA- vs. non-targeting siRNA-treated (Control siRNA) cells (median ratio 0.98 of the 365 proteins; Supplementary Material 6) indicating a similar level of mitochondrial proteins in the two groups.

When looking at the functions of the identified mitochondrial proteins, a total of 25 proteins of mitochondrial ribosomes were detected and all but 2 had ratio values (Zip14 siRNA vs non-targeted siRNA treatment) below 1.0 (mean ratio of 0.89), supporting the findings of the gene ontology analysis (a total list of the 25 detected mitochondrial ribosomal proteins are found in Supplementary Material 7).

Of interest, proteins within the oxidative phosphorylation process seemed to be affected as well, with 33 out of 34 proteins with a ratio value (Zip14 siRNA vs non-targeted siRNA treatment) below 1.0 (median 0.89) (Supplementary Material 8).

Proteins involved in the degradation of branched chain amino acids, fatty acid degradation, and citric acid cycle were not generally affected by *Zip14* siRNA treatment, as the mean ratio of *Zip14* siRNA vs. non-targeting siRNA treated (Control siRNA) cells were comparable (mean ratio of 0.99, 1.00, and 0.97, respectively).

### Measurements of insulin secretion and storage in *Zip14* siRNA-treated cells

Next, we investigated the functional effects of *Zip14* silencing on insulin secretion and storage after 24 h of glucose stimulation. The experiment was designed in a dose-related manner, in order to functionally investigate the insulin secretion process, with glucose concentrations covering the response range originally found by Merglen *et al*. when describing the INS-1E cell line^35^, The cells were thereby exposed to a range of hypo- to hyperglycemic conditions compared to their normal standard environment of 11 mM glucose.

**Figure 6:**
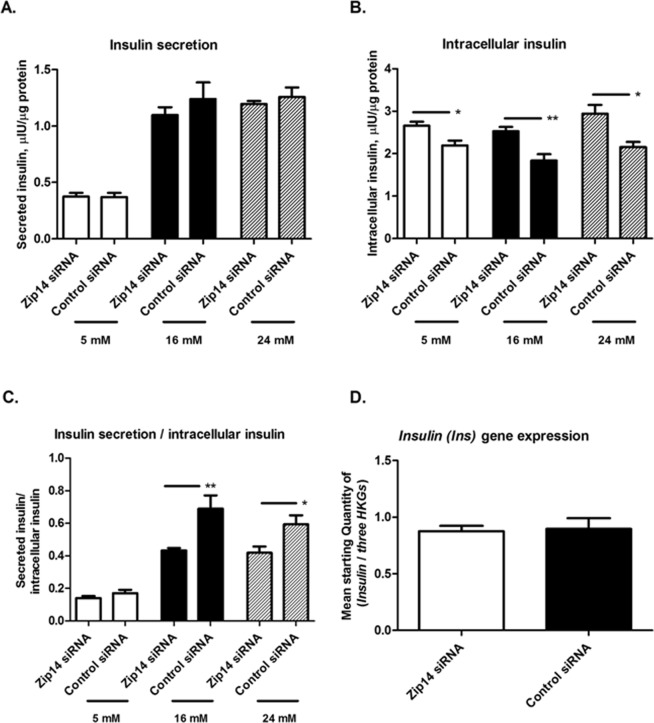
Insulin secretion and intracellular insulin content of INS-1E cells treated with *Zip14* siRNA or non-targeting siRNA at diverse glucose concentrations. Cells were treated with *Zip14* siRNA or non-targeting siRNA (Control siRNA) and incubated for 24 h in medium containing 5 mM, 16 mM, or 24 mM glucose (n = 6 replicates, except for 16 mM Control siRNA, where n = 5). Insulin measurements were performed using Rat Insulin Elisa kits and normalized to the protein concentration. Data are mean ± SEM. *p < 0.05, **p < 0.01. (**A**) Insulin secretion, expressed as µIU insulin/µg of protein. (**B**) Intracellular insulin content, expressed as µIU insulin/µg of protein. (**C**) Insulin secretion/intracellular insulin content ratio. (**D**) *Insulin (Ins)* mRNA expression at 11 mM glucose, expressed as mean starting quantity of (*insulin*/*thre*e *house-keeping genes*) ± SEM, measured by real-time PCR.

Insulin secretion in the presence of 5, 16, or 24 mM glucose was not different between *Zip14* siRNA- and control siRNA-treated INS-1E cells (p = 0.6991, p = 0.7922, and p = 0.6991, respectively) (Fig. 6A). Intracellular insulin content was, however, increased in *Zip14* siRNA-treated cells, reaching statistical significance in cells incubated in 5, 16, and 24 mM glucose (p = 0.0152, p = 0.0043, and p = 0.0260, respectively) (Fig. 6B). Reflecting this, the ratio between secreted and intracellular insulin was decreased in the presence of 16 and 24 mM glucose in the *Zip14* siRNA-treated cells (p = 0.0087 and p = 0.0260, respectively). At 5 mM glucose, the ratio showed the same pattern, although this effect was not statistically significant (p = 0.2403) (Fig. 6C). To investigate if Zip14 in itself has an effect on the Insulin mRNA, the expression level of *insulin (Ins)* was measured at the basic condition of 11 mM glucose. *Insulin (Ins)* was found to be statistically comparable between *Zip14* siRNA- and control siRNA-treated cells (p = 0.8182) (Fig. 6D).

In summary, increased intracellular insulin content, in combination with unaltered *insulin (Ins)* gene expression and secretion of insulin, was observed following Zip14 silencing.

### Effect of Zip14 silencing on cell viability, apoptosis, and necrosis

To functionally investigate whether Zip14 influences cell survival, cell viability, apoptosis, and necrosis were quantified in *Zip14* siRNA- and control siRNA-treated cells at diverse glucose concentrations, alongside measurement of the *Bax*/*Bcl-2* mRNA expression index as an indicator of activation of the intrinsic apoptotic pathway.

**Figure 7:**
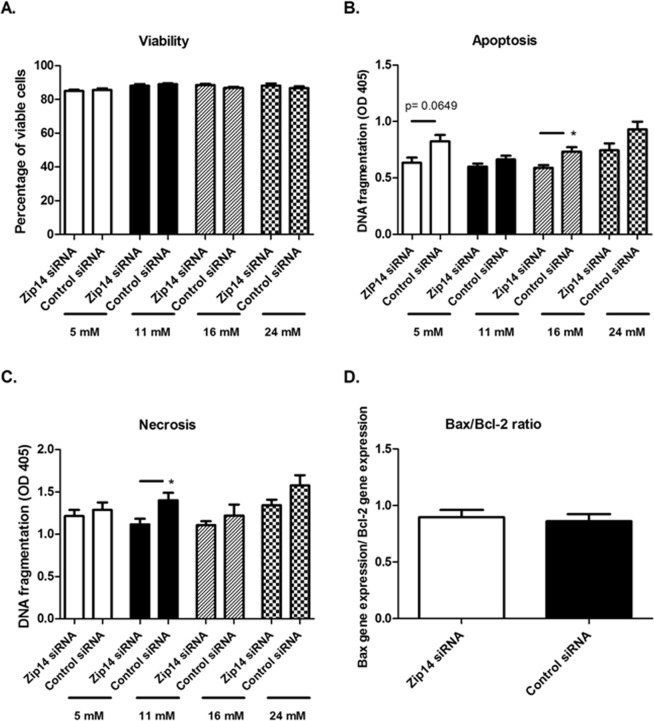
Overall viability, apoptosis, necrosis, and *Bax*/*Bcl-2* index in INS-1E cells treated with *Zip14* siRNA or non-targeting siRNA after 24 h incubation in diverse glucose concentrations. INS-1E cells were transfected with *Zip1*4 siRNA or non-targeting siRNA (Control siRNA) and incubated for 24 h in medium containing 5 mM, 11 mM, 16 mM, or 24 mM glucose. Viability measurements were performed using Via1-cassettes (Chemometec, Denmark) in a Nucleocounter NC-3000 (experiment performed twice, n = 9 in total). A Cell Death Detection ELISAPLUS kit was used for the measurement of apoptosis or necrosis. Detection was performed using an ELISA Reader at 405 nm. n = 6 in each group, except for the 16 mM Control siRNA group, where n = 4. Data are mean ± SEM. (**A**) Percentage of viable cells, assessed using acridine orange and DAPI staining. (**B**) Apoptosis, measured as the amount of intracellular histone-associated DNA fragments. (**C**) Necrosis, measured as the amount of histone-associated DNA fragments in the culture medium. *p < 0.05. (**D**) The *Bax/Bcl-*2 index, measured by real-time PCR. Gene expression was measured as mean starting quantity (*gene of interest*/*three house-keeping genes*) ± SEM.

The general viability of both *Zip14* siRNA-treated and control siRNA-treated cells was high, with a mean viability between 84% and 89%. The percentage of viable cells, showed no difference when comparing *Zip14* siRNA treated cells vs. siRNA controls (5 mM glucose, p = 0.7572; 11 mM, p = 0.4797; 16 mM, p = 0.0932; 24 mM, p = 0.4013) (Fig. 7A).

Despite comparable overall viability, active apoptosis, assessed by quantifying intracellular histone-associated DNA fragments, was decreased in *Zip14* siRNA-treated INS-1E cells. This result was statistically significant at 16 mM glucose and close to significance at 5 mM glucose (p = 0.0190 and p = 0.0649, respectively). The same tendency was found after incubation in 11 and 24 mM glucose, although these differences were not statistically significant (p = 0.2403 and p = 0.1797, respectively) (Fig. 7B).

Necrotic beta-cell death, judged by quantifying histone-associated DNA fragments in the culture supernatant, showed the same pattern as for apoptosis, with a decrease in *Zip14* siRNA- vs. control siRNA-treated cells. The difference was significant at 11 mM (p = 0.0411), but not at 5, 16, or 24 mM (p = 0.6991, p = 0.7619, and p = 0.1320, respectively) (Fig. 7C).

In addition, the *bax*/*bcl2* ratio, an indicator of susceptibility to apoptosis, was measured by real-time PCR at 11 mM glucose. However, there was no effect of Zip14 silencing on this parameter (Fig. 7D).

In summary, signs of decreased apoptosis and necrosis were found in Zip14-silenced cells, but there was no effect on the general viability of the cells or the *Bax*/*Bcl-2* index.

## Discussion

Studies of Zip14 knockout mice suggest a role for Zip14 in glucose homeostasis and beta-cell function, because Zip14 knockout animals display high circulating insulin levels, alongside signs of an insulin secretory deficit during high glucose stimulation^26,31^. Further, the pancreatic expression level of Zip14 is low in type 2 diabetic individuals^22^. In this study, we confirmed the presence of Zip14 in INS-1E cells and showed effects of glucose concentration on its mRNA expression. Using targeted proteomics, we identified the Zip14 protein within the 60–100 kDa range, which was comparable to findings of Liuzzi and co-authors, who reported a band size for Zip14 of 70 kDa by western blotting in AML12 hepatocytes. This apparent molecular mass is hypothesized to be due to glycosylation, given that Zip14 has a predicted size of 54 kDa, which is also observed in other cell-types^56^. The functional studies supported a role for Zip14 in insulin processing and also potentially in mitochondrial function, because the expression of a group of ribosomal mitochondrial proteins as well as the general level of mitochondrial proteins involved in oxidative phosphorylation was affected by *Zip14* silencing in our large-scale proteomic analysis. We were able to perform the sub-analyses of mitochondrial proteins due to the high amount of mitochondrial proteins measured by large scale proteomics.

Mitochondrial function is essential for insulin secretion, because ATP is the main signal that initiates insulin secretion. Upon glucose stimulation, ATP is generated within the mitochondria through oxidative phosphorylation^57^. Mitochondrial DNA encodes proteins that are essential for oxidative phosphorylation, and the mRNAs corresponding to these proteins are translated by mitochondrial ribosomes, which are encoded by nuclear DNA^58^. Several of these proteins were found to be significantly down-regulated at the protein level in this study. The potential importance of these proteins is underlined by the fact that, in rho0 MIN6 and INS-E cells, which lack the mitochondrial genome, glucose-stimulated insulin secretion is absent^59,60^. Of interest, one of the main proteins, Slirp, required for efficient translocation of mRNA to the mitochondrial ribosome, was found to be significantly down-regulated in *Zip14* siRNA treated samples vs. control (Ratio 0.86, listed in Table 2)^61^.

Examining the 34 mitochondrial proteins directly involved in oxidative phosphorylation, the mean ratio of *Zip14* siRNA/non-targeted siRNA was found to be 0.89 indicating a general lower level of proteins involved in this process in a Zip14 deficient state. Relevant, the oxidative phosphorylation protein Cox7a2, was found to be significantly down-regulated (Table 4). Cox7a2 is an important assembly factor in the respiratory chain, modulating the interaction between complexes III (CIII) and IV (CIV) thereby optimizing cellular metabolism^62^.

Zinc ions regulate genes at a transcriptional level, through the transcription factor, metal-regulatory transcription factor 1, and it may be that silencing of Zip14 thereby affects the production of other proteins with a mitochondrial function. The observed low expression levels of proteins involved in the mitochondrial protein translation are supported by our findings of a general lower level of mitochondrial oxidative phosphorylation proteins. The dysregulated insulin secretion, as seen in our functional insulin secretion studies, might be a consequence of compromised mitochondrial metabolism. In the pathology of diabetes, mutations in mitochondrial DNA, as well as altered translation of these genes into proteins, are believed to play a role^57,58^. Around 1% of all cases of diabetes mellitus are thought to arise from mutations within mitochondrial DNA^57^.

In our functional studies of the insulin secretion process, a decreased ratio of secreted insulin to intracellular insulin content was observed upon a Zip14 down-regulation, further reflected in an increased intracellular insulin content at all measured glucose concentrations (Fig. 6.). Because *insulin (Ins)* expression was not affected by *Zip14* silencing in cells cultured in 11 mM glucose, the accumulation of intracellular insulin likely reflect an abnormal secretion process induced by the partial deficiency of Zip14.

In line with the dysregulated insulin secretion observed in Zip14 siRNA treated cells, the expression of proteins involved in insulin secretion was also affected by Zip14 silencing. Of special interest was the up-regulation of chromogranin A (Chga), which was further confirmed by western blotting. Chga is a well-known secretory granule protein that has several peptide derivatives, including betagranin and pancreastatin, which strongly inhibit glucose-stimulated insulin release^54,55^. In line with this, Chga knock-out mice exhibit an increased glucose-stimulated insulin secretion and recent research further suggest that Chga is linked to the regulation of mitochondrial volume, as knock-down of Chga in beta-cells results in increased mitochondrial volume and expression of mitochondrial proteins^53^.

In addition, 6-phosphofructo-2-kinase/fructose-2, 6-biphosphatase 2 (Pfkfb2) expression was down-regulated. Studies of INS-1E cells have shown a stimulatory effect of Pfkfb2 on insulin secretion, which was hypothesized to be linked to its glucokinase activity^63^. Further, the continuous and stable release of insulin in response to glucose is dependent on mitochondrial Ca^2+^ import by the Ca^2+^ uniporter (MCU)^64^. Interestingly, our studies showed a significant down-regulation of the mitochondrial calcium uptake protein 1 (Micu1), which is a known subunit and regulator of the MCU transporter^57^. Furthermore, functional studies in HELA cells have shown that Micu1 is required for mitochondrial Ca^2+^ uptake by the Ca^2+^ uniporter^65^.

Despite the possibility that mitochondrial dysfunction might lead to increased levels of oxidative stress and subsequently cell death^66^, Zip14 silencing did not inhibit cell viability. Instead lowered apoptosis and necrosis were indicated by measurement of histone-associated DNA fragments in Zip14 silenced cells and the surrounding medium, respectively. However, no differences were detected in *Bax*/*Bcl-2* expression, which are regulators of the intrinsic apoptotic pathway, dependent on mitochondrial cytochrome c release^66^. The extent to which Zip14 expression influences apoptosis and necrosis remains unclear, because cell viability was high and unaffected by a 30% down-regulation of the Zip14 protein. Given that some degree of apoptosis inevitably occurs following rapid cell proliferation, our findings might reflect a decreased proliferative rate in the Zip14 siRNA-treated cells. Zip14 plays a general role in proliferation and differentiation, indicated by the decreased proliferation and differentiation of chondrocytes, hepatocytes, and adipocytes in Zip14 knockout mice^24,27,32^. Consistent with this, the proliferation marker protein Ki-67 (Mki67) was significantly down-regulated following Zip14 siRNA treatment. Mki67 is a reliable marker of proliferation, because it is highly expressed in all phases of proliferation, but absent in the resting state^67^.

The observed functional effects of Zip14 silencing likely reflect changes in intracellular zinc homeostasis, because Zip14 functions as a zinc importing protein. The zinc transporter family consists of no less than 10 Znts and 14 Zips^68^. However, the complexity and interplay of these zinc transporters are far from understood. Znt1 is known as a main and generally expressed zinc-exporting protein at the plasma-membrane, however no effect was found on Znt1 expression upon a Zip14 down-regulation indicating no compensatory effect by this general zinc-efflux transporter^46^. As Znt 3, 5, and 8 are localized at the insulin granules and thought to play an important functional role in regulating the glucose-stimulated insulin secretion, one could have suspected an effect of a Zip14 down-regulation as insulin secretion was affected in our experiment^5,47–49^. In this study, no such effect was found, indicating that their role is not directly dependent on Zip14 function. Regarding the Zip family, we choose to investigate Zip6 and Zip9, both hormone-sensitive transporters, that like Zip14 seems to have a function in apoptosis and proliferation in various cell types, however no indications of a regulatory interplay was found upon a Zip14 regulation^50,51^. Additionally, Zip13 was measured, as an interesting transporter in terms of Zip14, as Zip13 and Zip14 knock-out mice share many similarities i.e. impaired glucose homeostasis and growth retardation and could therefore be a potential target for a compensatory mechanism^52^. Overall, the change in Zip14 expression was not compensated for by any of the other zinc transporters measured (Znt 1, 3, 5, 8 and Zip 6, 9, 13), pointing at a functional change in intracellular zinc homeostasis upon a Zip14 regulation. Albeit, it cannot be excluded that the down-regulation of Zip14 was compensated by other zinc transporters beyond the scope of our investigations.

A decreased amount of cytosolic zinc following a Zip14 down-regulation has been indicated by a reduced Mt1 expression in a study by Troche and co-authors, investigating Zip14 siRNA-treated adipocytes^24^. In our experiments, we detected an up-regulation of *Mt1a* expression following Zip14 silencing. This might reflect increased levels of oxidative stress resulting from mitochondrial dysfunction, with Mt1a being up-regulated to scavenge free radicals and thereby prevent cell death^12^.However, in support of a change in intracellular metals upon Zip14 silencing, the expression of several metal-binding proteins was altered, including ACO1, which is an iron-sensing protein^69^. Notably, Zip14 plays an important role as an iron transporting protein in liver^29,70^, and it cannot be excluded that dysregulation of iron homeostasis contributes to the cellular phenotype that we have described.

The INS-1E cells are were cultivated at 11 mM glucose as originally described by Merglen *et al*. and Asfari *et al*.^35.36^. The glucose-stimulation experiments were designed in a dose-related manner, using diverse glucose concentrations ranging from low (5 mM) to high glucose (24 mM), in order to functionally investigate the glucose-stimulated response of the INS-1E cells focusing on the effect of a Zip14 siRNA down-regulation. One cannot exclude, that the cells were somehow glucose deprived at 5 mM, with the standard growth condition being 11 mM. However, the viability of the cells at 5 mM were comparable with the other glucose concentrations (11,16 or 24 mM), just as the pattern of apoptosis and necrosis were similar (Fig. 7) supporting that measurements of insulin secretion under such conditions are still feasible and reflect the regulatory function of glucose on INS-1E cells.

In conclusion, our results establish a role of Zip14 within the beta-cell and imply that a down-regulation of Zip14 impairs insulin processing and potentially affects mitochondrial function. As this is the first study into the function of Zip14 in the pancreatic beta-cell, future studies should aim at further exploring these novel links. Since signs of low pancreatic expression levels of Zip14 have been demonstrated in T2D, enhanced Zip14 activity could potentially serve as a future pharmacological target in the treatment of beta-cell dysfunction.

## Supplementary information

Supplementary material

## References

[1] Alejandro EU, Gregg B, Blandino-Rosano M, Cras-Meneur C, Bernal-Mizrachi E (2015). Natural history of beta-cell adaptation and failure in type 2 diabetes. Mol. Aspects Med..

[2] Chimienti, F., Rutter, G. A., Wheeler, M. B. & Wijesekara, N. Zinc and diabetes in *Zinc in Human Health* (ed. Rink, L.) 493–513 (IOS Press, 2011).

[3] Smidt K (2009). SLC30A3 responds to glucose- and zinc variations in beta-cells and is critical for insulin production and *in vivo* glucose-metabolism during beta-cell stress. PLoS. One..

[4] Huang L, Yan M, Kirschke CP (2010). Over-expression of ZnT7 increases insulin synthesis and secretion in pancreatic beta-cells by promoting insulin gene transcription. Exp. Cell Res..

[5] Petersen AB (2011). siRNA-mediated knock-down of ZnT3 and ZnT8 affects production and secretion of insulin and apoptosis in INS-1E cells. APMIS.

[6] Chimienti F, Devergnas S, Favier A, Seve M (2004). Identification and cloning of a beta-cell-specific zinc transporter, ZnT-8, localized into insulin secretory granules. Diabetes.

[7] Wijesekara N (2010). Beta cell-specific Znt8 deletion in mice causes marked defects in insulin processing, crystallisation and secretion. Diabetologia.

[8] Sladek R (2007). A genome-wide association study identifies novel risk loci for type 2 diabetes. Nature.

[9] Kambe T (2002). Cloning and characterization of a novel mammalian zinc transporter, zinc transporter 5, abundantly expressed in pancreatic beta cells. J Biol. Chem..

[10] Aguilar MV (2007). Plasma mineral content in type-2 diabetic patients and their association with the metabolic syndrome. Ann. Nutr. Metab.

[11] Al-Maroof RA, Al-Sharbatti SS (2006). Serum zinc levels in diabetic patients and effect of zinc supplementation on glycemic control of type 2 diabetics. Saudi. Med. J..

[12] Maret, W. Human Zinc Biochemistry in *Zinc in Human Health* (ed. Rink, L.) 45–62 (IOS Press, 2011).

[13] Maret W (2011). Metals on the move: zinc ions in cellular regulation and in the coordination dynamics of zinc proteins. Biometals.

[14] Beyersmann D, Haase H (2001). Functions of zinc in signaling, proliferation and differentiation of mammalian cells. Biometals.

[15] Truong-Tran AQ, Carter J, Ruffin RE, Zalewski PD (2001). The role of zinc in caspase activation and apoptotic cell death. Biometals.

[16] Haase H, Maret W (2005). Fluctuations of cellular, available zinc modulate insulin signaling via inhibition of protein tyrosine phosphatases. J. Trace Elem. Med. Biol..

[17] Haase, H. & Rink, L. Zinc Signaling in *Zinc in Human Health* (ed. Rink, L.) 94–117 (IOS Press, 2011).

[18] McCord MC, Aizenman E (2014). The role of intracellular zinc release in aging, oxidative stress, and Alzheimer’s disease. Front Aging Neurosci..

[19] Dineley KE, Richards LL, Votyakova TV, Reynolds IJ (2005). Zinc causes loss of membrane potential and elevates reactive oxygen species in rat brain mitochondria. Mitochondrion..

[20] Jitrapakdee S, Wutthisathapornchai A, Wallace JC, MacDonald MJ (2010). Regulation of insulin secretion: role of mitochondrial signalling. Diabetologia.

[21] Kimura T, Kambe T (2016). The Functions of Metallothionein and ZIP and ZnT Transporters: An Overview and Perspective. Int. J Mol. Sci..

[22] Liu Y (2015). Characterization of Zinc Influx Transporters (ZIPs) in Pancreatic beta Cells: roles in regulating cytosolic zinc homeostasis and insulin secretion. J Biol. Chem..

[23] Hardy AB (2015). Zip4 mediated zinc influx stimulates insulin secretion in pancreatic beta cells. PLoS One.

[24] Troche C, Aydemir TB, Cousins RJ (2015). Zinc transporter Slc39a14 regulates inflammatory signaling associated with hypertrophic adiposity. Am. J. Physiol Endocrinol. Metab..

[25] Maxel T (2015). Gene expression of the zinc transporter ZIP14 (SLC39a14) is affected by weight loss and metabolic status and associates with PPARgamma in human adipose tissue and 3T3-L1 pre-adipocytes. BMC. Obes..

[26] Beker AT (2012). Zinc transporter ZIP14 functions in hepatic zinc, iron and glucose homeostasis during the innate immune response (endotoxemia). PLoS. One..

[27] Hojyo S (2011). The zinc transporter SLC39A14/ZIP14 controls G-protein coupled receptor-mediated signaling required for systemic growth. PLoS. One..

[28] Pinilla-Tenas JJ (2011). Zip14 is a complex broad-scope metal-ion transporter whose functional properties support roles in the cellular uptake of zinc and nontransferrin-bound iron. Am. J. Physiol Cell Physiol.

[29] Nam H (2013). ZIP14 and DMT1 in the liver, pancreas, and heart are differentially regulated by iron deficiency and overload: implications for tissue iron uptake in iron-related disorders. Haematologica.

[30] Jenkitkasemwong S (2015). SLC39A14 Is Required for the Development of Hepatocellular Iron Overload in Murine Models of Hereditary Hemochromatosis. Cell Metab.

[31] Aydemir TB, Troche C, Kim MH, Cousins RJ (2016). Hepatic ZIP14-mediated Zinc Transport Contributes to Endosomal Insulin Receptor Trafficking and Glucose Metabolism. J Biol. Chem..

[32] Aydemir TB, Sitren HS, Cousins RJ (2012). The zinc transporter Zip14 influences c-Met phosphorylation and hepatocyte proliferation during liver regeneration in mice. Gastroenterology.

[33] Taylor KM, Morgan HE, Johnson A, Nicholson RI (2005). Structure-function analysis of a novel member of the LIV-1 subfamily of zinc transporters, ZIP14. FEBS Lett..

[34] Mohanasundaram D (2011). Ultrastructural analysis, zinc transporters, glucose transporters and hormones expression in New world primate (Callithrix jacchus) and human pancreatic islets. Gen. Comp Endocrinol..

[35] Merglen A (2004). Glucose sensitivity and metabolism-secretion coupling studied during two-year continuous culture in INS-1E insulinoma cells. Endocrinology.

[36] Asfari M (1992). Establishment of 2-mercaptoethanol-dependent differentiated insulin-secreting cell lines. Endocrinology.

[37] Vandesompele J (2002). Accurate normalization of real-time quantitative RT-PCR data by geometric averaging of multiple internal control genes. Genome Biol..

[38] Birkler RI, Nochi Z, Gregersen N, Palmfeldt J (2016). Selected reaction monitoring mass spectrometry for relative quantification of proteins involved in cellular life and death processes. J Chromatogr. B Analyt. Technol. Biomed. Life Sci..

[39] MacLean B (2010). Skyline: an open source document editor for creating and analyzing targeted proteomics experiments. Bioinformatics..

[40] Baiges I, Palmfeldt J, Blade C, Gregersen N, Arola L (2010). Lipogenesis is decreased by grape seed proanthocyanidins according to liver proteomics of rats fed a high fat diet. Mol. Cell Proteomics..

[41] Benjamini Y, Drai D, Elmer G, Kafkafi N, Golani I (2001). Controlling the false discovery rate in behavior genetics research. Behav. Brain Res..

[42] Huang DW, Sherman BT, Lempicki RA (2009). Systematic and integrative analysis of large gene lists using DAVID bioinformatics resources. Nat. Protoc..

[43] Calvo SE, Clauser KR, Mootha VK (2016). MitoCarta2.0: an updated inventory of mammalian mitochondrial proteins. Nucleic Acids Res..

[44] Kanehisa M, Furumichi M, Tanabe M, Sato Y, Morishima K (2017). KEGG: new perspectives on genomes, pathways, diseases and drugs. Nucleic Acids Res..

[45] Nygaard SB (2015). Exogenous metallothionein potentiates the insulin response at normal glucose concentrations in INS-1E beta-cells without disturbing intracellular ZnT8 expression. Basic Clin. Pharmacol. Toxicol..

[46] Kambe T, Yamaguchi-Iwai Y, Sasaki R, Nagao M (2004). Overview of mammalian zinc transporters. Cell Mol. Life Sci..

[47] Kambe T (2002). Cloning and characterization of a novel mammalian zinc transporter, zinc transporter 5, abundantly expressed in pancreatic beta cells. J Biol. Chem..

[48] Chimienti F (2006). *In vivo* expression and functional characterization of the zinc transporter ZnT8 in glucose-induced insulin secretion. J. Cell Sci..

[49] Smidt K (2016). The zinc transporter ZNT3 co-localizes with insulin in INS-1E pancreatic beta cells and influences cell survival, insulin secretion capacity, and ZNT8 expression. Biometals.

[50] Matsui C (2017). Zinc and its transporter ZIP6 are key mediators of breast cancer cell survival under high glucose conditions. FEBS Lett..

[51] Thomas P, Pang Y, Dong J, Berg AH (2014). Identification and characterization of membrane androgen receptors in the ZIP9 zinc transporter subfamily: II. Role of human ZIP9 in testosterone-induced prostate and breast cancer cell apoptosis. Endocrinology.

[52] Fukada T, Hojyo S, Furuichi T (2013). Zinc signal: a new player in osteobiology. J Bone Miner. Metab.

[53] Wollam J (2017). Chromogranin A regulates vesicle storage and mitochondrial dynamics to influence insulin secretion. Cell Tissue Res..

[54] Tatemoto K (1986). Pancreastatin, a novel pancreatic peptide that inhibits insulin secretion. Nature.

[55] Schmid GM (2007). Inhibition of insulin secretion by betagranin, an N-terminal chromogranin A fragment. J Biol. Chem..

[56] Liuzzi JP, Aydemir F, Nam H, Knutson MD, Cousins RJ (2006). Zip14 (Slc39a14) mediates non-transferrin-bound iron uptake into cells. Proc. Natl. Acad. Sci. USA.

[57] Wiederkehr A, Wollheim CB (2012). Mitochondrial signals drive insulin secretion in the pancreatic beta-cell. Mol. Cell Endocrinol..

[58] Sylvester JE, Fischel-Ghodsian N, Mougey EB, O’Brien TW (2004). Mitochondrial ribosomal proteins: candidate genes for mitochondrial disease. Genet. Med..

[59] Soejima A (1996). Mitochondrial DNA is required for regulation of glucose-stimulated insulin secretion in a mouse pancreatic beta cell line, MIN6. J Biol. Chem..

[60] Kennedy ED, Maechler P, Wollheim CB (1998). Effects of depletion of mitochondrial DNA in metabolism secretion coupling in INS-1 cells. Diabetes.

[61] Lagouge M (2015). SLIRP Regulates the Rate of Mitochondrial Protein Synthesis and Protects LRPPRC from Degradation. PLoS Genet..

[62] Cogliati S (2016). Mechanism of super-assembly of respiratory complexes III and IV. Nature.

[63] Arden C (2008). A role for PFK-2/FBPase-2, as distinct from fructose 2,6-bisphosphate, in regulation of insulin secretion in pancreatic beta-cells. Biochem. J.

[64] Quan X (2015). Essential role of mitochondrial Ca2 +uniporter in the generation of mitochondrial pH gradient and metabolism-secretion coupling in insulin-releasing cells. J Biol. Chem..

[65] Perocchi F (2010). MICU1 encodes a mitochondrial EF hand protein required for Ca(2+) uptake. Nature.

[66] Vander Heiden MG, Thompson CB (1999). Bcl-2 proteins: regulators of apoptosis or of mitochondrial homeostasis?. Nat. Cell Biol..

[67] Scholzen T, Gerdes J (2000). The Ki-67 protein: from the known and the unknown. J Cell Physiol.

[68] Liuzzi JP, Cousins RJ (2004). Mammalian zinc transporters. Annu. Rev. Nutr..

[69] Anderson CP, Shen M, Eisenstein RS, Leibold EA (2012). Mammalian iron metabolism and its control by iron regulatory proteins. Biochim. Biophys. Acta.

[70] Zhao N, Gao J, Enns CA, Knutson MD (2010). ZRT/IRT-like protein 14 (ZIP14) promotes the cellular assimilation of iron from transferrin. J. Biol. Chem..

