# Supplementary material for: The zinc transporter Zip14 (SLC39a14) affects Beta-cell Function: Proteomics, Gene expression, and Insulin secretion studies in INS-1E cells
Source: Sci Rep. 2019 Jun 13;9:8589. doi: 10.1038/s41598-019-44954-1 (PMC6565745; doi:10.1038/s41598-019-44954-1)
Supplement: Supplementary file 1 — Supplementary material [file 41598_2019_44954_MOESM1_ESM.pdf]

The zinc transporter Zip14 (SLC39a14) affects Beta-cell Function: Proteomics, Gene expression, and Insulin secretion studies in INS-1E cells

Trine Maxel[*], Kamille Smidt, Charlotte C. Petersen, Bent Honoré, Anne K. Christensen, Per B. Jeppesen, Birgitte Brock, Jørgen Rungby, Johan Palmfeldt, and Agnete Larsen.

*** Correspondence:** Trine Maxel: tmj@biomed.au.dk

Supplementary Material 1

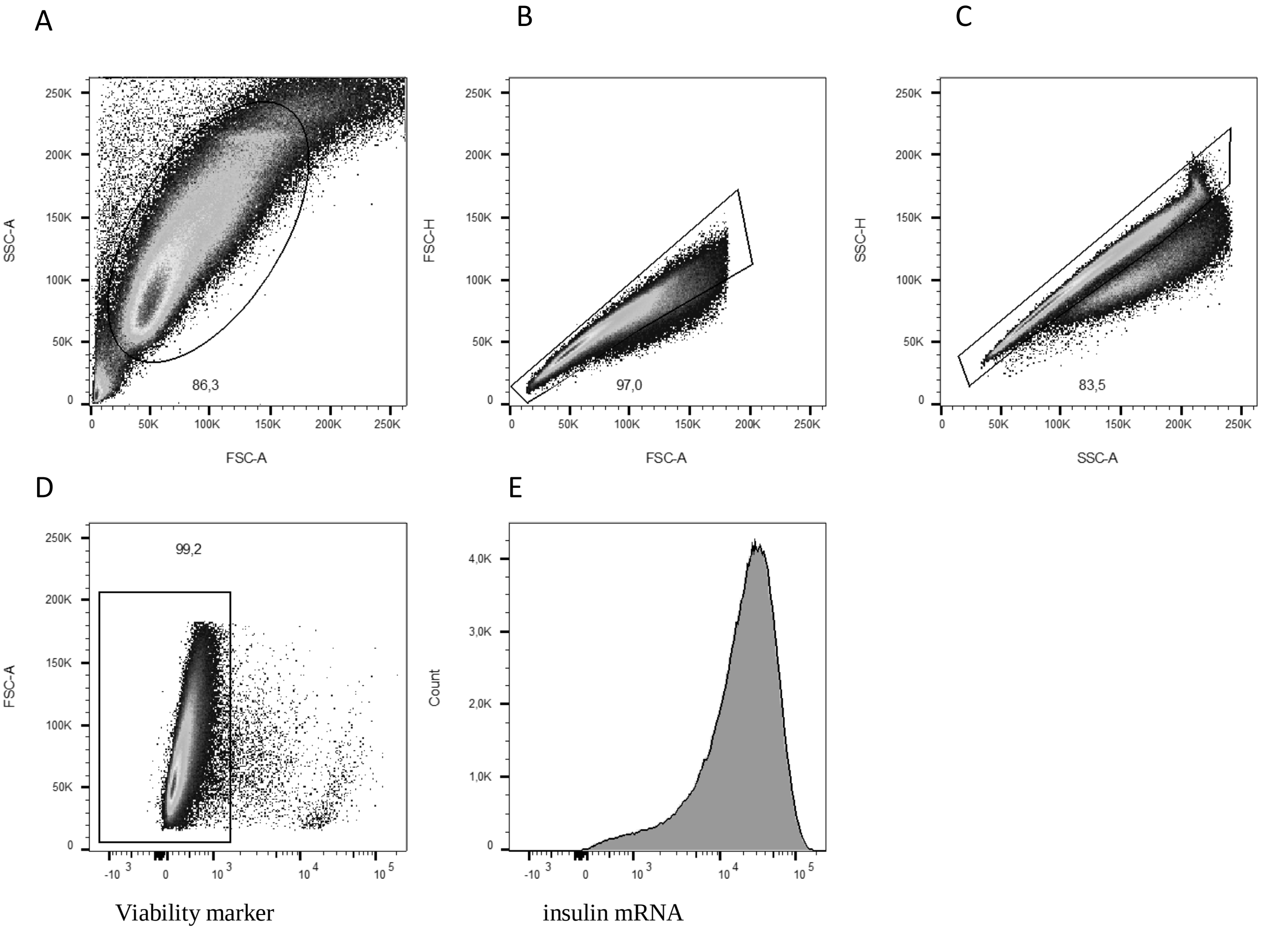

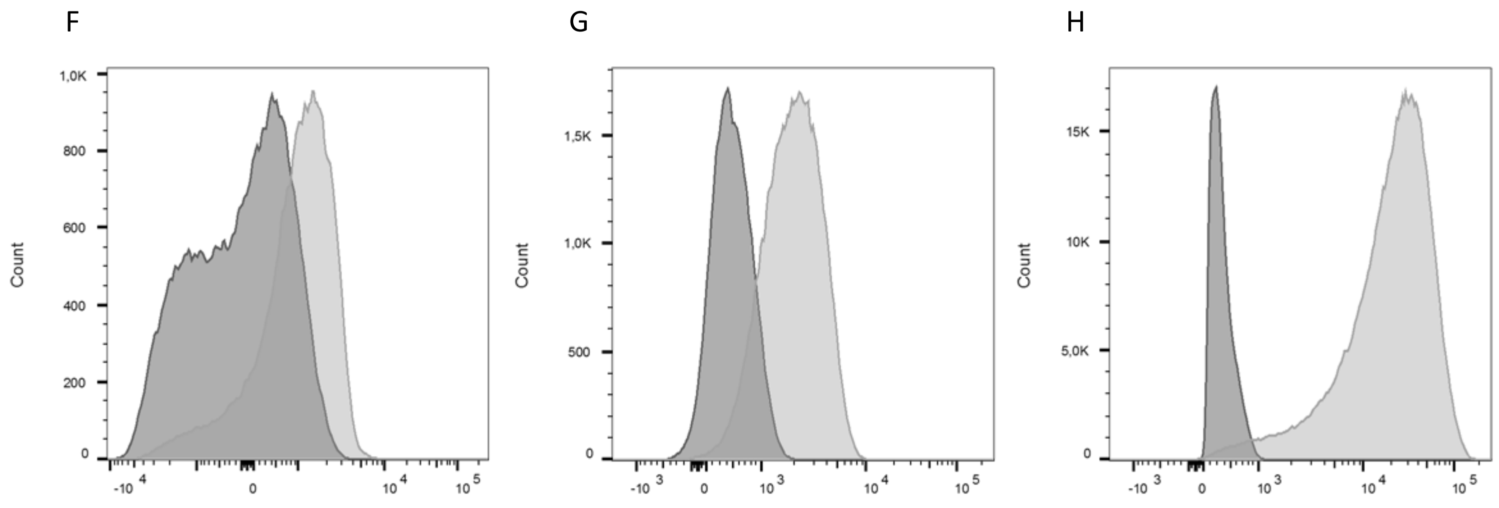

FMO control Zip14 mRNA FMO control Znt8 mRNA FMO control insulin mRNA

Supplementary 1. Flow cytometric gating strategy for experiments using PrimeFlow RNA Assays. Gating was performed by excluding debris (**A**) and cells aggregated in both forward- and side-scatter dimensions (**B** and **C**), and by eliminating dead cells (**D**). Example of insulin (Ins) mRNA signal after gating (**E**). Fluorescence minus one (FMO) controls were included (**F**, Zip14; **G**, Znt8; **H**, insulin). Light gray histograms correspond to the indicated mRNA probe, while dark gray histograms are the corresponding FMO controls. One representative example following incubation of cells in 11 mM glucose is shown. Three hundred thousand cells were collected per sample.

Supplementary Material 2

**Gene name and ID number**	**Forward primer (5´–3´)**	**Reverse primer (5´–3´)**	**Annealing temperature (°C)**
beta-actin (ID: 81822)	CTACAATGAGCTGCGTGTGGC	ATCCAGACGCAGGATGGCATG	62
CycA (ID: 25518)	AGGTCCTGGCATCTTGTCCA	CTTGCTGGTCTTGCCATTCC	59
HSP90ab1 (ID: 301252)	GATTGACATCATCCCCAACC	CTGCTCATCATCGTTGTGCT	57
Bax (ID: 24887)	GTGAGCGGCTGCTTGTCT	GTGGGGGTCCCGAAGTAG	58
Bcl-2 (ID: 24224)	GCACTTTGCAGAGATGTCCA	ATGCCGGTCAGGTACTCAG	57
Ins (1 and2) (ID: 24505 and 24506)	CGCTTCCTGCCCCTGCTGGC	CGGGCCTCCACCCAGCTCCA	68
MT1A (ID: 24567)	TCCCGACTTCAGCAGCCCGA	GCCCTGGGCACATTTGGAGC	63.5
MT3 (ID: 117038)	TGGTTCCTGCACCTGCTCGG	CACCAGGGACACGCAGCACT	63.5
Znt1 (ID: 58976)	CGCTTCGCCCGGAGGACTCA	CACGCCGACGCTAAGCACCA	63
Znt3 (ID: 366568)	TCCTCTTCTCTATCTGCGCCC	TGTGCGGAGGCAACGTGGTAA	62
Znt5 (ID: 294698)	GCACAGCTCTCAGTCGGTCCC	CCCATGACCAAGGCGGAGCA	64

Znt8 (ID: 299903)	GGTGGACATGTTGCTGGGAG	CACCAGTCACCACCCAGATG	61
Zip6 (ID: 291733)	ACACTTCCACGACACGCTGG G	TGGCAAGGCCGTCGCTGAAA	61
Zip9 (ID: 314275)	GCTGGCCTGGAGCGGAATCG	TAGGGAGGACATGGACGGTGG C	66
Zip13 (ID: 295928)	TGGGGCTGTGGGTCATCGCT	TCCGGACCACGGCTCTCAGG	58
Zip14 (ID: 306009)	TCTGCTCAACAGCGTGGGCG	GGGGTGCAAGCCCGGGAATC	64

Supplementary 2. Rat Primer sequences used for real-time PCR.
Abbreviations: CycA, cyclophilin A; Ins, Insulin; MT, methallothionein.

Supplementary Material 3

Detailed description of proteomic analyses.

Preparation of samples for targeted and large scale proteomic studies:

INS-1E cells were grown and transfected (*Zip14* siRNA and control siRNA) as previously described. Cells were scraped into 500 µl of ice-cold PBS with inhibitors (Halt Protease and phosphatase inhibitor cocktail 100×, Thermo Fisher Scientific, Denmark). Samples were centrifuged, then protein pellets were dissolved in lysis buffer (Tris/HCl, 20 mM; EGTA, 5 mM; NaCl, 150 mM; glycerophosphate, 20 mM; NaF, 10 mM; Triton X-100, 1%; and Tween-20, 0.1%) containing inhibitors (Halt Protease and phosphatase inhibitor cocktail 100×). Samples were sonicated on ice and centrifuged to remove tissue particles and intact cells. Samples were pooled 6 and 6 in order to obtain sufficient protein concentration, which were measured using Pierce BCA Protein Assay Reagent kits (Bie & Berntsen A/S, Denmark).

Targeted analysis of Zip14:

Using selected reaction monitoring (SRM) mass spectrometry (MS), the protein level of Zip14 was measured by specific targeting of a unique Zip14 peptide sequence, using the following procedure.

SDS-PAGE, in-gel digestion, and C18 purification:

Five replicate samples of Zip14 siRNA- and control siRNA-transfected cells were included. Protein (25 µg from each sample) was heated at 65°C for 15 min, then separated by SDS-PAGE (Criterion TGX gel, 4–20%, Bio-rad, Denmark) and visualized using Coomassie brilliant blue staining and an Image Quant LAS 4000 Transilluminator, to confirm that a comparable amount of protein had been loaded in each lane. A gel piece corresponding to 60–100 kDa was cut from the gel using a scalpel. Gel pieces were destained in 50% acetonitrile (AcN), followed by reduction and alkylation using 50 mM tris(2-carboxyethyl)phosphine (TCEP) and 100 mM iodoacetamide. Samples were subsequently destained and digested using trypsin (Trypsin Gold, Promega, Denmark) overnight at 30°C in 2.25 mM ammonium bicarbonate, followed by protein extraction using AcN and trifluoracetic acid. The extracted protein was evaporated and purified using C18 spin columns (Pierce C18 Spin columns, Thermo Scientific, Denmark), according to the manufacturer's instructions, followed by evaporation.

Measurement on a triple quadrupole instrument (Triple Q MS), data analysis, and statistics:

Peptides were analyzed on a nano-liquid chromatography (LC)-MS/MS system consisting of an Ultimate 3000 LC (Thermo Fisher Scientific) coupled to a TSQ Vantage triple quadrupole mass spectrometer (Thermo Fisher Scientific, Waltham, MA, USA), as previously described [1]. Separation of the peptides was conducted using C18 reverse phase LC on a trap column (2 cm; particle size, 5 µm; ID, 75 µm) and an analytical column (10 cm; particle size, 5 µm; ID, 75 µm) mounted in a Thermo Scientific Nanospray Flex Ion Source with a liquid junction, using a steel emitter. The eluents used were Buffer A (H_2O, 2% AcN, 0.1% formic acid (FA)) and Buffer B (AcN, 5% H_2O, 0.1% FA). Peptide elution was performed using a linear gradient of 0–39% Buffer B over 40 min. Electrospray ionization in positive ionization mode was applied using a spray voltage of

1700 V. Capillary temperature was set to 200°C, and selectivity for both Q1 and Q3 was 0.7 (FWHM). Argon was used as the collision gas, with the pressure in Q2 set to 1.5 mTorr. The peptide SEFQEFCPTILQQLDSR is unique for the rat Zip14 protein (gene name, *Slc39a14*), and was analyzed (m/z = 1054.50, z = 2) to quantify Zip14 expression using an isotopically labeled peptide standard of the same sequence (JPT Technology, Berlin, Germany). Raw data files from the LC-MS/MS analysis were imported into Skyline software, and each file was manually inspected to ensure correct retention time, peak boundaries (integration), and ranking of transitions. The rdotp-value for the four transitions was 0.99, indicating close to identical ranking across the range of light and heavy peptides. The ratios between the endogenous (light) and internal standard (heavy) peptides were calculated using Skyline. Subsequently, data were exported to Microsoft Excel for statistical calculations. All samples were analyzed twice by Triple Q MS. One sample was excluded from both the targeted and the large-scale study because of an outlying Zip14 concentration. The mean ratio (endogenous Zip14 specific peptide/heavy labeled standard peptide) for each group was calculated, and the groups were compared using the Mann-Whitney U-test (Microsoft Excel and GraphPad Prism 5; level of significance, $p < 0.05$).

Large-scale proteomic analysis:

The regulation of cellular proteins was evaluated following Zip14 silencing using a large-scale proteomic approach. The following procedure was used.

Tandem Mass Tag (TMT) Labeling and peptide purification:

Seventy micrograms of protein was used, and *Zip14* siRNA- and control siRNA-treated cells were compared (n = 4 replicates). An isobaric tag was added to each sample using a TMT 10plex Mass Tag Labeling Kit (Applied Biosystems, Foster City, CA, USA), according to the manufacturer's protocol. Briefly, samples were precipitated with ice-cold acetone, followed by protein digestion with trypsin overnight at 37°C. Samples were labeled with different TMT label reagents, included in the TMT Mass Tag Labeling Kit, and combined into one sample. They were then purified using strong cation exchange chromatography, followed by separation of peptides by isoelectric focusing on an Immobiline DryStrip (pH 3–10, GE Health Care Life Sciences, Uppsala, Sweden) using a Multiphor II unit (Pharmacia Biotech AB, Uppsala, Sweden). The DryStrip was cut into 10 pieces, and the peptides were extracted using AcN and trifluoracetic acid. Peptides were purified using C18-LC (Pierce C18 Spin columns), as recommended by the manufacturer, and evaporated before further analysis.

Nano-liquid chromatography and mass spectrometry analysis:

The peptide mixtures were separated by nano-LC (Ultimate 3000, Dionex) coupled to a mass spectrometer (Orbitrap Fusion, Thermo Fisher Scientific, Bremen, Germany) through an EASY-Spray nano-electrospray ion source (Thermo Scientific). A µ-Precolumn (300 µm × 5 mm, C18 PepMap100, 5 µm, 100 Å, Thermo Scientific) and an analytical column (EASY-Spray Column, 500 mm × 75 µm, PepMap RSCL, C18, 2 mm, 100 Å, Thermo Scientific) were used to trap and separate the peptides, respectively. The peptides were eluted with a flow of 300 nl/min using a 125 min gradient by mixing Buffer A (0.1% FA) with Buffer B (80% AcN, 20% H_2O, 0.1% FA). The gradient steps were performed with the following amounts of Buffer B: 6% (at 0 min), 12% (3 min), 28% (88 min), 40% (95 min), 90% (100 min), 90% (110 min), 6% (111 min), and 6% (125 min). The MS detection consisted of a full Orbitrap scan (m/z 380–1500) at a resolution of 120,000, with an

automatic gain control (AGC) target of 2×10^5 and a maximum injection time of 50 ms. Up to 10 data-dependent MS^2 scans were performed in the linear ion trap in the mass range 400–1200 m/z, with CID energy at 35%, an AGC target of 1×10^4, and a maximum injection time of 50 ms. The precursor ions were isolated using the quadrupole, with an isolation window of 0.7 m/z. The reporter ions were isolated with a window of 2 m/z and detected with data-dependent MS^3 using synchronous precursor selection in the Orbitrap in the mass range 120–500 m/z, with a HCD collision energy of 65%, and acquired at a resolution of 60,000, with an AGC target of 1×10^5 and a maximum injection time of 120 ms. Dynamic exclusion was set to 30 s or 70 s, in first and second replicate analysis, respectively.

Database searches and statistics:

The raw MS data files from the two separate TMT studies were processed, and all generated peak lists from the same study were merged and analyzed using Mascot v. 2.5.1 (Matrix Science, London, UK) in Proteome Discoverer 2.1 (Thermo Scientific). In Proteome Discoverer 2.1, the following default workflows were applied: "Processing workflow for SPS MS3 reporter ion-based quantification" and "PWF_Fusion_Reporter_Based_Quan_SPS_MS3 _Mascot_Percolator". The MS data were searched against the SwissProt *Rattus* database downloaded in May 2016, which contained 25,741 proteins (including unreviewed sequences). Mascot was used for protein identification. Full scan tolerance was 8 ppm, MS/MS tolerance was 30 mmu, and up to two missed cleavages were accepted. Oxidation on methionine was set as a dynamic modification, while TMT-10-plex on lysine and N-terminal, and carbamidomethyl on cysteine, were chosen as static modifications. Only protein-unique peptides (Unique+Razor) and those consisting of at least six amino acids were included. Normalization to the summed intensities of the TMT signal was applied to compensate for possible variation in the starting material. The false discovery rate (FDR) criterion for peptides was $q < 0.01$. Only proteins with an identification score ≥ 30 (corresponding to a protein identification significance of 0.001) were considered, as well as proteins with at least three quantitative scans and one protein unique peptide, yielding 3431 proteins.

Mean protein ratios were calculated for each protein as the mean abundance in the four *Zip14* siRNA-treated samples divided by the mean abundance in control siRNA-treated samples. A significant fold change was defined as 2× global standard error of the 3431 proteins ($2 \times 0.068 = 0.136$). FDR was calculated from p-values (estimated by Student's t-test) using Benjamini and Hochberg's method [2]. A FDR < 0.05 was used. Fifty-two proteins met the fold-change and FDR criteria. For gene ontology analysis, to obtain protein groups, wider criteria, defined as a p value < 0.05 and a significant fold change, were used, yielding 121 proteins. For ontology analysis, the Functional Annotation tool from the Database for Annotation Visualization and Integrated Discovery (DAVID) was used (DAVID 6.8 Beta; https://david-d.ncifcrf.gov/). The standard settings of DAVID, with the addition of protein interactions, were applied with the stringency set as medium. The total list of the 3431 proteins found within the samples was selected as background. An enrichment score (ES) ≥ 1.3, equal to 0.05 on a non-log scale, was set as the level of significance [3]. Three protein lists were analyzed for functional enrichment; one included both up- and downregulated proteins and two consisted of only either down- or upregulated proteins.

Reference List

(1) Birkler RI, Nochi Z, Gregersen N, Palmfeldt J. Selected reaction monitoring mass spectrometry for relative quantification of proteins involved in cellular life and death processes. J Chromatogr B Analyt Technol Biomed Life Sci 2016 Nov 1;**1035**:49-56.

(2) Benjamini Y, Drai D, Elmer G, Kafkafi N, Golani I. Controlling the false discovery rate in behavior genetics research. Behav Brain Res 2001 Nov 1;**125**(1-2):279-84.

(3) Huang dW, Sherman BT, Lempicki RA. Systematic and integrative analysis of large gene lists using DAVID bioinformatics resources. Nat Protoc 2009;**4**(1):44-57.

Supplementary Material 4.

The full list of identified proteins in INS-1E cells by proteomics.

Accession	Gene name
D4A4W6	Slirp
A0A0G2JZD6	Resp18
F1M9I3	Muc13
F1M1H0	Dera
G3V6S2	Aco1
A0A0G2K1L4	Reps2
D4A8G5	Tgfbi
Q566E4	Hnrnpr
F8QYX0	Chga
F1LMH0	Sept3
G3V6C3	Pbdc1
E9PTF3	Noc2l
A0A0G2K3U1	Ampd2
D4A193	Reep1
A0A0G2JSL7	Pdp2
A0A0G2K2Y2	Wnk3
D4AE80	Dcp1a
D3ZQ02	Wdr37
F1LP80	Vgf
F1M0R1	Rnf213
D3ZRX9	Cnn2
D4A7R3	Nup205
B0BN20	Tspan6
D3ZUY8	Ap2a1
A0A0G2K6H5	Cfdp1
D3ZJN9	Evi5
D3ZDD7	Strbp
A0A0G2K5U7	Cdk12
A0A0G2K2M9	Srrm2
D3ZNI3	Pdcd11
B2RYC9	Gba
D3ZYT2	Mrps5
M0R3M4	Ranbp2
F1LPG3	Pacs1
D4AB70	Edem3
B2RZD5	Rpl22l1
Q4KLK7	Nop56

D4A0Y6	Mki67
Q9QXI2	Rfc2
Q6P9W6	Rab3b
A0JPQ5	RGD1560212
B5DEM3	Tmed4
F1LNI8	Camk2b
D3ZDG0	LOC681282
D3ZIF0	Zfp512
F7FF45	Numa1
G3V784	Adpgk
G3V827	Ccbl1
F1LW91	Numa1
G3V836	Clu
G3V8A4	Mtr
A0A140TAA1	Igsf8
D3ZD46	Dhx58
A0A0G2K0T5	Micu1
A0A0U1RRT8	Sept6
A0A0G2JYU2	mrpl11
A0A0G2K8H0	Caprin1
Q6MG72	Ehmt2
A0A0G2JZY3	Reps1
D3ZK96	Papola
B6ID02	Gprc5c
A0A0G2K6T9	Pcdh1
B0BN81	Rps5
F1M6V1	Hp1bp3
D4A7X1	Mrps16
O35823	Abi2
D4ACW1	Nop2
D3ZJY1	Mrpl28
M0RCH8	NEWGENE_1359295
A0A096MJI4	Gigyf2
F1LT70	Pank2
D3ZUP8	Lmbrd2
D4ABM5	Mrps34
A0A0G2JX16	Qrich1
G3V6P2	Dlst
D4A4Z9	Ktn1
D3ZBW5	LOC685203
D3ZRN2	Med1
F1M4Q5	Ssh2
B2RYJ3	Cul4a
Q5D1N7	Rcan3
B0BMU7	Elp5

A1L1I6	Rtn3
D3ZZN4	
B5DEL8	Ndufs5
G3V7I0	Prdx3
H9KVF6	Stk10
Q5PPP1	Clta
A0A0G2KAT4	Ddx39b
Q6AYA4	Galnt14
D4A833	Mrps30
D3ZPU3	Hsd17b12
B2RYS0	Cox7a2
Q5XIB7	Tsen34
G3V9K3	LOC103690070
A0A0G2K5E4	Dnaja3
Q5RJM3	Igfbp7
B1H297	Ddx31
G3V7R3	Gmeb2
F1LW77	Rab33b
F1LSG0	Wasl
D3ZMI8	
Q4G067	Mrpl44
A0A0G2K0D5	Osbpl7
A0A0G2JV04	Gga3
R9PXY6	Pfkfb2
F1M8Y4	Deptor
D3ZU85	Frmd4a
Q5PPK6	Nop58
Q6GT74	Bsg
Q7TPJ5	Impad1
M0R4H5	Pdlim4
Q5XHZ8	Cog3
D3ZC55	Hspa12a
A0A0G2KAD8	Syp
F1LRQ6	Cdc23
D3ZFT1	Rfc1
G3V720	Kcnh6
Q499T8	Itln1
F1LNT1	Itpr1
Q68FY4	Gc
F1M1D2	Tab3
A0A140TAF5	Clcc1
Q68FT7	Farsb
A0A0G2JSH2	Bdh1
A0A0G2QC27	Nol6
G3V6H9	Nap1l1

F1LSM7	Scaf4
A0A140TAE0	Mvp
A0A0A0MXY9	Akr7a2
D4A0X3	Btbd8
F1M049	Atxn2
F1LNK0	Map2
A0A0G2JV31	Xpnpep1
Q1JU70	Ythdf2
G3V7J2	Prkra
G3V8P5	RGD1310127
F1LMV9	Coro2b
D3ZE16	Idua
A0A0G2K1E3	Kmt2b
B0BNG3	Lman2
D3ZVD8	Hdac6
F1LNF0	Myh14
A0A0G2JYC7	Hdgfrp2
D4A962	Hnrnpul1
G3V6F5	Elac2
Q5BJZ3	Nnt
B0K021	Rpl7a
F1LPS0	Synj1
G3V6D3	Atp5b
A0A0G2K350	Napg
G3V9S9	Sec24d
F1LNF7	Idh3a
Q5EB90	Polr2c
Q6T487	Actn1
F1LPC6	Cpd
Q5U214	Snrpa
F1LSW6	Ppip5k1
G3V6V1	Rnpep
A0A0G2KAM3	Pdhb
Q5XI39	Cryzl1
D3ZSX6	Paxip1
F1LMA4	Adrbk1
D4AC36	Eif3f
A0A0G2K6R7	Wash1
F1LPB3	Acsl5
A0A0G2K2B0	Phc3
P70527	RT1.Ag
D3ZF52	RGD1565767
Q0D2L2	Mrps22
A0A0G2JU77	Eif3k
G3V829	Fubp3

A0A0H2UHR9	Obfc1
F1LRK0	Snap91
A0A0G2JX76	Asap1
D3ZNZ9	Hist3h2ba
B5DES0	Snrpd2
D4A6H8	Ctnna2
A0A0G2JZZ4	Selm
B5DF02	Eed
F2Z3T9	U2af2
D3ZQ74	Plod1
D3ZZX1	Inpp5a
D3ZLL8	LOC691716
F1M9W9	Trappc8
A0A0G2JXT0	Vps26a
Q5U355	Itfg1
D4AD82	Arhgap35
D3ZIN7	Mrps23
Q66HP4	Stau1
A0A0G2K7W4	Map4k4
Q4FZU0	Acp6
G3V7J8	Kif3c
F1M949	Ckap5
G3V648	Agpat3
B2RZ96	Ube2r2
F1M9Y9	Rbpms
D4A719	Snx12
G3V8E2	Strip1
B5DF24	Uck1
A0A0G2K9Q6	Atp2b3
D4AEB3	Setx
A0A096MJL6	Pgk1
B2GV58	Lsm14a
F1M907	Dlg2
A0A0G2JZ60	Fsd1l
D4A2D8	Lsm7
G3V7X2	Scg2
A0A0G2K0J4	Nipbl
D4A777	Fam114a1
A0A0H2UHT5	Ppm1g
A0A0G2K7B9	Car8
A0A0G2K3V7	Top1
G3V8T5	Ruvbl2
A0A0G2JSQ9	Pelp1
G3V6A6	RGD1566265
B5DEL9	Rps7

F1LQC1	Acox1
A0A0H2UHE1	Suclg1
A0A0H2UHS7	Rpl18
F1M6J5	Jade3
B0BMW0	Rab14
D3ZTF1	Slc4a1ap
D4A9U6	Ubr2
A0A0G2K8Z9	Kif13b
Q6PDV8	LOC100360057
F2Z3R2	Hnrnpl
A0A0G2K964	Ube2e3
Q6AYC9	Asb6
D3ZQQ2	Wiz
D4A781	Ipo5
A0A0G2JW88	Map4
E9PT79	Tsn
F2Z3T7	LOC103694869
Q5EBA7	Hgfac
B5DFJ2	Fam3a
F1MAM8	Ddx20
Q68G39	Cdk16
Q9ES32	Tfa
A0A0G2K719	Ddx3x
D4AD33	Fam103a1
D3ZRA3	Acaca
F1LMA7	Mrc2
D3Z8K0	Pdzd11
Q5RKI5	Flii
A0A0G2K7W6	RGD1562402
D4AA91	Cers5
D3ZJW8	Med25
G3V9J7	Rabep1
B2RYN3	Eef1e1
D4A4Y0	Exosc4
B2RYC6	Zfp385a
D3ZF86	Arfgef3
D3ZQH9	Rasa4
G3V9F3	Mprip
Q6DGF2	Clint1
Q06C60	Bola1
A0A140TAF2	Elavl4
A0A140TAJ5	Pi4ka
D3ZXF9	Mrpl12
Z4YNS1	Psme2
A0A0G2K7X3	Nucks1

D3ZSX2	Aff4
D3ZAI0	U2surp
D3ZF64	Abcc3
A0A0G2JVW3	Ankrd17
F1LR36	Supt6h
F1LRT0	Ltbp3
A0A0G2K946	Spock2
A0A0G2JXT8	Flnb
D3ZRN3	Actbl2
A0A0G2K3K2	Actb
Q9ESH1	Psmd4
A0A0G2K9R3	Supv3l1
B2GV62	Mrpl20
F1LSL2	Nup107
M0RE00	Tbca
A0A0G2K9A2	Arpc2
A0A0H2UHK2	Pgrmc1
G3V705	Pqbp1
M0R567	Irf2bpl
A0A0G2JUC9	Herc2
M0RDD7	Chtopl1
D4AA13	Dmxl1
D3ZT52	Pbrm1
A0A0G2KA88	Soga1
A0A0G2JSK5	Itgb1
D4A565	Ndufb5
D3ZU55	Foxk1
F1M8P6	
F1LPC7	Hdgf
G3V9V9	Csrp2
D3ZZA8	Sec24a
A0A0G2K405	Prkar2a
B2RYQ8	Rpl36a
D3ZFY0	Sephs1
B3SVE5	Zwint
Q64538	
A0A096MJL5	Pola1
A0A0G2K0Z7	Gpd2
D4A4K4	Vps13c
G3V6K3	Exosc7
D4ADF5	Pdcd5
Q6B436	Cct5
D3ZN79	LOC686074
F1MAB8	Kif11
Q3ZB99	Tjp2

A0A0G2K3R1	Map3k4
D3ZGD0	Gemin5
Q5XHY0	Ddx18
A0A0G2K977	Golga2
A0A0G2K318	Myo5b
Q499P2	Lta4h
A0A0G2JXV4	Snx17
G3V7A5	Ldlr
A0A0G2K648	Wdr61
A0A0G2K1M0	Taok2
A0A0G2K2J9	Ctage5
D3ZMY7	Nt5c2
G3V6P7	Myh9
R9PY05	Nfasc
A0A0G2K5E6	
B5DEM5	Rpl14
G3V918	Gart
D3ZMX6	Sntb2
Q3B8P4	Dcps
A0A0G2K0M8	Ncam1
M0R608	Rtn1
B5DFC6	Gnpda1
F1LSV0	Sema4b
F1LRL9	Map1b
Q80W83	Ppp2r5b
F1M7T1	LOC100361934
Q62801	Srp54a
A0A0G2K2U5	Rilpl1
Q7TP06	Cep192
A0A0G2K543	C2cd5
M0R9B9	Ubxn7
A0A0G2K6J3	
A0A0G2JTT6	Herc1
A0A0G2K2B8	Ywhah
D3ZIT4	Anapc7
B1WBQ7	Msh2
G3V852	Tln1
D3ZZ95	LOC100361060
B1WC65	Haus3
G3V6H2	Prpf8
D4ADB4	Cggbp1
F1LRK4	Grsf1
A0A0G2JZ87	Sfswap
B0VXR4	Mapk8ip3
Q4V8I6	Rpl11

B2RYI2	Srp68
F1LPP0	Amph
A0A0G2KBC7	Pfkm
A0A0H2UHF6	Arl6ip5
A0A0G2KB92	Dclk1
A0A0G2JZH0	Cab39
A0A0G2JYT9	Rfx3
G3V8C8	Ttl
F1M7Z1	Edc3
A0A0G2K1Y8	Sptan1
B5DEZ6	Gnpda2
D4A053	Shroom2
M0R6L4	LOC100365839
F2Z3Q8	Kpnb1
B5DFJ4	Vps18
Q5RKH8	Ppil2
A0A0G2JSP8	Ckm
Q499V0	Commd7
G3V6T7	Pdia4
D4A206	Tcof1
B2RZA4	Ap1m2
D3ZPJ1	Klhl11
A0A0H2UHD4	Slc30a8
D4ABV5	Calm2
A0A0G2K2G8	Tpm4
D4A9D6	Dhx9
B1WC49	Api5
Q6AYI1	Ddx5
B2RZ09	Manf
A0A0A0MXZ5	Bap1
D3ZS58	Ndufa2
B2RYG5	Taf15
D3ZQY4	Fryl
G3V8T9	Bax
A0A0G2K6Y9	Stx7
F1LML9	Baiap2
Q32PY7	Nudcd3
D3ZM57	Golim4
Q4FZS2	Bub3
A0A0G2K1W1	Rab11fip5
B2RZD6	Ndufa4
A0A0G2K3I9	Dusp3
A0A0H2UHR5	Bckdk
F1M9N7	Agfg1
B1WC00	Zfr

Q8VHC1	Cst6
I6LBX7	Pcdhgb8
Q32Q88	Fbxo22
A0A140TA99	Tceb2
A0A0G2JXA8	Flot2
D3ZQD3	Ogdhl
A0A0G2KA48	Mrpl37
A0A0G2JYT1	Erc1
D4A0T0	Ndufb10
B1WBP6	Plbd2
Q99PS2	Csnk1e
S4VP54	Map2k4
Q4KLI7	Sf3a3
D4AE49	Skiv2l2
D4AE56	Ptges2
A0A0G2JUB0	Fam3c
A0A0G2K435	Dnajc7
Q5U3Z7	Shmt2
A0A0H2UI07	Pklr
F1M3D3	Hnrnpm
F1LPB8	Entpd5
D4ACB8	Cct8
A0A0G2JWD2	Fxr1
A0A0G2K0W3	Ttyh3
M0RDF7	Bysl
B2GV96	Ccdc115
B6DYQ4	Mgst1
D3ZY40	Pcf11
A0A0G2JW85	Rasa3
G3V824	Igf2r
R9PXV2	Ralgapb
M0R5T5	Shank2
D3ZXA6	Pdpr
Q2KMK7	
F1LRW6	Atp2b1
D4AE17	Zak
F1M2K3	Sumo2
F1M4J1	LOC100294508
Q5BK56	Gstm4
F1LSK6	Cyp20a1
D3ZJU0	Arid2
A0A0G2JSW3	Hbb
Q6AXY8	Dhrs1
E9PTR4	Ubap2l
A0A0G2K7L4	Itpk1

D3ZTK5	RGD1563835
F1LTS8	
G3V8G2	Psmd5
Q5FVS1	Fth1
D4A9P7	RGD1565363
D3ZWL9	Dido1
Q5BKA1	Metap2
D3ZH66	Taf5
A0A0G2K220	Kdm3a
F1LRB7	Tsg101
B2RYS6	Prtfdc1
G3V963	Tmem132a
D4A7G5	Ganc
D3ZG43	Ndufs3
F1LP91	Gsk3a
A8QIC3	Dnajc9
M0RBY8	Brap
G3V681	Mcm4
A0A0A0MXU4	Emc2
M0R7T1	Phactr4
V9GZ82	Ap3m2
A0A0G2JZE6	Rack1
D3ZJ91	Ddhd2
Q6IRH6	Slc25a3
M0R8J3	
A0A0H2UHZ2	Nap1l4
D3ZZM3	Cog4
B5DES1	Rpl23a
D3ZLT1	Ndufb7
Q3MIE9	Sms
M0R9Q1	Rbm14
G3V9T9	Ptpn6
E9PU44	Smarce1
G3V886	LOC100910779
F1LPC5	Tmem259
B2RYU2	Rpl12
R9PXU4	Txnrd1
D3ZRM9	LOC100360491
B3STT9	Kdm1a
M0RCA3	Zfpl1
Q63116	Kit
D3ZXY2	Pdzd8
D3ZCT5	Pald1
D4AD36	Fchsd1
B5DFF4	Vps37c

D4A105	Tmem63b
G3V6Q6	Gna11
A0A0G2JW52	Prmt3
M0R3L1	Mast4
A0A0G2JZ79	Sirt1
R9PXT0	Uba3
A0A0G2K8Q2	Gramd3
M0R7P0	LOC100912027
A0A0G2JTF5	Usp34
G3V781	Mre11a
G3V8M1	Pold1
D3ZTW7	Atpaf2
D4A0M3	Phc2
B1WC61	Acad9
E9PSJ4	Spag9
Q5BK37	Arhgef1
A3KNA0	Aqr
D3ZJK6	Trappc9
G3V7V6	Retsat
D4ADM2	Pan3
D4A3P0	Ybx2
D4AB23	Noc3l
F1LXA0	Ndufa12
G3V949	Clip2
Q6MG28	RT1-CE16
B0BNE6	Ndufs8
A0A096MK30	Msn
D3ZRE3	Csnk1a1
D3ZGJ0	Dhx32
A0A0U1RRV0	Pja1
F1LRP7	Ago2
D4A6T5	Cutc
Q6AY21	G3bp2
Q6LDS4	Sod1
A0A0G2JTN8	Atp5g2
D3ZQN3	Pnmal2
A0A0G2QC56	Coro2a
B0BN68	Mrps9
Q3MID9	Ogfr
M0R7M8	Mapre2
Q6TXF6	Decr2
F1LT10	Mllt4
A0A0G2JSM7	Add1
Q6XLI7	Rbm12
A0A0G2K995	Fblim1

D4A7U6	Lsm3
A0A0G2KAT5	Ptk2
D3ZVK3	Trmt6
D3ZDE4	Dguok
F1M1X9	Cdk13
D4AB03	Fam120a
A0A0G2K890	Ezr
Q5M963	Cmas
Q7TPI5	Glud1
G3V678	LOC100912534
Q6GQY2	Mcfd2
B4F7D5	Tmem181
G3V614	Kif1bp
D3ZCG3	Mon2
D4A9D8	Osbp
D3ZES9	Prc1
A0A0G2JSM4	Bet1l
A0A0G2KA20	Mcf2l
G3V936	Cs
A0A0G2K4U6	Ilf3
D4A4P8	Ttyh2
D4AEG7	Tbc1d13
Q6MG66	Lsm2
O70188	Nfic
B1WBX4	Zfp2
D3ZAG3	Map9
F1M8A5	Hypk
D3ZUK4	Trim33
R9PY09	Pacsin2
Z4YNP9	Wdr7
Q6P7B6	Efnb1
B0BNB9	Htra2
Q6PLD8	Cpg2b
G3V7L6	Psmc2
B5DF48	Tyw1
F1LP45	Apbb1
F8WFS9	Add2
D3ZCZ9	LOC100912599
D4A8M7	Ncaph
D3ZXS8	Ube2k
A0A0G2JVW5	Huwe1
B2RYJ6	Ttc9
Q32PZ7	Srp72
F1LZX5	Hectd4
D4A3V2	Ndufa6

Q642A9	Vps16
F1LQZ9	Map6
F1LN92	Afg3l2
G3V8M8	Parg
B1WBZ5	Nusap1
F1LXP8	Arhgap18
A0A0G2JY12	Pxn
B1WBS4	Vps26b
Q5I0M7	Hnrnpa1
M0RD75	Rps6
A0A0H2UHV9	Copg2
M0R711	Ptprs
A0A140UHW6	Trmt1
A0A0G2K5L1	Fat1
D3ZYG0	Vav2
A0A0G2K9M4	Wdfy3
G3V6X7	LOC103690091
Q3SWT9	Nars2
D4AAM0	Tnpo3
D4A6N9	Asun
B4F7B6	Bmi1
D3Z8R4	Rbm25l1
B5DF65	Blvrb
D3ZT90	Gcdh
Q5XIU4	Bcap29
B1WBN7	Fam20c
D3ZCR4	Ppp4r3b
D3ZVR9	Pgm5
F1LRV6	Gmpr
A0A0G2K757	Rpn2
Q6AYM8	Gpaa1
D3ZSN4	Zfp330
F1M9V6	Myo10
F1M9D6	Stat1
B2RYJ7	Actr1b
B4F774	Gdap1l1
D3ZJW5	Zfp629
D3ZJ32	Esyt2
D4A2F6	Nhlrc3
D4A604	Pigt
F1LYM2	Ube2d3
Q5PQZ9	Ndufc2
A0A096MKH2	Vta1
Q3KRF2	Hdlbp
D3ZTG2	Ttc27

M0R9L2	Cntfr
A0A0G2JWP8	Top2a
B1WBY7	Erlin1
A0A0G2JX62	Srpk2
A0A0G2JSH9	Prdx2
Q99NA6	Idh3B
A0A0G2K9I8	LOC100361025
D3ZS72	Prex1
G3V8T3	Adar
Q6AYT1	Trim5
G3V9N8	Ap1b1
D3ZE15	LOC100911483
Q6P6W6	Ndufa10
F1M062	Larp1
D3ZHB7	Ube3c
Q5D023	Dync1li2
D3ZUC9	Oxsr1
F1MA54	Pdk1
Q6P9X2	Sec11a
B2GV41	Usp39
A9UMV9	Ndufa7
D3ZP96	Mcm2
B6DYQ1	
Q6IRH7	Clpb
A0A0G2K2A0	
D4A899	Vps13a
A0A0G2K7N8	Bptf
A0A0G2K9K2	Tacc1
A0A0G2JT63	Cyfip2
A0A096MIV5	Abcf2
D3ZME3	
D3ZJG4	Pacs2
D4A7M6	LOC100359539
G3V624	Coro1c
Q499S6	Ctsf
B0BNM3	Snx9
G3V679	Tfrc
D4A4Q4	Ociad2
D3ZLK9	Naxd
A0A0G2JYR1	Exoc6b
A0A0G2K9J2	Atp6v1h
D3ZL11	Rbsn
D4A779	Phldb2
B2GUX5	Nt5c3a
D4A305	Ccdc58

Q6IN10	Ece1
B5DEK0	Rprd1b
F1LX07	Slc25a12
Q5RJK6	Inpp1
F1LMX1	Bin1
F1M9G6	Taok1
A0A140TAB8	Igf1r
F1LV01	Wdr19
G3V6W2	Preb
F1LYQ7	LOC680700
A0A140TA89	Gatm
A0A0G2K217	Evl
A0A140TAF7	Poglut1
Q5BMA6	Arhgef12
G3V7V3	Slc27a4
A0A0A0MXZ0	Isca1
D3ZAX5	Cherp
A0A0G2K3B2	Selt
A0A0H2UHU1	Rpl37a
D4A9G1	Rpl3l
E9PT85	Xrcc6
A0A0U1RS25	Upf1
F1M978	Impa1
G3V6B1	Tgfb2
B5DFD1	Nudcd1
A0A140TAE6	Mecr
Q6IN39	Hsd17b4
F1LWZ7	Tbc1d8
Q4KLI0	Smarcb1
D4A3E1	Hnrnpll
Q5PPG2	Lgmn
F1LP30	Mccc1
A0A0H2UI35	Homer1
A0A0G2K782	Zc2hc1a
D4A9Y0	Sdf2l1
D3ZJ81	Fstl4
D3ZKI6	Ralgapa2
D3ZXN8	Anxa10
D3ZXJ3	Zfp78
Q6P6Q3	Nucb1
A0A0G2K261	Iars2
A0A0G2KAU2	Armcx3
F1LW74	Iqgap2
F1LS42	Prkcb
D3ZQ59	Nrd1

B7Z6N5	
F1LSF4	Enpp2
F1M9A7	Acox3
F1MAQ7	Son
G3V8M5	Ppp4c
F1LNY0	Hprt1
G3V714	Scg5
C0JPT7	Flna
D3ZD89	Naa15
F1M4H5	Nova2
A0A0G2JXW5	Bbx
A0A0G2K279	Zfp92
A0A0G2K0M1	Kif15
D3ZZ51	Prrc2b
G3V8V0	Arl2
M0R7E6	Srrt
D3ZVJ3	Kntc1
Q5RJN0	Ndufs7
A0A097BVJ5	Cnp
A0A0G2JWF7	Morc2
A0A0G2JWP1	Cstf2
B5DFN4	Pfdn5
F1M403	Ube2o
D3ZJF9	Gla
A0A0G2K6Q5	Gnl1
D3ZX87	LOC100910017
A0A0G2JSM0	Dnajc2
D3ZGQ1	Tor1aip1
A0A0G2K556	Uimc1
A0A0G2K0W0	Ggps1
Q6P2A5	Ak3
E9PTE5	Zmym3
Q4KMA8	Erap1
A0A0H2UHS5	Bod1
F1LM28	Nf1
A0A0G2JZM8	Clasp2
D4AAU6	Ccdc25
B0BMY2	LOC684097
A0A0G2KB11	Stard10
Q499R1	Ppp2r5d
B2GUW8	Slc25a53
A0A0G2K4T5	Smc5
B1H275	Wbscr22
F1LP22	Atp2b2
A0A0G2K2Z0	Eml4

A0A0G2K9S3	Adrbk2
G3V8U3	Dpf2
F1LTR1	Wdr26
Q9WUF7	Hmmr
D4ACM1	Elp3
B1H241	Ric8a
B5DFH2	Map1s
A1A5S4	Sephs2
Q5M9F7	Actr10
F1LP05	Atp5a1
F8WFP9	Ero1a
F1M1D5	Tbcd
D3ZFH6	Phb-ps1
D4A2B0	Poldip3
A0A0G2K585	Arpc5
F1LXV0	Rps6ka1
E9PT66	Sf3b3
D4A746	Gmppb
M0R982	Uxs1
D3ZGE6	Cttn
Q4VBH2	Trnt1
D3ZGY1	Pym1
D4A164	Nebl
R9PY07	Pitpnb
G3V943	Dnaaf5
D3ZIX4	H1fx
D3ZQE8	Xpo5
D4A944	Snrnp40
C7C5T1	ChREBP
F1LNP1	Arid1b
F1M953	Hspa9
D3ZKT8	Hddc2
D3ZV60	Cenpe
Q6P783	Pfkl
D3ZIY3	Ythdf3
A0A0G2K658	LOC102556347
B5DEG8	Sec24c
D3ZC01	Rbbp5
D3ZDR2	Chmp6
D3ZFE9	LOC100359687
F1MAH5	Ppp6r3
Q4V8C0	Prim2
A0A0A0MXX8	Fcho2
D3ZPR0	Cse1l
G3V7X4	Abca2

Q496Z1	Bicd2
D4AAU4	Rprd1a
D4A3E8	Mrps27
B6DYQ0	Gstk1
A0A0G2KAP1	Ero1b
F7EZZ0	Dap3
D3ZVQ0	Usp5
D3ZYT5	Myt1
D3ZFQ8	Cyc1
G3V7J0	Aldh6a1
D4A1P2	Rpl10l
A0A0G2JZY0	Lsm14b
A0A0A0MXY0	Gba2
D3ZVH6	Vps41
Q3ZAV2	Ybx1
F1LZX7	Rpl17
G3V9I9	Srek1
D4ABD7	Trip11
D3ZYX8	Cox7a2l
Q32KK2	Arsa
D4ABT8	Hnrnpul2
A5I9F0	Ptprk
D3ZMJ7	Wnk2
D4A1B8	Dctn3
D4A634	Ranbp6
A0A0H2UHZ6	Puf60
A0A0H2UHV2	Med23
A0A0G2JUN5	Impa2
G3V9N0	Pabpc4
F1LMR7	Dpp6
A0A0G2JWG0	LOC103690022
A0A0G2KB52	Map7
D3ZW08	Adsl
Q5U2N1	Nelfa
Q7M093	
D4AEL2	Golm1
A0A0G2K9L9	Mdn1
D3ZKX8	Fam169a
A9CMA3	Mgat5
M0R6J0	Mrpl39
D3ZBG6	Ptrhd1
F1M084	Plcb1
Q499R7	Ppa1
D4A1Q9	Ttll12
A0A0G2K014	Lcp1

F1MAL5	Irs2
A0A0G2K681	Clip1
Q5RK17	Diablo
F1LW16	Btaf1
D4A2X2	Myo1b
D3ZKG1	Mut
Q6DUV1	Prkce
A0A0G2K2L8	Cask
A0A0G2JUU7	Tubgcp3
D3ZDP2	Ict1
A0A0H2UHJ4	Ints12
F1M155	Svil
D4A0T8	Dhrs7
M0R7Q3	Rbpj
M0R665	
A0A0G2K8B7	Eif4a2
Q7TP91	Surf4
D3ZUT9	Ints3
A0A0G2K8C7	Mtmr3
G3V712	Krt7
A0A0G2K386	Parn
D3ZVP6	Ppwd1
D3ZRH1	Oxa1l
A0A0G2K9J0	Tanc2
D3ZA55	Ecd
A0A0G2K7M2	Rad23a
O89045	
D3ZP87	Fam92a1
A0A0G2JSI1	Aldh9a1
D4A542	Gprasp2
A0A0G2K7N9	Arhgap5
D3ZJ92	Prpf40a
G3V7I7	Lrrc40
A0A0G2K1C0	Actr3
Q5UAJ5	ATP8
D3ZCH6	Rnaset2
M0R7U6	Sowahc
A0A0G2KA06	Klhl22
G3V8B6	Psmd1
M0R6L9	RGD1560687
A0A140TAH1	Hgs
Q3KRC3	Srpra
Q56R20	Kpna1
A0A0G2K4C1	LOC103694557
G3V9E0	Mcrs1

B2RZA9	NEWGENE_1308361
A0A0H2UHG4	Cdk18
A0A0G2JT48	Mapre1
B3KU41	
A0A0G2K8V2	Vcl
F7F557	Acot8
D3ZND9	Riok3
F1M9C4	Optn
A0A0G2K6I4	Enah
M0R3K1	Rheb
F1MAA2	Cops7a
D4A8H3	Uba6
Q6P743	Ahcy
E9PT44	Nr3c1
Q9JI56	Snap29
M0RDY6	LOC100360726
D4A133	Atp6v1a
A0A0G2K139	Cand2
F1LMD9	Gak
Q6AYR1	Tfg
D4A020	Med14
A0A0G2K5P8	Glrx3
D3ZTR4	Sumf2
D4A6P1	Sez6l2
F1LWT1	Cadps2
Q6AYD5	Gspt1
D3ZUN5	Pofut2
Q3MID4	Shpk
F1M6X5	Txnrd2
D3ZLA3	Cpne3
Q6QI09	Taf3
F1M853	Rrbp1
A0A0G2JSH4	Gsk3b
M0R6T4	Mms19
E9PU37	Zdhhc13
G3V826	Tkt
F8V328	Rab8a
A0A0G2KAY8	15-sep
A0A0G2JSS8	Prdx5
Q68FZ8	Pccb
B0BMT0	Acta2
G3V8P6	Zbtb7a
A0A140TAI1	Ubqln1
G3V803	Cdh2
A0A0G2K007	Stk39

G3V8E4	Ubfd1
A0A0G2JY26	Dnajc6
D4ACQ9	Gabpa
Q6IRL3	Rtn4
D3ZF39	Uap1
G3V619	Ascc1
G3V6U9	Setd3
B2RYR8	Rps8
B2GUZ7	Tbcc
F1LRM5	Bag1
B5DF62	Pak4
D4AB36	Flywch2
O89035	Slc25a10
Q5RK08	Gbas
Q6AYA7	Rfk
G3V7U4	Lmnb1
D3ZM33	LOC100362298
A0A0G2JTH6	Ehd3
A0A0G2K8S6	LOC103692171
D4A916	Celf3
Q6PCU4	Emd
F1LQE6	Ighmbp2
G3V6S5	Mthfd1
A0A0G2JW51	Dhx30
F1MAG0	Dfna5
F1LN75	09-sep
D3ZFY8	LOC100912618
Q6AZ33	Blvra
R9PXY7	Slc8a1
A0A0G2K6U1	Nsf
Q4KM87	Actl6a
B2RZ08	Tmem263
F1LRI5	Gcn1l1
Q498R0	Taf6
F1LX22	Pip4k2a
D3ZYQ9	Rnf20
D3ZE49	Trappc12
Q68G11	Csnk2b
D4A6X7	Ppcs
Q6PDV6	LOC100911847
A0A0G2JY82	Scamp1
A0A0G2JXG5	
D4AA38	Hlcs
F1LRX2	Fhod3
A0A0G2K5C0	Camsap3

D4AC23	Cct7
Q67ER1	Rnf113a2
F1LXF5	Get4
Q5PQU0	Kdr
Q6P9Y4	Slc25a4
A0A0G2K9M5	Tbc1d8b
A0A0H2UHD9	Ncln
A0A0U1RRQ3	Ubap2
A0A0H2UH90	Vwa5a
A0A0G2JUJ9	Sec63
A0A0G2JVS7	Ciao1
A0A0G2JSR4	Stat5b
A0A023IKI3	Psmb8
D4ACJ1	LOC100363469
B5DFC4	Prkca
D4AAV0	Irf6
D4A5S9	Prpf39
G3V9D7	Add3
M0RB75	Kdm4b
A0A0G2JSV3	LOC314140
B4DGJ5	PAF1
A0A0G2KAI1	Pde1c
B2RYN1	Fn3krp
A0A0G2K943	Ap2a2
G3V9J9	Mapk4
F2W8A6	Npy
F1M8X9	Gbf1
Q566D2	Tigar
D4A7D6	Bcl9
Q6P3V8	Eif4a1
B3DM93	Pdcd4
F1M446	AI314180
A0A0G2K9Q3	Naa25
A0A0G2JW12	C4a
D3ZW58	Casc4
D4AC99	Chtf18
A0A0G2K9D6	Smarcc1
E9PU23	Jag2
G3V6Y3	Kif12
A9CMB4	Ubxd2
M0RCH0	Eif3i
A0A0G2K8R3	Lmo7
F1LRI7	Aak1
D3ZUV9	Cnot3
B2RZ20	Ssna1

A0A0G2JSR9	Sdf4
A0A0G2K8P3	Nedd4
A0A0G2JSV6	Hba-a2
Q5EBC7	Rabep2
F1LPQ1	Nup98
D3ZJ29	Zfp512b
F1LVZ9	Hectd3
A0A0U1RVI2	Mapk10
A0A0G2K0F3	Epb41l1
A0A0G2K6C0	
F1LP76	Ikbkap
O08769	Cdkn1b
F1LM75	Rai14
G3V6G5	Tbl1x
F1LT94	Arhgef18
E9PTK4	Thnsl1
D3ZN63	Wdhd1
D3ZKR9	Zcchc6
R9PXV9	Ppme1
A0A0G2JZR4	Rab11b
A7BFV9	
Q7TP08	Amacr
B2RZ79	Iscu
A0A0G2JWK2	Mecp2
Q7TPJ3	Gusb
F1LMM9	Gfm1
A0A0G2JU82	Macf1
A0A0G2K5T1	Camsap1
M0RA26	LOC100362987
G3V7Q7	Iqgap1
F1M779	Cltc
D4A8L5	Mocs3
G3V7F1	Mlst8
D3ZDF0	Nptn
A0A0G2JUL7	11-sep
B0BMS9	Prkrip1
A0A0G2K4Y7	Myo5a
D3ZTP3	Tmem59
F1M1Y0	Sgip1
E9PU15	Dicer1
A0A0G2K7J8	
Q6AY58	Bcap31
B0BNJ4	Ethe1
A0A096MKC0	Gigyf2
D3ZRG7	Rbm26

Q6QI79	Cmtr1
D4A9J4	Whsc1
A7UAK3	Pfkfb3
A0A0G2JXI9	Hist1h2bo
A0A0G2JT93	Ctnnb1
A0A096MK29	Nt5c3b
B4F778	Rfc4
A0A0G2K6X6	Mark2
A0A0H2UHL9	Dbn1
D4AEH3	Psmd7
B0BNB5	Nup43
Q6P751	Cdk2
D4ABC7	Uprt
D3ZPP2	Arl8a
D3ZUW6	Ube2c
D3ZU83	Ergic3
A0A0G2JWH4	Agpat4
A0A0G2KB63	Phb2
Q45QL8	Gnb4
D3ZAZ0	Eif3m
Q6QI86	Hdhd2
F1M471	Epm2aip1
F1M8K8	Mbnl2
D4A8G0	Lsm12
D3ZZB0	Dip2c
F1LQS3	Rpl6-ps1
D3ZNQ6	Ube2m
Q5BJU0	Rras2
F1LUM5	Tubal3
B2RZ66	Srp19
D3ZCS4	Iqgap3
F1MAB2	5330417C22Rik
F1LPG2	Dock8
F1LNB2	Phactr1
M0RAR7	Cbx4
Q5PQP7	Gtf3c2
A0A0U1RRS9	Lamp1
Q7TP61	Acat2
D4A5A6	Polr2a
F1LQC8	Cdk7
A0A0A0MY49	Dnm2
A1L1J7	Pbk
D4A5R0	Ppih
F1M790	Ptgfrn
Q6P796	Spint2

B1H270	Suclg2
D4A3D9	Stk32c
R9PXU5	Raf1
F1MAJ8	Zfp91
D4ABP4	Rab3gap2
A0A0G2K970	Smad4
A0A0G2JUZ0	Mtmr1
F1LPQ8	Secisbp2
D3ZR52	Lpcat4
D3ZXJ5	Efl1
G3V8R7	Gatad2a
Q6P3V9	Rpl4
F1M392	Limch1
D4AAL2	Ncapg2
A0A0G2K7W5	Ahsa2
M0RDK9	Acad8
D3ZUQ2	Naa10
D4A6K4	Golga1
D3ZN59	RGD1559962
F1LQ83	Tmem9b
A0A0G2JY22	Dip2b
B2RYS8	Ndufb8
B2RZ64	Mbd3
A0A0G2K9W5	Maged1
A0A0G2JSL4	Tsc2
A0A0G2QC17	Pdp1
D4A031	Ddx42
D3ZK36	Uhrf2
D3ZU52	Cops7b
A0A0G2K4R9	Eogt
G3V888	Acly
V9GZ88	Inppl1
A0A0G2K7C4	Csnk1g1
A0A140TAF0	Tpm3
F1LRK1	Atp4a
D3ZQQ1	Aftph
F1M7Q7	Otud4
G3V6K1	Tcn2
D4ADD8	Dctn6
A0A096MIY6	
F1LR39	Wdr18
D3ZQ57	Plxnb2
A0A0G2K7S6	Wdr44
A0A0G2JU14	RGD1307947
A0A0H2UHP0	Ctcf

A0A0G2K4C4	Rps9
F1M8G8	Sipa1l2
Q5XI34	Ppp2r1a
D3ZAN3	Ganab
A0A140TAH3	Grwd1
A0A0G2JYA2	Arfip1
D3ZDI7	Ppp2r5a
A0A0G2K1C8	Hdac3
G3V8Q8	Sec23ip
D3Z8P5	RGD1566085
Q63489	Gtf2f2
D3ZZK0	Efcab7
Q4FZS1	Cbx8
F1M6D0	Mrps6
Q5BJN3	Tial1
D4A1D4	Nudt14
M0RAJ5	Prr14l
D3ZVE5	RGD1561662
Q6P9U5	Rpl9
F1LM55	Ccar2
B0BN51	Snrpb
G3V9M3	Wdr47
F7EUU4	Cops5
D3ZEG9	Ercc2
Q642B5	Armcx2
D3ZRN5	Trove2
D4A175	Trit1
A0A0G2K562	Adam10
F1LXV3	Stk26
Q496Z3	Ipo13
A0A0U1RRQ2	Arpc5
G3V7V2	Aamp
A0A0G2JYB1	Capzb
A0A0G2K3H5	Sin3a
A0A0G2JVY4	Tmem63a
Q6GQQ2	Pick1
A0A0G2JWJ7	Gorasp1
F1LM66	Eftud2
A0A0G2JZI2	Eprs
D3ZIT7	LOC103689975
D3ZUD8	Tm9sf3
D4A5L9	LOC679794
Q4FZR0	Vars
B5DF46	Pmm2
G3V631	Rabgef1

Q4KM60	Rpl10a
D3ZF13	Ndufab1
Q9JIK3	Ddx20
A0A0G2JVG3	Pkm
M0R3Y7	LOC100911660
Q5XIH3	Ndufv1
D3ZP27	Dym
D4A5I9	Myo6
F1LST4	Mapt
A0A0G2K0P2	Smad2
F1M7S2	Psmf1
A0A0G2JXZ3	Atrx
G3V6B8	Tsr2
D3ZG88	Sssca1
D3Z937	Hmg20a
A0A140TAG3	Zc3h18
D4A9Z8	Chmp4bl1
A0A0G2JTF2	Dab2ip
D4ADE5	Setd7
B1WC67	Slc25a24
A0A0G2K0P1	Kifap3
B2RYG2	Pck2
D4AA31	Prcp
A0A0G2K0J3	Ank3
A1L127	Rab3ip
D3Z9L5	Wdr11
G3V804	Sfxn3
G3V6G6	Sytl4
D4ACT4	Ankrd52
P97601	Hspe1
F1LPH3	Pam
C8CHS6	Sod2
D4A414	Cox15
D4A4L5	Isca2
G3V8Q1	Cope
A0A0G2K5I7	Kdm4a
B5DF50	Galnt2
A0A0G2K7G8	St7l
Q66H91	Git2
D3ZRM5	Rab23
A0A0G2K1V3	Tsen15
A0A0G2K5I8	Zfp62
F1M6C2	LOC103691939
Q6PDV9	Rps11
M0R757	LOC100360413

M0RCH6	
A0A0G2JUX4	Usp47
G3V9P7	Htt
D3ZH41	Ckap4
A0A0G2JZQ9	Krt77
A0A0H2UI27	10-sep
B2RYP5	Ppm1f
B5DFK6	Ap3d1
D3ZIV8	Ythdc2
D3ZEA0	Fndc3a
A0A0G2K3Q6	Aldoc
A0A0G2JXD7	Akt3
D3ZF26	Tnks1bp1
D3ZXP7	Arpc1a
D3ZYM6	Calr4
D3ZNF4	Tbl1xr1
G3V8Y5	Polr2b
D3ZN76	Sec16a
A0A0G2K6T6	
D3ZA31	Mtmr2
A0A0H2UHM9	Pja2
Q68FS8	Rtcd1
A0A0G2K0H9	RGD1564855
D3ZFA8	LOC100362366
F1M5N4	Me3
C7C5T2	Pfkp
D3ZFU9	Mylk
D3ZV40	Unk
Q6AYA2	Vrk1
A0A0G2JVH4	Immt
Q9QXG3	Stx1a
A0A0G2K808	Asap2
Q6TUH3	Usp40
P97886	UGT1
B5DEF3	Ggcx
Q5FVC4	Dnajb12
D4A8T3	Copz1
C7E1V1	Itpr3
A0A0H2UHB5	Sqstm1
G3V7M5	Pcyt1b
D3ZX42	Rabgap1
D4AE06	Fkbp15
D3ZES2	Trappc6b
E9PTG1	Smarca2
B3IYD2	Ufc1

D3XAM6	Map2k7
A0A0G2JZH8	Pdhx
Q8K5A0	Ptpn2
A0A0G2JTT4	Rab30
F1M8H2	Wars2
D4A022	Gapvd1
A0A0G2K4X5	Zhx3
D4AE96	Ipo7
A0A0G2K761	Cul2
G3V8S2	Shc1
A0A0G2JVG2	Vars
D3ZZS8	Atp6v1b1
Q9JMG8	Dpysl5
A0A0G2JZ01	Eya3
B5DEX4	Vasp
B2RYP6	Luc7l2
A0A0G2K8C1	Npnt
B0BNH1	Gnptg
Q5RKJ9	Rab10
D3ZN38	Stard5
A0A0G2K9F0	Kat7
F1LMT5	Pex5l
A0A0G2JXI7	Foxp4
B2RZ76	Dhrsx
D3ZJD3	RGD1565183
F1M8H6	Pip5k1c
F1LR60	Apba1
F1LYJ8	Gramd4
G3V6R7	Oxsm
G3V9W0	Cetn2
D4A510	Smarcc2
D3ZQG8	Kif2c
B5DEP4	Mrpl42
D3ZY02	Athl1
A0A0G2JXT3	Fdps
D3ZQ63	Mettl2b
F1LV89	Rap1gap
F1M5A9	
D3ZM69	Epb41l2
G3V8Y8	Hip1
B0BNB2	Denr
A0A0G2K305	Cwf19l2
F1M7V4	Pclo
D3ZX61	Wnk1
G3V8C0	Dctn5

B2RYW7	Srp14
A0A0G2K2B5	Asph
A0A0H2UI05	Cfap20
A0A0G2K5N6	Rock2
F7FLB2	Pgm2
A0A0G2K8N9	Nek9
A0A140TAJ3	Fubp1
B2GV92	Ptges3
A0A0G2K737	Txnl1
D3ZT98	Bola3
G3V660	Nsun5
B4F7A5	Cd99
A0A0G2JST3	Krt1
Q5XIA6	Smpd1
D3ZN39	Usp8
A0A0G2JSZ9	Ccs
Q6P6U2	Psmc3
A2VCV8	Mlx
D4A2H2	Sptlc1
D3ZIV3	Mad1l1
A0A096MJZ0	Dnal1
D4A8C5	Plcb4
B0BN39	Med18
G3XEV5	Wac
Q9QYV2	Enpp3
A0A0G2K110	Eif6
F1LSS1	Smc1a
F7FHF7	Mfn2
Q6P9U0	Serpinb6
F1LM69	Ddost
D4A401	Tex10
Q6IRJ7	Anxa7
Q7TP82	Man2a1
A0A0G2JTG7	Hnrnph1
F1LRK9	Ppp4r1
D4A0U9	Msh6
A0A0G2K5S7	Entpd3
D4A8Y5	Snd1
G3V790	Smarca4
D3ZSW5	Txnl4a
G3V9M8	LOC100910130
G3V9U2	Acaa2
D3ZDI9	Usp13
M0RD40	Sik3
B1H262	Ppp1r9b

R4GNK3	Txn1
F1LNL2	Smarca5
A0A0G2JT51	Pafah2
A0A0G2K677	Sptbn4
A0A096P6L1	Snrpc
D3ZA85	Nfu1
A0A0G2K3V4	Ogt
M3ZCQ2	Snrnp200
Q0ZFS8	Srsf3
F1M0Y6	Dach1
D3ZD97	Dhx15
A0A0G2JYA4	LOC100362453
A0A0G2K7F5	Dlg4
Q6P9V5	Psma2
D3ZTF6	Pik3c2a
A0A0H2UHW4	Pcnp
A0A0G2JUV8	Camk2a
F1LMQ3	Psmd8
A0A0G2K2C2	Gmps
D4A7Z2	LOC100360654
B0BMY6	Otulin
D3ZXE2	Pds5b
A0A0G2JVH5	Hltf
A0A0G2JUM2	LOC361635
D3ZIE9	Aldh18a1
F1M4B6	RGD1563349
Q6UN82	Setsip
D3ZAS8	Sart3
Q5U2S7	Psmd3
A0A0G2JT62	Ptprf
G3V7T6	Sf3b1
B0BN63	Ahsa1
D4A3T0	Sos1
D3Z863	Cwf19l1
A0A0G2K1G8	Slc7a14
A1A5L1	Blmh
Q6P7A7	Rpn1
F1LPM4	Maob
D3ZAQ4	Psmg2
B2RYI6	Snx4
G3V6U3	Alg2
E9PT51	Poldip2
R9PXW7	Stip1
Q6IN14	Cd82
D3ZDC2	Slc43a2

Q56R17	Kpna4
A0A096MJW8	Plch1
F1M801	Anapc1
D3ZD09	Cox6b1
D4A9L2	Srsf1
M0R9R0	Gprasp1
A0A0G2K9U6	Atg16l1
D3ZV52	Itsn1
A0A0G2K717	Cnot2
A0A0G2K7M1	
Q45QK6	Gng2
A0A096MIY3	Tdg
F1M2S2	Dnajc24
F1SW39	Psip1
Q56A29	Vsnl1
G3V662	Nup153
D4A7I6	RGD1309995
D3ZGQ8	Rsf1
D4A3P1	Ubqln4
A0A0G2JT30	Mat2b
Z4YNF4	Acp1
Q6P9X1	Clns1a
P70565	Jup
A0A0G2JTG1	
Q3MHT9	Fam129b
D3ZZL3	Naglu
D3ZI07	Kif3b
A0A0G2K9P5	Cog5
Q2TA67	Mcmbp
D4ABC4	Ppp4r3a
F1M388	Auts2
A0A0G2K0B0	Cdv3
Q4QQS6	Alg5
A0A0G2JXH2	Ccdc51
B1WBR0	Smad5
A0A0G2JXR7	Cwc27
D3ZPJ9	Ascc2
G3V7A6	Ccdc174
A0A0G2K165	Ppp2r2b
A0A0G2JSR7	Matr3
D3ZUJ8	Tmtc3
Q7TQN4	Rela
A0A0G2JXD9	Dopey2
D4AAH6	Psmg1
D3ZWJ9	Fam234b

A0A0G2JSZ5	Pdia6
Q5XI21	Tom1
A0A0G2KAH7	Ext2
G3V644	Ndufv3
B0BMW2	Hsd17b10
Q4VFZ4	Katnb1
G3V6P8	Gng12
Q68FT0	Arrdc1
D4A2Z8	Dhx36
Q8VHJ9	Syne1
Q4V8I9	Ugp2
D4A648	Stk4
Q4G052	Wnt4
B2RYJ2	Dbr1
M0R8E8	Ipo11
F1LMK3	Sdccag3
A0A0G2QC02	Skiv2l
Q5U1W8	LOC100911295
D3ZC46	Tcf25
D3ZY71	LOC100910137
M0RC73	Naa16
A0A0G2JTA6	Igf2
Q6S3A0	Plec
D4AEH9	Agl
Q5U2N2	Usp14
A0A0H2UH97	Anapc5
D3ZSP7	Ttc3
A0A0G2KA12	Kif1b
E9PTA5	Pign
F7EPZ4	Dis3
A0A0G2K1L8	Basp1
B0BN55	Urod
D3ZEH3	Sec61a2
F1LZH9	Ankrd44
B3SVE9	Tecr
A0A0G2K490	Tnik
A0A0G2JXF9	Hcfc2
G3V9N1	Pgam5
Q642B0	Gpc4
Q64608	Atp1a1
Q6IMZ5	Tmod1
G3V715	Prkaa2
A0A0A0MY22	Siae
F1M6X7	Arhgap17
D4A702	Synpo2

A0A0G2K249	Prpf4b
D4A5S0	Rock1
D4A2P1	Ccar1
A0A140UHY1	Ttc4
D3ZCG4	Spag7
M0R8V0	LOC100910056
F1LQ27	Fam98c
A0A0G2JWJ0	Ppp1r12a
D3ZDU2	Rptor
F1LNV7	Gtf3c1
A0A0G2JV65	Ywhaz
A0A0G2K2V6	Krt10
A0A0G2K0I3	Nampt
D3Z981	Plxna1
D3ZDP9	Zbtb11
F1LTZ5	Enpp4
Q7TP03	Glt8d1
A0A0H2UHI7	Atad1
M0R7A6	Itsn2
O70371	
A0A0G2K714	Sh3glb1
D4A7V1	Sh3glb2
E9PSJ1	St3gal1
Q99NI1	Nme3
A0A0G2JTI7	Prpf3
A0A0G2JVB6	Stx12
A0A0G2K889	Ehmt1
D3ZT01	Cog2
A0A0G2K4H3	
D4AED6	Wbscr27
D4A531	Polr2i
B4F7F3	Arvcf
D4A626	Clmn
F1LSC3	Sf1
D4A994	Emc1
A0A0G2KB58	Cdc42bpb
Q3MHS7	Gmds
Q5I0F0	Drg1
D3Z949	RGD1561671
D3ZD80	Gtf3c4
A0A0G2JWJ4	LOC100911515
A0A0H2UI20	Ctbp1
Q6P9X7	Txndc9
D3ZTA8	Rbm33
E9PT53	Wfs1

Q7TP16	Tctn2
A0A0H2UHM7	LOC100909441
A0A0G2K664	Hspb11
A0A0G2K8M2	Rsph1
A0A0G2JUP0	Nek7
Q5U2U8	Bag3
G3V874	Epb41l3
B1WC70	Ppp1r8
Q5RJK9	Polr1c
G3V7J7	Eif5a2
D3ZAS2	Sgsm1
D4A5Q2	Smc2
Q9JIJ7	Ddx1
B2RZ72	Arpc4
G3V6L9	Fkbp3
Q6PCT9	Psmd6
F1M0Z1	Trio
D4A3R6	Zmym2
D3ZBZ9	Ttc33
F1M863	Ppfia4
A0A0G2JSJ8	Fuca1
F1LNF3	Nfxl1
G3V9K0	Cars
F1M9C9	Hars2
A0A0G2K6W6	Dis3l2
M0RCT5	Eml1
D3Z9C0	Mios
D3ZC89	Fam114a2
Q71VC8	Cdk4
Q5U3Z5	Bri3bp
Q6TXG7	Shmt1
D4A4S3	Hspa8
F1LPE9	Erc1
Q7M094	
D3ZQR6	Eml6
A0A0G2K273	LOC100909481
Q4V8G6	Mettl3
Q6AY24	Ubl7
D3ZNA3	
Q91Y79	Cep170
F1LNG7	Gna13
B0BMW4	Gnas
B2GV74	Klc2
A0A0G2K613	
F7FG68	Syvn1

F7EZ89	Tbc1d15
A1L122	Nae1
Q5PQK2	Fus
Q7TP42	Sec62
B0BNJ5	Edf1
Q5U2P5	C2cd2l
D3ZKG7	Inpp5f
F1LRV4	Hspa4
G3V7G8	Gars
D3ZYM7	LOC102550385
D4A7F2	Mycbp
F1LN29	Stau2
M0RC54	Bod1l1
F8WG91	Arl3
B2RYP3	Fam91a1
G3V7T3	Pank4
F1M2K4	Lats1
H2BF30	Fads1
G3V9H0	Rasa1
D4A6D9	Hs1bp3
D4A7U5	Vwa5b2
B5DF01	Vps11
D3ZXM2	Rad9a
A0A0G2K7X2	Traf3
G3V802	Ccna2
G3V8Z3	Gpkow
B5DFI9	Pdk3
A0A0U1RRY3	Eif2b1
A0A0G2K0D7	Psma6
D3ZZK1	LOC100359563
Q498N3	Dctn4
D4A4D5	LOC498555
A0A0G2K5T3	LOC685590
A0A0G2JT90	Ash2l
F7FKF2	LOC364556
D4ACV0	Nelfb
Q2I6B4	Atp6v0a1
B4F7E2	Arsb
Q6AZ05	Ica1
F1LP21	Timm8a1
G3V8A5	Vps35
A0A096MJZ2	Tbl2
A0A0G2JU09	Ep400
Q568Y6	Dmap1
D4A853	Cic

F1M4I4	Vps51
F7F350	Syap1
D3ZZZ9	Ctnnd1
D3ZRK0	Fam83h
B0BNG5	Hif1an
G3V711	Adam17
D3ZEM8	Tsr1
A0A0G2K0J7	Ctdp1
D4ADS9	Efr3a
B5DEP7	Snrpg
Q66HF7	Pja1
D3ZWJ1	Dnlz
A0A0G2K911	Nsfl1c
B2GUZ6	Rtn4ip1
D3ZMW3	Msto1
D3ZR64	Zfp598
B6DYQ8	Gstt1
D3ZQV8	Phf6
I6L9G6	Tardbp
Q1RP74	LOC103690005
A0A0G2K8G0	Apc
B2RZ22	Paip1
D3ZY39	Rrp1b
D3ZCM7	Tle1
A0A0G2K527	Git1
Q6AZ40	Prpsap2
D3ZXH7	Alyref
Q3B8N9	Bphl
Q5M893	Hmbs
A0A0G2K7Y0	Cr1l
G3V6K0	Commd5
D4A1J6	Ankfy1
G3V6H5	Slc25a11
Q5U328	Ncl
F1M265	Palld
R9PXR4	Tomm70
D4AC65	Coa7
D3ZKE0	Gnptab
B1WC32	Uba2
B0BN97	Txndc12
A0A0G2JWH3	Ncapd2
F1LN34	Ankh
Q6PDW4	Psmb1
D3ZHF8	Guf1
F7F0B1	Zc3hav1

Q3KRF0	Vps33a
F1M8A4	Ppfia2
M0R5Q9	Gde1
Q569C9	Golph3
M0R4P2	Tsfm
G3V7B5	Prpsap1
Q6IMZ3	Anxa6
Q0QF18	Sdha
D3ZYQ8	Uckl1
D4ACA3	Dscc1
A0A0A0MY09	Hsp90b1
D3ZGX8	Brd4
B2RYW3	Ndufb9
G3V699	Sec31a
F1LVX1	Dnajc1
A0A096MK61	Crtap
F1LZJ4	Hyi
D4ABI6	Uchl3
F1LQ05	Sh3gl2
D4A8X8	Cttnbp2nl
F1M6V8	Strn4
D3ZE20	RGD1560225
Q3S4A4	Arfgap1
F1LMB9	Exoc2
D3ZEJ9	RGD1310429
A0A0G2K700	Dkc1
A4GW50	Stk38l
D4A914	Xrn2
O88321	Psmd4
D3ZSC2	Pdcd2l
D4A5X7	Gdap1
D3ZGT6	P4ha2
G3V8V1	Grn
A0A096MJI9	Atp1b1
D3ZB65	Bccip
Q4V8E2	Psmd14
F1M6G3	Gm8444
G3V792	Dync1i1
Q6IRF8	Atp6ap1
D3ZP47	Phpt1
Q6P9V6	Psma5
F1LP82	Rab2a
A0JN17	Kras
D3ZPA1	Gcc1
B0BMV8	Dhfr

M0R5F8	Hmgn5
Q7TP34	Mrfap1
A0A0G2K4U4	Cacnb1
Q6PED0	Rps27a
M0R462	Mllt1
A0A0G2JSP3	Cul3
D3ZSP1	LOC100361838
Q6P736	Ptbp1
B2GUV5	Atp6v1g1
F1M4A4	Kif1a
A0A0G2JY73	Eif4g3
Q2I6B1	Atp6v0a2
A0A0U1RS33	Sgsm2
F1LQZ8	Dhtkd1
A0A0G2JZS2	Pabpc1
F1M742	Fnbp1l
A0A0G2K1K5	Trappc11
Q6AY81	Npdc1
F1LR12	Nav2
G3V9Q4	Stk38
Q5BJX6	Vps50
Q6MG13	Dhx16
D3Z8L5	Pum1
B2RZ62	Ap3s1
Q5BJP4	Rbm39
A0A0G2K121	Mlec
F7F707	Nrn1l
D3ZAS9	Ddrgk1
A0A0G2K1G0	Bclaf1
A0A0G2K583	Mtap
A0A0H2UI10	Mgea5
Q6P3E1	Rps16
D3ZQI0	Polr2j
A0A0G2K724	Mcts1
E9PU09	Akap12
B5DFG9	Pik3ap1
F1MAK3	Arhgap32
B5DER3	Iah1
D3ZPE5	Vps53
D3ZCT7	Sec23b
B1WBW9	Abhd10
D3ZDU5	Pfn2
F7F189	Ndc80
A0A0G2K4T8	Mipep
G3V7I8	Slk

M0R4P9	Ube2h
Q5PQK5	Rdx
M0RCV0	LOC100910575
D4A1Y5	Mthfd2
B3DMA5	Cnot7
D3Z9D2	Fyco1
A0A0G2K1S6	Me1
D4A415	Fam175b
A9UMW0	Ubl5
D3ZYD7	Ccdc88a
D3ZBH5	Lmtk2
A0A096MK59	Bud31
B2GUY4	Dmtn
D3ZFB2	Luc7l3
Q7TPJ1	Igtp
A0A0G2KA38	Isg20
F1LN07	Scgn
Q2YDU5	Fgd1
A0A0G2K6X9	Adk
D3ZQK4	Txndc16
B2GUV2	Vps52
Q4KLH7	Rad21
Q6IE71	Usp11
A0A0G2K5C6	Map1a
H1UBM7	Cpne2
M0R9L0	Naca
A0A0G2JZA1	Robo2
D4ABI7	Hacd3
D3ZQ26	Rnf25
B2RZ74	Snrnp70
A9CMB7	Dars
G3V6W6	Psmc6
G3V607	Chm
D4A601	Taco1
Q6IN37	Gm2a
A0A0A0MXV9	Chst11
G3V6N4	LOC100912042
D3ZUX5	Chchd3
A0A0G2JWA5	Slmap
Q642D2	Acp2
A0A0G2K1D2	Rap1gds1
D4A7Q9	Gtf3c3
G3V6P6	Rbm3
Q66HK4	Tceb3
A8USN8	

A0A0G2K849	Taf4a
Q5BKC3	Park7
A0A0H2UHC0	Arhgap44
A0A0H2UI38	LOC100910336
D3ZIK8	Fbxo38
F1M110	Nmt2
G3V7B6	Ptpmt1
D4AC38	Ago1
A0A0H2UHP9	Rab6a
B2RYT7	Hdhd3
A0A0G2JWH5	Stt3a
B5DF86	Cog8
G3V6S3	Calu
G3V6Y6	Pygb
D3ZSA9	Nomo1
D4A0E8	Prmt5
A0A0G2JXA1	Rab3gap1
Q7TP77	Mrpl49
Q4QQV4	Hars
G3V9M1	Ddx23
G3V9D3	Sel1l
B1H249	Gnpnat1
A0A0G2KAI8	Drg2
F1LPA4	Pkn2
F2Z3T0	Cdc73
B2RZB5	Chmp2a
D3ZWT8	Atp5h
A0A0A0MXW1	Bckdhb
F1LWB9	Ankrd50
M0R9N8	Anlnl1
M0R9Z5	Irf2bp2
G3V7Y3	Atp5d
D4A769	Samd4b
D4ABY4	Ube2j1
B4F765	Ift27
F1LY69	Ift140
D3ZTM7	Efcab14
F1M298	Fbxo18
Q9QYU8	Gcsh
M0R5Y9	Zbtb8os
A0A0G2JWX1	Psmd11
Q66H61	Qars
Q5FVF3	Gga1
B2RYW4	Mrpl53
A0A0U1RRP2	NEWGENE_1582994

A0A0G2K4G3	Nedd8
Q66H09	Ttc1
G3V6I9	Rpl26
R9PXS2	Ocrl
Q32PX6	Rhog
G3V7Q6	Psmb5
Q499T5	Capn7
B0BMT9	Sqrdl
D4A6X4	Acyp1
D4A069	Zmym4
M0R6Y8	
D3ZWG9	Ptar1
A0A0G2K9Q8	Ubr7
D3ZUD3	Wipf2
Q56R18	Kpna3
A0A0G2JVD6	Kmt2d
B1H227	Rcc1
D3ZV63	RGD1307830
M0RBD1	RGD1560821
F1LM09	Usp7
D3ZWA8	Appl1
Q5FVC5	Nectin2
B2RYP4	Snx2
Q6U1J1	Tbc1d22b
D4A1W5	Rbm4
D3ZQL8	Pum2
B4F7B2	Taf10
F1MAF2	Akap17a
M0R5J4	
Z4YNP1	Celf2
A0A0G2K4U2	Gstm7
M0R750	Srcap
A0A0G2KA91	Mier1
F1M3G7	Akap13
F1LQH9	Bag2
F2W8B0	Comt
D3ZQQ5	Dnm1
D3ZZH6	Map3k5
B2RYU7	Cbx5
A0A0G2K8K9	Rbmxl1b
Q5U3Y8	Btf3
F1LYG2	Sipa1l3
F1M9Q3	Ltn1
M0RC65	Cfl2
D3ZRL3	Dusp23

A0A0G2JX05	Uck2
D3ZPL1	Cpsf6
M0R7Z0	
A0A0G2JWT6	Pex16
Q6IRG9	Ap3m1
Q9JJI7	St14
F1LSY2	Nptxr
Q45QL6	Gnb2
A0A096MJP9	Ddi2
B0K030	Dnajb1
B0BN99	Hmgb3
E9PTX9	Slc12a2
F1LRE5	Osbpl9
F1M0F4	Exoc6
F1LPT8	Scaf8
A0A0G2K427	Ivns1abp
D3Z9M1	Mettl16
Q5M9F6	Mthfs
A0A0G2JWD6	Ap3b1
F1LNC3	Phlpp1
A0A0G2K6P1	Hsd17b8
D3ZD72	Ncapg
Q4V8H5	Dnpep
D4A8H8	Cyfip1
Q5FVM2	Psme3
F1LNC5	Rims2
G3V9W5	Pip4k2c
Q6AY02	Rbm17
D3ZK73	Cul4b
A0A096MKB0	Rab24
A0A0G2K5D7	Specc1
B4F768	Aldh4a1
M0RCC8	Msra
D4AE00	Ap3b2
A0A0G2K8E7	Gdpd1
Q5M9H7	Dnaja2
F7EVX2	Eif2b2
F1LRZ9	Rundc3a
D3ZZR9	Fkbp2
A0A140UHY3	Ewsr1
E9PTB3	Mtg1
F8WG67	Acot7
A0A0G2JX67	Prepl
M0RAI9	Nelfcd
G3V8T7	Tdrkh

B2GUZ3	Mthfd1l
A0A0G2JSQ6	Smad1
D4A9Z6	Mrps35
Q3MHS9	Cct6a
Q5RKG9	Eif4b
B0BMU0	Ppie
Q5VLR5	Erp44
G3V8D0	St8sia3
Q66HL0	Nt5e
G3V8G5	Glg1
A0A0G2K777	Rhot1
D3ZKE1	Uvrag
D4A857	Ipo9
B2RYA6	Prcc
E9PST5	Acin1
D3ZR27	Mfn1
G3V6H0	LOC100363782
F7FJQ3	Npc2
A0A0G2K7K2	Aifm1
D3Z837	Cdc42bpg
D3ZUT8	Ndrg4
A8IRI3	gr
Q4V8Q2	Spint1
A0A0G2JWA1	Gnas
G3V7K5	Npc1
B0BN65	Fam49a
A1L1L5	Ccnk
Q6TUE3	Tmem106b
D4A1G8	Cep170b
A0A0G2JW03	Lpin1
A0A0G2QC15	Htatip2
D4A6W6	RGD1561333
D3Z890	Daglb
D3ZZK8	Frg1
Q0VJ96	Rxra
D3ZXC7	
M0RDY1	Zfp74
Q5BK33	LOC652956
Q7M0B2	Ppif
B1WC51	Cks2
A0A0G2JTH4	Cd47
A0A0G2K793	Hsp90ab1
D3ZME7	Hscb
F1LM60	Arap1
Q7TP44	Srxn1

F1LQU9	Sgsm3
M0RAP5	Sbf1
A0A0G2K2Q2	Gcat
A0A0G2QC22	RGD1306215
A0A096MJY6	Gbe1
A0A0G2K598	Tacc2
B2RYK3	Spr
D3ZL83	Fhdc1
A0A0G2K502	Me2
Q6P9V1	Cd81
G3V7Q4	Ptpn12
M0RBF8	Exoc4
F1LPF6	Atp2a2
F1LNM0	Dlg1
D3ZL85	Hccs
R9PXT9	Prkar2b
G3V845	Plcg1
G3V912	Tmx4
D3ZMN5	Snx29
D3ZB48	Hook1
Q3B7U1	Maged2
D4A5W8	Pgs1
D4A0A1	Soga3
A1L1M0	Prkaca
F1MAD9	Smc4
D4A3V3	Parp12
G3V721	Wbp2
Q6AYP5	Cadm1
A0A0G2K2B6	Tpmt
A0A096MIS3	Eif2b4
D3ZGY2	Otud6b
D4A197	Mcee
G3V9T7	Asna1
A0A0G2K8M7	Tpd52l1
A0A0G2JZ92	Srgap2
A0A0G2JVA7	Eif1a
D3ZUB7	Anapc4
B0K008	Eif1
D3ZAQ1	Actr5
Q45QL2	Gnb4
M0R565	Wdr82
D4ACG2	Ilvbl
G3V907	LOC100911356
G3V959	Trafd1
D3ZMY8	Pcnt

D4A0C3	Hid1
Q4JFL8	LAR-PTP2
B0BNJ1	Sri
D4ADZ9	Pus7
F1LLX6	Cadps
D4AE68	Gnaq
Q7TP14	Chid1
B5DFC3	Sec23a
A0A0G2JU56	Mark3
A0A0G2K1P0	Ppia
B5DFB2	Rbbp4
F1M8V2	Ube4b
F1LSQ0	Faf1
A0A0H2UHA0	Ppp1r2
D4AEG3	Ppil4
B2GV73	Arpc3
D3ZU51	Rpp30
F1LSM5	Nckap1
A0A0G2K5G9	Ik
A0A0G2JUE4	Golga4
D3ZJX5	Timm50
F1LQM9	Xpo7
F1M642	Cnot6l
A0A0G2JZ04	Zcchc11
A0A0G2JSL0	rCG_63409
A0A0G2K0U8	Pus10
M0R7V3	Apoo
A0A0H2UHA6	Tmem33
F1LUW9	Tubg2
D3ZPP0	Zfp668
A0A0G2K4X8	Skp1
B5DEN5	Eef1b2
F1LRY5	Sardh
G3V617	Mapk14
D4A4H5	Sdf2
A0A0G2JXC3	Rps21
X2G6B3	Gck
D3ZD23	Abce1
Q6TUD1	Nars
A0A0G2K3Z9	
Q6MGC3	Wdr46
B5DEG6	Gart
D4A1U8	RGD1311744
F1M8D4	Bend7
I2FHN3	Ctsl

A0A0G2JTL5	Pc
Q68FQ9	Lancl2
B2RYQ2	Ppp2r4
B1WC71	Aspscr1
F1LNE7	Mark1
A0A096MJF7	Phka1
A0A0A0MY43	Ascc3
D3ZMN2	RGD1311747
A0A0G2K0W9	Psma7
D3ZXI0	Pycr1
G3V818	Parva
D3ZXP3	H2afx
A0A0G2K7T5	Ppp3cb
A0A0G2K865	Tpd52
Q6P9V7	Psme1
F1M8L1	Kif2a
A0A0G2JWB6	Pxdn
A0A0G2JX25	Gmpr2
A0A0H2UHK0	Sfr1
A0A0G2JX54	
Q6TUD3	Rdh11
A0A0G2K2I6	Dock9
A0A0G2K0Y9	Dlg3
D4A261	Ctc1
D4A264	Zadh2
B2GV99	Myl6
A0A0G2JXS8	Ssr1
A0A0G2KB55	LOC102553386
M0R402	Tmx3
D4A8A0	Cad
A0A0G2QC13	Zfp709l1
D4A0G9	Ercc6l
Q6P685	Eif2s2
F1LMW7	Marcks
A0A0G2K9C5	Numbl
G3V6L4	Kif5c
M0RA17	Snx5
Q6AYZ4	Ppp1cc
B1WC73	Arl6
A0A096MJZ7	Synrg
B5DEI0	Pcyox1l
A0A0G2JZ76	Synrg
D3ZLT7	RGD1305350
G3V9S2	Ammecr1l
D3ZQ80	Smg8

B1H2A6	Fxr2
M0R623	Gtpbp4
C0KUC6	Lims1
F7EL36	Anp32a
D3ZHC4	Hebp2
Q66HH8	Anxa5
D3ZW15	Sec24b
D3ZMS1	Sf3b2
Q3MIE3	Wash2
F1LN59	Eif4g2
D4A720	Srsf7
A0A0G2K7T6	Nup155
F1LNZ2	Numb
B2RZ33	Nck1
Q64599	LOC286987
D4A7R0	Srp72
G3V9W2	Jak1
A0A0G2KA27	Madd
B4F758	Hmgb1
F1M943	Armc8
A0A0G2JXN8	Osbpl8
Q4V884	Cdc16
D4A830	Ppa2
B6DYQ5	
A0A0G2K7E5	Cacna2d1
Q66H11	RGD1306195
A0A0G2K013	Actn4
Q5XIM7	Kars
B1WBY1	Cul1
F1M0U5	Nbas
F1LPM3	Sorbs2
D3ZG85	Cdkl5
A0A0G2K1U9	Irgq
D4A1L2	Nbeal2
D3ZZR5	Snrpa1
E9PT82	Strn3
D4A9A3	Cenpv
A0A0G2KB73	Rif1
A0A0H2UHC8	Ublcp1
D4A4P4	Flad1
G3V637	Stxbp2
D3ZLD5	Golga3
D3ZKP9	Cacna2d1
F1LVV3	Ranbp9
B0BN19	Rab22a

A0A140TAG9	Cdc37
A0A0G2JU91	Ncor2
M0RC57	Smap1
F1M400	Ankrd28
D3ZA66	Pstpip2
D3Z994	Tbc1d22a
A0A140TAA0	Usp28
Q4PLF2	CAT2
D3ZT03	Upf2
M0RDF1	Kdm3b
A4D0F6	
D3ZHD7	Rfx5
O70420	
F1M7S0	Magi3
F1M6W2	Ermp1
A0A0G2K6H2	Gstz1
A0A0G2K213	Ten1
A0A0G2K751	Dnajc8
D3ZVK7	Hist1h2ak
G3V8L3	Lmna
D3ZUV3	Eif2a
Q9Z0U8	Hnrnpab
B5DEJ5	Eefsec
D4A269	
F8WFH8	Wars
A0A0H2UHD8	Sh3kbp1
D3ZHQ1	Dpp8
Q4V8E1	Gatad2b
F1LWS7	LOC102546572
D4ACM2	Mad2l1
G3V9P0	Psmd9
Q6MG85	Agpat1
D3ZVW3	Zc3h4
Q3KRE2	Mettl7a
B2RYF6	Clptm1
D3ZRD3	Pde6d
A0A0G2K191	Tbce
D4ACZ5	Ndrg3
A0A0G2K2P5	Tjp1
D4AB33	Peg3
F1M1B3	RGD1564420
D3ZPV8	Ggct
D3ZUM4	Glb1
D3ZZ99	Add1
D4A1B2	Arpin

F1LV37	Tnrc6b
A0A0A0MXT5	Mina
Q6P9U9	Impdh2
D3ZMR2	Uhrf1bp1
D4A786	LOC103693430
B5DEH4	Uap1l1
Q156J1	
A0A0G2K0I1	Spata5
G3V945	Aldh5a1
F1LRH1	Msi1
D3ZL50	Ttc37
D3ZIC4	Ppp1r12b
Q9WUW7	Xpo1
B2GV69	Hnrnpa2b1
A0A0G2JTS3	Vps29
D4A8M4	Lrch3
G3V897	Ctr9
A0A140TAD1	Nudt5
A0A0G2KBB9	Ank2
B0BN83	Armc1
A0A0G2K0H6	Wdr13
D3ZLC1	Lmnb2
Q5U302	Ctnna1
Q6MGB4	Slc39a7
D4A1B9	Smc3
A0A0F7RQL3	Mif
E9PTV0	Guk1
B1WC26	Nans
A0A0G2JU66	Nploc4
B2RZ38	Rragd
D3ZHP8	Rpl5l1
A0A0G2K548	Akap9
A0A0G2K1Z2	Arhgap12
A0A0G2K9M7	Sgf29
Q6IE67	Psma3
B0K014	Dtd1
A9UMW1	Gsta4
G3V8D6	Trim3
B2GV01	Mta2
A0A0G2JUF6	Idh2
A0A0G2JTM7	Brd7
G3V7H2	Minpp1
M0R5N4	Pfdn4
A0A0H2UHQ0	Slc3a2
Q4KLZ3	Dazap1

A0A0G2K4T3	Acap2
D3ZD11	Spcs2
Q8CGQ3	Hras
M0RCX0	Pcbd2
D3ZT71	Bcl2l13
A0A0G2K3S6	Rbm10
D4A067	RGD1564541
A0A0G2K916	Ankhd1
A0A0G2K9B4	Mrpl15
A0A0G2JXC1	Lman1
F1LR10	Lima1
Q05759	Prkacb
D4A5T1	Sf3b5
D4ACD3	Usp25
A0A0G2JWR2	Pacsin1
D4AA59	RGD1564243
A0A0G2K161	Epb4.1
A0A096MK83	Bud31
D3ZXM4	Evi5l
G3V798	Srsf4
A0A0G2JZV8	Fmr1
Q5U362	Anxa4
M0R8B6	Tubb1
A0A0G2JWU1	LOC500959
Q4V794	Vps37a
A0A0A0MY14	LOC100359503
A0A0G2K6E0	Rhoc
A0A0G2K4M8	Acot3
A0A140UHX4	Prkag2
D3ZUM2	Sarm1
A0A0G2JXZ9	Ptprj
A0A0G2K9R0	Zswim8
D3ZVT1	Actr8
F1M9C3	Braf
A0A0G2K3T3	Clip1
G3V6C9	Cdc42bpa
A0A0G2K7B7	Cnot10
F1LNF1	Hnrnpa2b1
A0A0G2JSJ4	Syt13
G3V762	Tsta3
D3ZLF0	Gtpbp6
Q6AY18	Sar1a
Q6P9Y2	Rabggtb
D4A4J0	Supt16h
A0A0U1RRU5	Tab1

A0A0G2K0W4	Leng8
D3ZM09	Sars2
Q5RJK5	Cbx3
D3Z955	Pgm2l1
A0A0G2QC38	Srsf11
D3ZJ86	Slc9a6
F1LRL4	Tbc1d9b
A0A140UHX3	Ncapd3
D3ZC63	Cmpk2
D3ZHU8	Rbm19
D4IGX4	Fut8
B2RYM6	Zc3hc1
A0A0G2JYG5	Myo9b
M0RCP9	Pin4
B4DJE1	
A0A0G2K7Q6	Ak1
D3ZUI1	Apip
B5DFK1	Copa
B1H248	Vps36
F1LSE6	Ppfia3
Q6IMX8	Acot2
Q6LCQ4	Nme1
A0A0G2K824	Gmppa
A0A0G2JUN8	Psmb7
D3ZIU2	Psmg3
B5DF98	Map3k3
A0A0G2KAW4	Thop1
Q6PDW1	Rps12
Q9JHT3	Hacl1
A0A0G2K5H7	Sbno1
D4A7D3	Tbk1
B5DFM8	Bcas2
M0R735	Syncrip
M0R416	Pdxp
E9PT90	Spg20
Q6IRL1	Gas6
D3ZLM5	Nhlrc2
A0A0G2JSW0	Myl12b
A0A0G2K0P5	Trip4
D3ZGL0	Gripap1
G3V9Z3	Maoa
B2GVB4	09-sep
Q5U2Z4	Nfkb2
A0JN25	Mapt
B1WBY8	Hdac2

B2RYD7	Stt3b
F1LMM8	Pdk2
A0A0G2K756	Acbd3
B2GV82	Nle1
D4A4Z0	Ccdc12
D3ZCD4	Pitpnb
Q7M079	Hsp90b1
D3ZCN9	RGD1560073
Q5M9H2	Acadvl
Q5U2R9	Scfd2
B2RZ97	Kcmf1
Q6MG70	Neu1
D3ZTW1	Ints5
Q4QR73	Dnaja4
D4A6V3	
A0A0G2K8T0	Asah1
A0A0G2JX74	Mtor
A0JN29	Lnp
Q5M943	Thumpd1
A0A0H2UHT9	Cmss1
A0A0G2K9E0	Rnh1
A0A0G2JZC6	Arhgef11
A0A140UHX0	Prkcd
B0BNE9	Mtif3
A0A0G2K953	Vimp
M0R6L8	Dnajc19
Q8K1G4	Eef2
F1LVA9	Dock5
F1LNC4	Vti1b
A0A0G2JTB2	Gbe1
D3ZUL8	Zcchc8
B2RYJ1	Anapc2
F1M6V6	Spock1
A0A0G2K463	Pde4dip
D3ZAQ0	Fundc2
A0A0G2KAN5	Erh
A0A0G2K1J9	Slc2a2
D3ZDR1	Fam193b
Q3KR55	U2af1
A0A0G2K8E6	Vapa
B3DMA1	Atxn2l
D4A315	
Q6P762	Man2b1
D3ZFM1	Asf1a
Q5XIQ6	Trmt2a

D4A0L4	Ybx3
B2RYB3	Srrm1
Q5M949	Nipsnap3b
B2GUW7	LOC103693457
A0A0A0MXX7	Fxn
Q6P505	Psma4
E9PTI6	Raly
D3ZCQ7	Zcchc17
D4ABK7	Hnrnph3
A0A5D0	Cd200
Q7M0G5	
A0A0G2K0X4	Rac1
A0A0G2JU92	Emb
A0A0G2KAZ7	Hnrnpdl
D4ACM9	Mfap1a
G3V7R1	Npap60
D4A2G9	Ranbp1
A0A0G2K2B4	Ncor1
B5DF60	Eif1ax
A0A0G2JWG6	Golgb1
G3V8W2	Cds2
Q64LD0	
D3ZS22	LOC310177
A0A0G2K707	Dgkz
A0A0G2JU73	Trip12
D4AE50	Nfx1
Q63287	RGD1560099
A0A0G2JXK7	Fbxo7
D3ZE72	Metap1
D3Z8N9	Asxl2
A0A0G2K7S2	Ints6
M0R3V4	Mydgf
D3ZF21	Gprin3
Q5XI86	Ptrh2
D3ZI68	Prpf31
B5DFH8	Cstf2t
D3ZZE3	C1H10orf76
D3ZX38	Pfdn1
M0R660	
Q32PZ0	Pak1ip1
A0A096P6L8	Fn1
D4AD89	Sez6l
D3ZDJ6	Vstm2l
F1M787	Ctnnd2
Q45QM8	Gnai3

A0A0U1RRY5	Rpl3
A0A0G2JTA0	Faf2
A0A0A0MXV1	Eps8
M0R3N4	Vat1l
D4A0G7	Rab37
D4A511	Srp9
A0A0G2K9H8	Arf3
D3ZUY0	Rdh14
F7IXA2	Ring1
A0A0G2K7G7	08-sep
D3ZWU1	Brd3
A8ASI9	Bid
Q5XI77	Anxa11
Q5U2X8	Acot9
Q5M830	Rfc3
G3V9X2	Gpsm1
Q4KM71	Sfpq
Q9JMB3	Epb41l3
Q6AZ25	Tpm1
F1LNG5	Pik3r1
D4A068	Pnma2
D4AA35	Asmtl
A0A0H2UHI2	Map2k1
A0A0G2K6G2	Apmap
B2GUW2	Arhgef16
D3ZKK4	Zfp869
D3ZVS2	L2hgdh
A0A0G2K0X1	Pcm1
B4F764	Nudt21
Q6P503	Atp6v1d
F1M9Q8	Agps
A0A0G2JWA8	Macf1
Q45QJ4	Plcb3
D3ZZ62	Xpot
B5DFK8	Pdxdc1
D3ZC56	Dst
Q3ZAU6	Rnf14
A0A0G2K590	Spast
G3V7N4	Syk
D3ZLS5	Hectd1
Q4QRB8	Asl
G3V786	Akr1b8
F1M4W7	Cstf3
F1M0N1	Abl2
F1LQH2	Nfkb1

M0R766	Tvp23b
A0A0H2UI21	Crat
A0A0G2K1E5	Sympk
Q3B7D2	Sptlc2
D3ZTR5	Zbed5
G3V8U9	Psmb4
M0R809	Gng4
D3ZLX3	Mob2
Q76MV3	Cox17
A0A140TAA4	Pdcd6ip
F1M1R4	Rbm27
G3V8C4	Clic4
E9PU01	Chd4
M0RDJ3	Eif2ak2
M0R4V3	Snap23
Q2PYT3	
D3ZLU0	C2cd4c
G3V774	Fbxo2
D4AEP0	Adss
O08814	Rap1b
A0A096MJP4	Cep41
Q4V8J6	Ythdf1
A0A0G2JSU7	Mef2d
D3ZNV5	Pdzrn4
D4A7L6	Rpia
Q68G41	Eci1
Q6P6Q5	App
Q3MHT2	Nfs1
D4ADD7	Glrx5
Q3MID6	Calu
Q5U2T9	Fkbp5
D3ZCG2	Kif21a
D3Z9P1	Kdsr
B1WBQ0	Cdc5l
D3ZBM3	Fech
D3ZZ38	Snx18
A0A0G2KA25	Tm9sf4
A0A0G2JVC8	Lss
G3V7Z8	Pabpn1
A0A0G2JXY9	Epn2
A0A096MIT8	LOC100912195
D3ZP59	
Q5XIA5	Coasy
M0R6D6	
D4A997	Htatsf1

F1LVX2	Ehbp1
A0A0G2JZG7	Sars
A0A059NZR0	Hn1
D4A0F5	07-sep
A0A0G2K4N7	Mpp6
G3V7Z3	Nol3
F1MAK9	Smpd4
Q6AYU2	Pcbp2
Q7TQ90	Adh4
A0A140TAI8	Ahcyl1
G3V6Z7	Golga5
A0A0G2K930	Rab7a
D3ZP49	LOC100910678
Q5XIN4	Mtmr9
F1LST0	Heatr5b
B2RZ44	Naa20
A0A0G2K6A2	Ptpn9
Q4FZR7	Spg20
D3ZHV7	Sdsl
B2GVA1	Selo
A0A0G2K8P5	Stmn3
A0A0G2K6D0	NEWGENE_1565481
A0A0G2JSH5	Alb
D4A2D3	Mycbp2
B5DEL5	Klhl9
D3ZL77	Eif3h
D3ZHW0	Dhx29
Q99521	Hadha
A0A0H2UHZ4	Zranb2
D3ZXL1	Arih1
A0A0G2K1M8	Prep
P70625	ZO-2
G3V656	LOC100910540
A0A0G2K675	Fto
A0A0A0MXZ9	Dapk3
F1LMC3	Camk2g
A0A0G2JVM3	Zc3h7a
B1WC34	Prkcsh
D3ZSZ6	Dgkh
B0BMU2	Nop16
G3V8L1	Pycard
A0A0A0MY07	Usp15
F1M656	Tulp3
D4AC97	Emsy
D4A533	Tapt1

Q6IN22	Ctsb
D3ZFQ1	Atxn7l3b
D3ZU64	Vps37b
A0A0G2K866	Abcb7
A0A0G2JVM2	Mia3
G3V6A2	Slain2
B0BMZ1	RGD1305587
O54857	Pten
A0A0H2UHG0	Yars
F1M835	Cbr4
A0A0G2JWC7	Fermt2
A0A140TA98	Mta1
Q2XTA4	Fabp5
D4A500	Hddc3
D4A0Y4	Oxnad1
Q6QI75	Wdr55
Q7TPI7	LOC100911453
D4A7U1	Zyx
D4AAN8	Helb
F1LMN8	Khdrbs3
D3ZQL1	Emc7
G3V7I3	Atp13a1
A0A0G2K5F1	Macrod1
D4A7J8	Prpf4
G3V6R5	Suox
A0A0G2JSS1	Ilkap
A0A0G2K3Q5	Nrcam
A0A0G2K7L0	Mrps10
F1M775	Diaph1
Q0QF43	Mdh2
D4A3M7	Bsdc1
G3V640	Timm44
A0A0G2K8N6	Ankmy2
A0A0G2JUW8	Lnpep
B0BNL2	Pin1
A0A0G2JZD1	Ppp2r5c
F1LPD6	Acaa1b
D4A8N1	Dpm1
Q4QQV0	Tubb6
A0A0G2K1A8	Plekha1
A0A0H2UHN2	Idi1
M0R7K1	Lin7a
D3ZZV1	Pam16
M9MMM8	Brsk2
G3V946	Dpy30

A0A0G2JSQ1	Sncb
B2GV14	Txlna
D4A3V4	Rnf214
D3ZLH8	Rtf1
B0BNM2	Mbd2
A9LRT4	Jnk1
Q642E5	Mvd
G3V7B0	Nol9
A0A0G2K5C8	Stim1
D3ZN27	Dnajc13
A0A0A0MXX0	Cd2ap
D3ZKQ4	Rabl6
B1WBV6	Aplp1
Q5PPJ6	Lars
Q3SWT7	Nrbp1
F1LYA4	Arid4b
A0A0G2JYS8	Ppp1ca
G3V9L1	Tex2
A0A0G2JWM2	Sirt2
Q66HF3	Etfdh
A0A0G2JSS9	Atl3
D3ZD73	Ddx6
M0RBL8	Tceal6
G3V992	Gtf2e1
A0A0G2K5Q2	Crnkl1
A0A0G2K2P6	Hbs1l
G3V728	Nipsnap1
A0JN30	Cnpy2
F7EXQ7	Ndufa8
D4AEK9	Ccdc6
D3ZPF2	Mcat
A0A0G2JSR0	Vdac3
A0A0G2JTR4	Abr
A0A0G2K5Y1	Vipas39
A0A0G2K4R1	Ppp1r12c
M0RAY0	Dus3l
Q6IRJ8	Arfgap1
F1LNT8	Vamp8
M0RAI4	Ears2
B2RYS9	Trmt112
D3ZIP8	Endod1
G3V647	Pdxk
Q497A9	Eif4ebp2
M0R5H1	Etl4
A0A0G2K7Q8	Kif1b

A0A0H2UHM0	Vps28
F1LS86	Iars
F1LRE1	Gsr
G3V652	Gpn3
D3ZIE4	Fyb
R9PXS4	Pitpna
F1LRI6	Taok3
D3ZXK4	Abhd11
B1H223	Dscr3
A0A0G2K214	Zc3h7b
D4A3I4	Btf3l4
E9PTG5	Plekha5
D3ZQN7	Lamb1
A0A0H2UHY0	Vars2
A0A096MK91	Ak6
G3V9V3	Dync1i2
D4A5W9	Snap25
B2RYL4	Armc6
D4AE02	Fam98b
D3ZGN7	Mical3
D3Z8W0	Paox
Q66SY1	Picalm
A0A0H2UHV7	Aars2
Q3LVE5	Snca
B2RYA8	Dnajb2
Q6TUH4	Thoc1
Q5M7X1	Copb2
H9KVF3	Stk24
B0BMV1	Set
A0A0H2UH94	Gcc2
Q4KLL7	Vps4b
B0BMX0	Tesl
M0RDC5	Dbi
B2RZB7	Snrpd1
F1LWN1	Ncoa7
D3ZP13	Qsox2
G3V7V5	Fkbp11
G3V702	Smu1
D3ZE73	Smc1b
D4AE08	Fam206a
D3ZF97	Erlec1
Q68FX8	Pmpca
D4AAZ8	Irf2bp1
A0A096MIX2	Ddx17
F1LQ55	Scp2

D4A040	Mrps11
A0JPL9	Rem2
Q7TP24	Srprb
Q6AY07	Aldoart2
D4A2D7	Ipo4
A0A0G2JX56	Dnajc5
B5DEJ9	Sbf2
B8YDD0	Hax1
Q6B437	Hsp90aa1
A0A0G2KAS8	Wnk2
B2RYL8	Ddx41
D3ZBS9	Smarcd1
A0A0G2JY96	
D3ZAR8	Otub2
A0A0G2K1Z9	Hm13
D3Z9D0	RGD1306271
A0A096MJQ1	Arpc1a
A0A0G2K1F2	Acacb
D4A772	Dtna
A0A0G2K3L9	Ppfia1
D4A3S8	Nsun2
A0A0G2K1I5	Cnrip1
D3Z8C7	Wdr48
D3ZT07	05-sep
Q6AYS3	Ctsa
F1MA36	Sptbn2
Q68G16	Ppp5c
D4A5K6	Zmpste24
A0A0G2JU43	LOC100361920
D4A099	Nsun4
D4A416	Clptm1l
D3ZU13	Eif4g1
F1MAQ4	Trappc10
B4F759	Phf5a
Q2M2S1	Tor1b
A0A0G2JTD1	Rprd2
A0A0G2K9L6	Rbck1
F7FKL9	LOC100911725
G3V8H5	Ikbkb
D4A417	Pcif1
E9PU02	Trappc9
F1LNT0	Dpysl4
D3ZHK4	Rb1cc1
D3Z941	Mars
A0A0G2K3J4	Sphkap

B5DFA5	Kdelc1
A0A0G2K7C1	Bag6
F8WFM2	Napb
V9GW19	Kdm5c
D3ZE17	Caskin1
A0A0G2JZ84	Epg5
D3ZSU7	Mien1
A2VCW7	Usf2
D4AA63	Ubqln2
A0A0G2JW14	Spg21
E9PSU5	
A0A0G2K9F2	Nemf
D3ZAK6	Rps15-ps2
A0A140UHX9	Ccz1b
G3V6L8	Strn
Q4KLN3	Srpk1
D3ZUL1	Ccdc124
F1LP26	Shroom3
M0R7Y9	
F6T1W7	Pold3
D4A6P3	Shtn1
A0A0G2JT47	Snx16
A0A0G2K7N7	
D3ZF12	Spcs3
F1LV44	Tenm3
D4A2N2	Inpp5b
Q7TP51	Ube4a
D4A7I8	Sumf1
F1M6K4	Phf21a
F1LT49	Lrrc47
G3V779	Lad1
B5DF29	Rfc5
D4A3E3	Arid1a
B1WBN5	Pus1
A0A0G2K235	Rab1a
D3ZSC8	Dnajc17
A0A0G2K9E5	Lars2
Q6P9W2	Araf
D3ZYL0	Ripk1
B2RYK4	Slc25a46
G3V7M0	Cnot1
D3ZJB8	Arih2
M0R907	Snrpd3
D3ZVB3	Fam135a
G3V734	Decr1

G3V8D5	Pgls
A0A0H4SRI2	Kidins220
F1M8K0	Dag1
Q6P757	Eef2k
A0A0G2K2V5	Exoc1
G3V7G9	Eif3l
A0A0G2JSY3	Nln
Q63629	
G3V8V4	Cers2
A0A0G2K931	Psat1
A0A0G2K093	Hspa13
Q9JJS4	Slc4a2
D4ADZ2	Bicd1
B0K010	Txndc17
G3V6S1	Pawr
F1LZI1	LOC680121
F1LNL0	A1cf
A0A0G2K1F3	Copg1
A0A0H2UHW2	Coq4
B0BNE3	Trappc5
D4AB01	Hint2
A0A0H2UHU0	Rps25
F1LQ35	Pde3b
G3V8C9	Ncoa6
D3ZCP9	Gtf2e2
B5DEL2	Cpsf1
F1LU48	Ergic1
P97563	Tmsbl1
F1MAF5	Pcid2
A0A0G2K0X5	Abhd16a
G3V7G0	Dync1li1
D3ZWF5	Eny2
A0A0G2K0P9	Copb2
D3ZI16	Cops6
A0A0G2JXS9	C1qtnf1
A0A0G2JXG7	Lrrc16a
D3ZJT4	RGD1564469
G3V997	Dcx
A0A0G2KAT3	Rpa1
A0A0G2JXU2	Wbp11
A2VD09	Abi1
D3ZHG2	Klc1
B2RYP8	Tubgcp2
A0A0G2JTN4	Pfas
Q4V8H2	Exoc1

A0A0G2JTA1	Ppp2r5e
A0A0G2K0V8	LOC100911422
F1LQZ3	Kif3a
Q6AY90	Fam192a
D4A1H2	Plcxd3
F1LND1	Cog1
D3ZUX7	Acsf3
B1WC02	Ctps1
Q99PW1	Yes1
D4ADF6	Zfyve16
D3ZVU7	Hdac1l
G3V8R0	RGD1311703
D4AB26	Smc6
A0A0G2K6A9	Rufy3
B1WBQ5	Stk3
F1LT58	Kpna6
F1M7V6	Cadm4
Q68FS9	Commd10
A0A096MK73	Stmn1
D3ZLX2	Borcs7
D4A820	Cyp2s1
D4ACL2	Ttc38
A0A0G2K5W6	Lyar
D3ZWS0	Scrib
F1LQI5	Ubr4
G3V8U6	Pola2
A0A140TAC3	Epn1
G3V6R9	Bloc1s4
F1M6T6	Ppp6r2
A0A0G2K5H2	Clvs1
D3ZFJ3	Sh3bp1
A0A0G2JYY7	Rbfox2
B1WBX6	Smap2
F1LPP2	Tlk2
A0A0G2JZ48	Tmf1
D4A017	Tmem87a
D4A4R1	Eml3
B2RZ82	Pcgf2
E1AZB1	
D3ZF50	RGD1310335
M0RD20	Capns1
Q6IN03	Rab28
A0A0G2K9L2	Tom1l2
Q499R6	Zfyve19
A0A0G2K1P1	Mtmr7

A0A0G2K8E5	Ints10
Q5RK25	Pmm1
A0A140TAA3	Ralgapa1
G3V8T4	Ddb1
F1LR87	Hexb
A0A0G2QC33	Atg4b
A0A0G2QC21	Arhgef7
A0A0G2JY24	
A0A0G2JX45	Ate1
F1LM47	Sucla2
G3V8U2	Keap1
D3ZGW2	Ap1g2
D3ZUB0	Rcn1
B0BNA3	Rars2
Q7TPK0	Fdps
A0A0G2JZ53	Birc6
A0A0G2K1A0	LOC102553099
F1M9G7	Crebbp
M0RDN9	Usp19
A0A0G2JXW4	Hnrnpc
A0A0G2K9Q1	Golph3l
F1LSX8	Atp2b4
A0A0G2K1S9	Ccm2
A0A0G2KAE2	Tusc3
D3Z9E6	Cpsf2
F1LSP6	Ptpn2
B2RYC5	RGD1310209
A0A0G2K8W9	Sptbn1
A0A0G2K4D9	Stag2
R9PXU0	Tmpo
F1LZF2	Fnbp1
Q498M7	Cnot4
Q6VEU8	Ddx24
Q498C9	Zfp207
A0A0G2K012	Ubxn6
Q1PBJ1	Mfge8
F1LR38	Abcd3
G3V766	Nbn
M0R3M8	Rrp12
A0A0G2JU07	Ube2v2
A0A0G2K167	Rmdn1
D3ZPD0	Ckap2
A0A0G2JU11	Rapgef2
F1M951	Ptpn23
M0R8C5	Eri3

W0T3G7	Map7d2
A0A0G2K3M6	Atp9b
A0A0G2K7G2	Agfg2
F1LNE5	Memo1
G3V7U8	Arpc5l
Q8CHJ4	Yy1
F1LPG9	Fam21c
F1LP34	Anp32b
F1LYZ8	Ppp1r21
A0A0G2K9F7	Zmynd8
Q5U313	Ankrd13a
F1M3H8	Hnrnpa0
A0A0G2K248	Mat1a
Q6LE97	
A0A0G2K743	RGD1562415
D3ZBC7	Dhrs13
A0A140TAB1	Pde4d
F1LR78	Ssh3
A1L1J8	Rab5b
D3ZFX4	Pgm3
D4A3X1	RGD1308601
A0A0G2JUX5	Purb
D3ZN95	Hcfc1
D3ZWS6	Naa30
D3ZG78	Zzef1
A0A0G2JVF1	Rundc3b
B2RYR9	Dynlt1
G3V6G7	Pnpt1
D4ADS8	Rab4a
Q8CJC8	Ppp1r17
D3ZX13	Kif20b
D4AC16	Tbc1d1
Q6MG75	Nelfe
Q6AXW2	Tmod3
F1LMY6	Sugt1
A0A0G2K3H2	Dock7
A0A0U1RRR6	Aimp2
A0A0G2JYV9	Crem
B2GV57	Cars2
D4AD70	RGD1561636
G3V927	Dlgap4
F1M8T4	Crybg3
Q6TUH0	Pde12
G3V926	Chuk
Q4G079	Aimp1

F1LP81	Esrp1
D3ZWL6	Ahcyl2
G3V796	Acadm
D4ABN8	Xrn1
F1M771	Rybp
F1M9V7	Npepps
G3V6W7	Cpsf3
Q642E3	Cdk5rap3
A0A0G2K0Q2	Kif5a
A9CMB5	Ubxn4
D3ZG07	LOC100360750
A0A0U1RRW0	Srr
A0A0G2KAW7	Eif4h
G3V8C6	Sra1
D3ZVU6	Avl9
Q45QN0	Gnai2
G3V7L8	Atp6v1e1
A0A0H2UHM5	Pdia3
G3V8Q2	Ina
F1M0X6	Magohb
B4F779	Appl2
F1LRD7	Pou2f1
A0A0G2JUI5	Clasp1
Q9R019	Chek2
A0A0G2JZA2	Grpel1
D3ZF11	Lamtor5
F1M1Y1	
A0A0G2JT06	Gps1
D3ZAP7	Chd7
B0BNJ0	Sin3b
A0A0G2QC04	Pls1
F1LUT4	Atp8a1
Q5HZY3	Uchl5
F1LQP9	Tnpo1
B5DEY8	Snx6
D3ZYH3	Nudt10
Q5XIC6	Psmd12
F1LU71	Auh
B4F775	Gopc
E9PTX3	Hook2
B4DQ80	
A0A0U1RRZ5	Prrc2a
B0K015	Commd8
A0A0G2QC39	Txlng
D3ZQB6	Cecr5

D3Z8J0	RGD1564425
G3V7C6	Tubb4b
A0A096MK24	Morc4
B2GV97	Sepsecs
D3ZP15	Rab9b
F1LQN3	Rtn4
Q99PF3	Apex1
A0A0G2KAM8	Vprbp
F1LUI2	
G3V7P2	Fgl2
A1A5L2	Pgm1
A0A096MJ27	Mdn1
Q7TP07	Vps13a
D4AE75	Pop1
D3ZSB7	Rps6ka4
G3V8F7	Gga2
D3ZF45	Larp4b
Q5BJT9	Ckmt1b
F1LSM0	Usp24
F1LR42	Rufy1
A0A0G2JZ83	Agap3
D3Z8E0	Rps6ka3
F7EMB2	RGD1304704
Q5XIV1	Pgk2
D3ZTP6	St18
A0A0H2UHE5	Ppm1a
B2GV72	Cbr3
Q5M964	Fh
G3V982	Elmo2
A0A0G2JZE8	Toe1
A0A0G2KA14	Clec16a
Q9JKD5	F11r
D4AC12	Anks1a
D4ACL8	Atm
D4ADT3	Wapl
Q5D059	Hnrnpk
D3ZSV7	Thumpd3
F1LT62	LOC100909677
G3V984	Bsn
B2GV55	Ube2q1
D3ZA84	Tln2
B5DFD8	Sh3bgrl
A0A0H2UHS4	Dpf1
A0A0G2JVC2	Scyl2
M0RCN8	LOC102546892

A0JPN0	Rad18
A0A068FP44	CARM1
F1LPS8	Pura
D3ZCS3	Pcbp4
B4DL66	
D3ZC98	Ccnt1
Q9WUW2	Vamp2
A0A0G2JXJ7	Esyt1
Q5M7T6	Atp6v0d1
G3V879	Coq7
A0A0G2K8F6	Man2b2
B4F772	Hspa4l
G3V8G1	Agtpbp1
Q497A2	Slc35b2
G3V9U0	Acss2
Q3HSE5	Akt2
G3V9N6	Yif1b
A0A059NZV6	Hn1l
D3ZV82	LOC685067
A0A0G2KB56	Gfpt1
M0RBN6	Nrxn1
F1M4J0	Rictor
Q66HM7	Ssb
C7E3F3	Ddx4
A0A0G2JYW3	Clta
A0A0G2K4L3	Fam134b
R9PXV8	Wrnip1
D4A5I4	Nav1
B5DF55	Stam
F1MA42	Sez6
Q6MGC4	Pfdn6
G3V6C4	Ugdh
Q80ZF4	Mapkapk2
Q4KM57	Lemd2
M0RBS9	Snx27
D3ZWQ8	Arhgef6
F1LS70	Abcc8
Q6TXJ6	Cyld
B2RYN6	Ap1g1
C6L8E0	Dbn1
A0A0G2JY43	Aldh3a2
G3V7V9	Lcmt1
Q5HZA7	Bin1
A0A0A0MXZ8	Impdh1
D4AAE9	Cisd2

B2RYI8	Papss1
D4A1G1	Acyp2
B2GUZ9	Fam49b
A0A0G2JV54	Ptbp3
D4ADS6	Ints7
A0A0G2K4Q9	Igfbp3
D4A0V6	Anln
A0A0G2K266	Ufl1
D3ZDB9	Nmral1
D4A644	Map7d1
F1M7P4	Prph
D3ZJR1	Eps15l1
F1LRZ7	Nefh
D3ZZ94	Parp10
A0A0G2K0T2	Cd63
D3ZHA7	RGD1560334_predicted
M0RB67	Ppidl1
D4A8H5	Ppp4r2
A0A0G2K242	Zfp516
G3V834	Prrc1
Q6IMX3	Acads
F1LVV4	Rcc2
A0A0G2KAX0	Psme4
B2GV93	Akap8
D3ZTL0	Tcerg1
A0A0G2JSG6	Ak2
B0BNK1	Rab5c
D4AAH9	Tbc1d23
B0BN46	Grhpr
A0A0G2K2D6	Tppp
B1WC84	Cnpy4
R9PXV7	Ppp1r7
D3ZR49	Man1a2
A0A0G2JTT2	Picalm
D3ZH75	Akt1s1
M0R7D1	Larp7
D4ABT4	Ndor1
D3ZYE5	Fam172a
A0A140TAE1	Fads2
G3V9S0	Cyb5r1
Q6AYB2	Sphk2
F1LRP3	Pias2
D3ZJR6	Smcr8
A0A0G2K4J0	Arfgap3
M0R544	Gaa

F1LXJ9	Ptprt
Q0D2L6	Rragc
D3ZHJ6	Atp2a3
A0A0G2K8M1	Zfp84
F1LQA8	Mdc1
A0A0G2K1E7	Sptan1
A0A0G2JTK4	Ppp6r1
D3ZXI2	
F1LPV0	Nars
Q99MI5	LOC100912604
B1WBN4	Rexo4
D4A4A9	Mrpl19
A0A0G2JW90	Pdha1l1
A0A0G2JZ52	Hnrnpu
A0A0G2KAN7	Gls
A0A0G2JXP1	Gyg1
E9PTB6	Polr3a
A0A0G2K351	Unc119b
D3ZDX7	Mrpl48
H7C5X3	RGD1566078
A0A0G2JTP7	Rnf2
B2RZC6	Ilf2
F1LP07	Dph6
Q642E6	Tpp1
Q6P2A7	Flot1
B4F7A9	Csnk2a2
B1H282	Colgalt1
D3ZUJ7	Ankrd13d
G3V7N5	Cpt2
Q5PQV3	Atp1a4
Q1PS21	Mcm7
D3ZU88	RGD1309104
A0A0G2K0S7	Rap1gap2
D3ZCA0	Prosc
F1M324	Plcl2
D3ZSW9	LOC100910755
Q5BK95	Gdi1
F1LNX7	Tssc1
D3ZV75	Mfsd1
A0A0H2UHJ5	Zfr
M0R965	LOC685025
A0A0U1RRV5	Cluh
Q4KLI4	Ppil1
A0A0G2K9T1	Itch
A0A0G2K8D1	Mospd2

D3ZER6	Tnpo2
A0A0G2JV16	Dock11
A0A0G2JV51	Nat10
Q6LCA5	Prkar1a
M0RD63	Ranbp3
A0A140TAH5	Ap2m1
D4A1X2	Exosc10
F1M842	Tp53bp1
D4ADG2	Iba57
D4AEC0	H2afv
F1M7B8	Ube3a
B0K017	Adprhl2
A0A0G2K950	Papss2
D4A5V8	Mapk9
F1LQI1	Hagh
A0A140TAB9	Pcmt1
F1LPH1	Cast
B3DM95	Ptms
F1M9X5	Prkar1b
A0A0G2JUF2	Snx30
D3Z8M8	Tsc22d1
Q99ND8	Ppm1b
G3V8R1	Nucb2
A0A0G2K4T7	Gtf2i
F7EV94	Bckdha
Q5FVS6	Pik3r2
F1LPS3	Nolc1
A0A0G2JTK6	Mta3
Q5RKJ4	Fnta
M0R851	Usp4
Q68G33	Gorasp2
G3V940	Coro1b
Q3ZB97	Ap2b1
D3ZQM3	Itga3
A0A0G2JU05	Llgl2
G3V9X6	Rad50
A0A0G2K330	Hadhb
D3ZXB8	Etnk1
A0A0G2K1T0	Gls
E9PSS1	Dclk2
F1LR76	Hexdc
D3ZZQ6	Ints4
A0A0G2K6D5	Pitrm1
F1LMZ4	Gfm2
F1MAC0	Ifi47

Q9QWQ8	Ptpn11
A0A0G2QC19	Dnajc21
M0RAD5	Clpp
B6DYQ9	Gstt2
F1MAA5	Rangap1
A9CMB8	Mcm6
F1LM33	Lrpprc
D3ZQM0	Sf3a1
A0A0G2K9F9	Coro7
D3ZZC1	Txndc5
A0A0K2TVC4	Actr2
D3ZXK9	Pnp
F1M5R3	Abhd14b
B5DEQ4	Snrpb2
A0A0G2K5Q8	Clk2
A1L1K6	Gtf3c5
D4ACK1	Nup214
A0A096MK13	Pola1
F1M124	Cobll1
Q68FQ5	Mtif2
F1MA61	Ncoa2
D4AEC2	Camsap2
Q5U2Q5	Rrm1
E9PTB2	Supt5h
F1LRB8	Mat2a
H9KVE3	Ubr5
A0A0G2K051	Eea1
Q5XIJ3	Idh3g
B2GUX3	Mcm5
D3ZFP4	Mcm3
D4A4T0	Stub1
D4AEI5	Myef2
Q6P136	Hyou1
A1A5S2	Cxxc1
D3ZDT1	Epb41l2
F8WFR8	LOC100359876
F1LPK7	Pls3
D3ZJ01	RGD1307235
A0A0G2K7Q7	Ide
A0A0G2K9V6	Tars
A0A0G2JYI0	Lrba
Q4QQS7	Umps
B2RYF8	Cnpy3
D4A6C5	Arhgap1
A0A0G2K2C7	Usp9x

F1M7L6	Scg3
A0A0G2JUC7	Dctn2
M0R9X8	Dync1h1
Q5XIT4	Nub1
F1LN91	Tns3
M0R781	Dpp9
D4A8U7	Dctn1
A0A0G2JSJ2	Cmpk1
G3V7W1	Pdcd6
Q32KJ5	Gns
G3V7L9	Luzp1
M0RAT6	Tmed8
A0A096MJX5	RGD1305178
B2GVB7	Apeh
D5MTG9	Ubr1
F1LML7	Hip1r
E9PSJ3	Kif4a
G3V7L1	Utrn
A0A0G2K089	Dennd4c
D7NIW0	
F1LT36	RGD1564698
D3ZAI6	Nt5dc3
A0A096MJA0	Tcp1
A0A096MJB9	Eif2s3
F1LN69	Mink1
B1H257	Borcs5
D3ZVH2	RGD1560831
F1LQT9	Dnmt1
F1LTW9	Efr3b
B4F7C2	Tubb4a
M0RDJ4	Gmfb
A0A0H2UHX3	Rps4x
Q62819	Ddc
Q5U322	Cpe
A0A0G2JVS2	Pa2g4
B2RZD4	Rpl34
D4A9N5	Trim25
G3V928	Lrp1
A0A0G2K9P4	Sh3glb2
Q8CHN7	Pcp4
Q1KQ07	Stat6
Q0QER8	Idh1
Q566D8	Lig1
F1LXF1	Bcr
Q14TE9	Top2b

B2RZ68	Dcaf7
G3V9G4	Acly
A0A0G2JZA7	Tmem2
F1LQ09	Atl2
B5DFA0	Vil1
B3GS92	
Q76LU9	Gpx4
D4A1V7	Mob1b
A0A0G2JSM8	Cdc42
Q5RK10	Rpl13a
Q3T1H3	Ncam1
M0R7I0	LOC100359600
A0A0H2UH99	Rpl24
A0A0G2K2B3	Khsrp
Q6P6T6	Ctsd
F1LPK1	Il6st
Q52KJ9	Tmx1
D4A352	Myrf
D4A0W7	Fndc3b
A0A0G2JSQ0	Ppp2r2d
A0A0G2K5S2	Recql
D3ZYS7	G3bp1
A0A0U1RVI9	Rps2-ps6
F1M0K6	RGD1565566
D3ZAP9	Gpd1l
A0A0A6YYM0	Acsl6
A0A0G2K8V3	Thoc2
M0RB65	LOC100364509
M0RCY2	LOC683961
M0R6E0	Atp9a
G3V9Y1	Myh10
M0R715	Pnpla6
D4ACN6	Col4a3bp
A0A0G2JVA5	LOC100910245
D3Z9H2	Hapln4
D3ZAA9	Mpp2
F1LPV2	Vldlr
M0RBX7	Aplp2
Q499T3	Sirpa
F1M885	Myo7b
D3Z8Q7	Fam96b
F1LMY3	Ptprz1
Q63575	Tgoln2
M0R919	Vbp1
B0K031	Rpl7

G3V9R0	Luc7l
D3Z8U5	Tll1
A0A0G2JY08	Myo18a
A0A0G2K954	Nabp2
A0A0G2JVG4	Pecr
F7EPE0	Psap
D3ZFF4	Lclat1
D3ZW09	Sorcs2
D3ZC82	Nufip2
A0A0H2UHF3	Qtrt1
A0A0G2K654	Hist1h1c
D4A9Q3	RGD1563570
D3ZBZ6	Disp2
F1LWE6	Msi2
D4A412	LOC688684
D4A3D8	Xpc
M0RD99	Rpl30l1
G3V817	Xrcc5
A0A096MJG7	Nebl

Supplementary Material 5

Zip14 siRNA Control

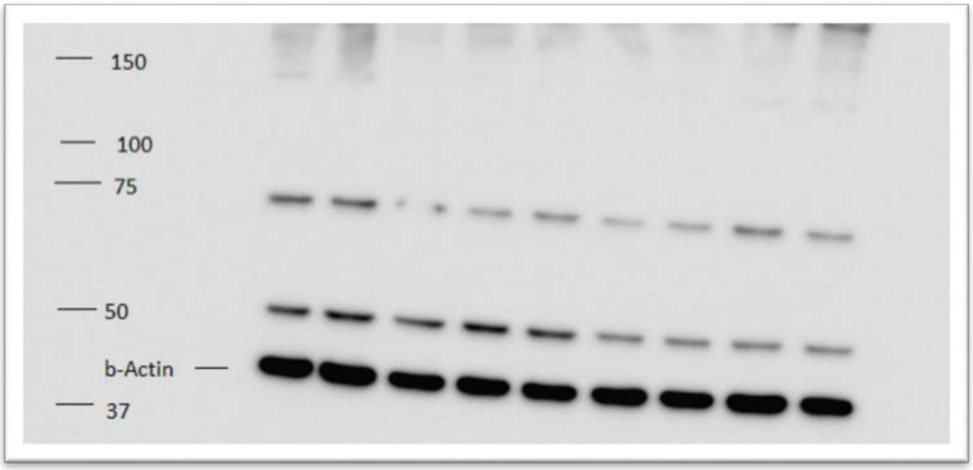

Chga precurser

Chga 50 kda

Supplementary Material 5. Western blotting of Chga (Chromogranin a) and B-actin (internal control).

Line 1-5; Zip14 siRNA-treated samples. Line 6-9; non-targeted siRNA-treated samples (Control). 5 μg of protein were used.

Supplementary Material 6

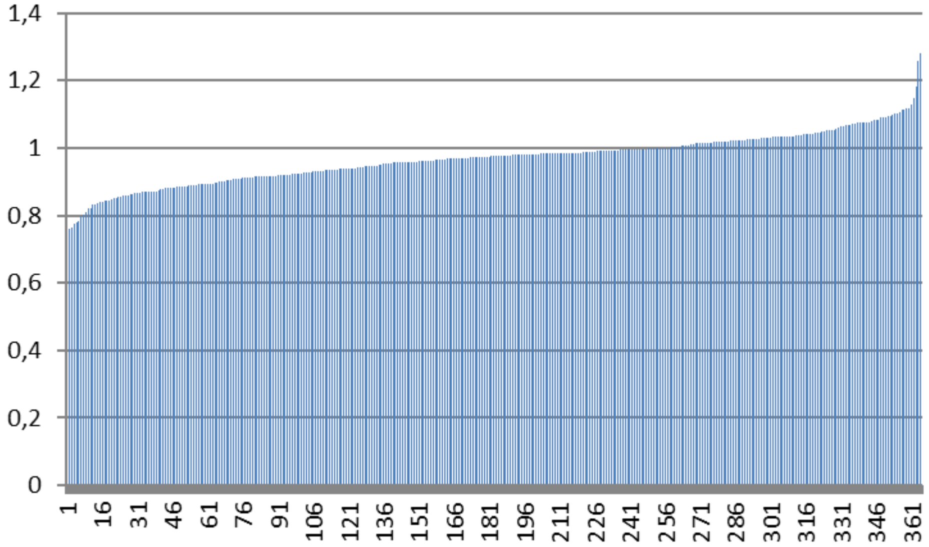

Supplementary Material 6. Quantitative ratio of mitochondrial proteins in Zip14 siRNA vs. non-targeting siRNA-treated samples (Control siRNA). Out of the 3431 proteins quantified in the samples, 365 proteins were identified as mitochondrial. The quantitative ratio between average Zip14 siRNA and non-targeted siRNA-treated samples are shown of the 365 mitochondrial proteins. The median ratio was 0.98.

Supplementary Material 7

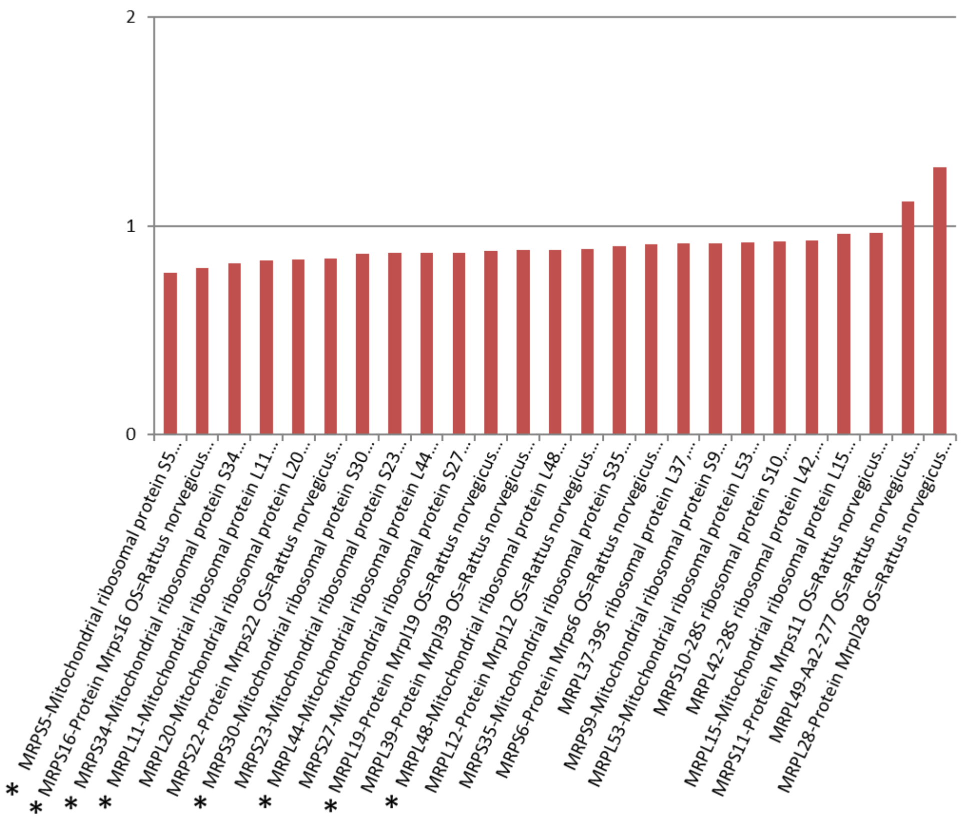

Supplementary Material 7. Quantitative ratio of ribosomal mitochondrial proteins in Zip14 siRNA vs. non-targeting siRNA-treated samples (Control siRNA). The median quantitative ratio of the depicted ribosomal mitochondrial proteins was 0.89. * denotes proteins with $p<0.05$.

Supplementary Material 8

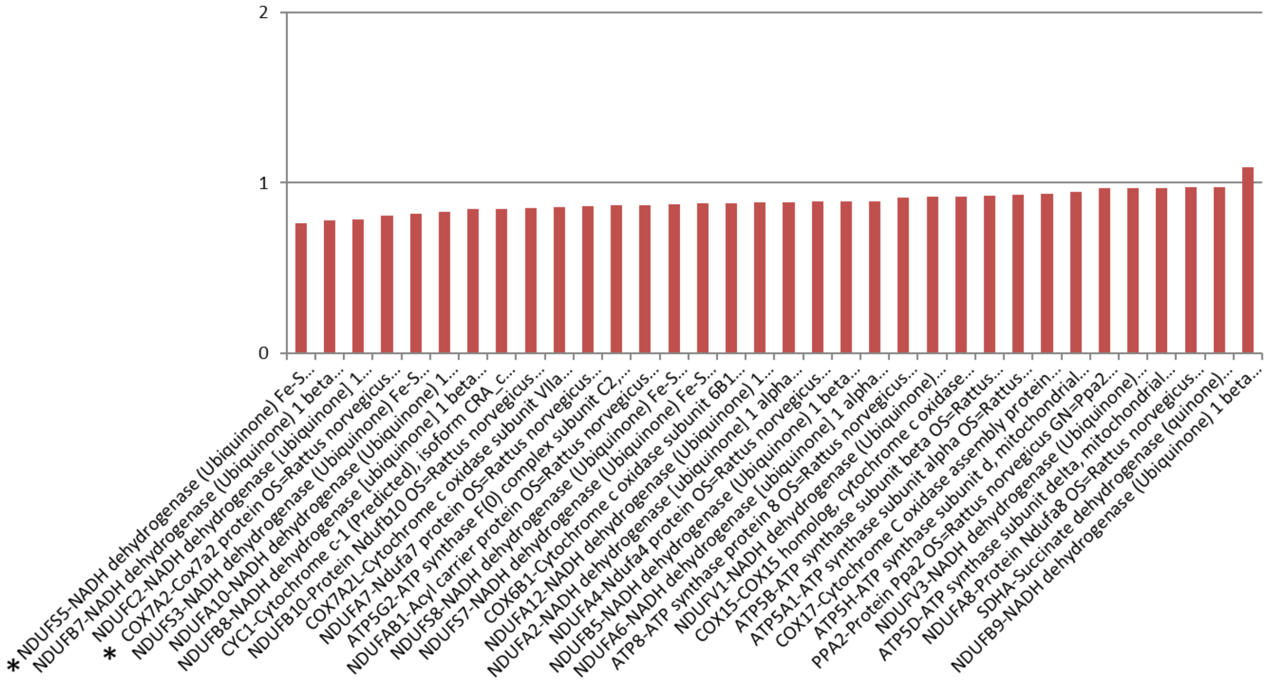

Supplementary Material 8. Quantitative ratio of mitochondrial proteins involved in oxidative phosphorylation in Zip14 siRNA vs. non-targeting siRNA-treated samples (Control siRNA). The median quantitative ratio of mitochondrial proteins involved in oxidative phosphorylation was 0.89. * denotes proteins with $p<0.05$.
